# TumorBoost: Normalization of allele-specific tumor copy numbers from a single pair of tumor-normal genotyping microarrays

**DOI:** 10.1186/1471-2105-11-245

**Published:** 2010-05-12

**Authors:** Henrik Bengtsson, Pierre Neuvial, Terence P Speed

**Affiliations:** 1 Department of Statistics, University of California, Berkeley, USA; 2 Bioinformatics Division, Walter & Eliza Hall Institute of Medical Research, Parkville, Australia

## Abstract

**Background:**

High-throughput genotyping microarrays assess both total DNA copy number and allelic composition, which makes them a tool of choice for copy number studies in cancer, including total copy number and loss of heterozygosity (LOH) analyses. Even after state of the art preprocessing methods, allelic signal estimates from genotyping arrays still suffer from systematic effects that make them difficult to use effectively for such downstream analyses.

**Results:**

We propose a method, TumorBoost, for normalizing allelic estimates of one tumor sample based on estimates from a single matched normal. The method applies to any paired tumor-normal estimates from any microarray-based technology, combined with any preprocessing method. We demonstrate that it increases the signal-to-noise ratio of allelic signals, making it significantly easier to detect allelic imbalances.

**Conclusions:**

TumorBoost increases the power to detect somatic copy-number events (including copy-neutral LOH) in the tumor from allelic signals of Affymetrix or Illumina origin. We also conclude that high-precision allelic estimates can be obtained from a single pair of tumor-normal hybridizations, if TumorBoost is combined with single-array preprocessing methods such as (allele-specific) CRMA v2 for Affymetrix or BeadStudio's (proprietary) XY-normalization method for Illumina. A bounded-memory implementation is available in the open-source and cross-platform R package *aroma.cn*, which is part of the Aroma Project (http://www.aroma-project.org/).

## Background

The development of microarray technologies to assess DNA copy number (CN) changes was triggered by the fact that genomic alterations are hallmarks of gene deregulation and genome instability in cancers [1,2]. Among these technologies, genotyping microarrays [3-5] quantify not only total copy numbers (TCNs) but also contributions of each allele to TCN. Besides providing additional evidence for changes in TCN, allelic signal estimates can help pinpoint regions of allelic imbalance (AI) that cannot be identified from TCN alone, such as regions of copy-neutral loss of heterozygosity (LOH), or regions that are hard to detect from TCN.

In this paper we present the TumorBoost method for normalizing raw allelic signals of a tumor tissue given raw allelic signals of a matched normal tissue or blood extract. By "raw" we mean after preprocessing, but before detection of copy number events.

**Figure 1:**
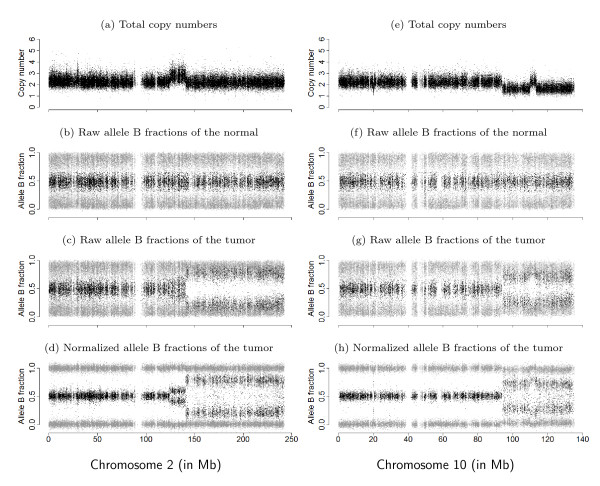
**Genomic signals from genotyping microarrays in two chromosomal regions**. Total (relative) copy numbers (a, e) and allele B fractions for the normal (b, f), the tumor (c, g) and the normalized tumor (d, h) for all SNPs on chromosome 2 (left) and chromosome 10 (right) in sample TCGA-23-1027. Homozygous SNPs (SNPs genotyped as AA or BB) are in gray, and heterozygous SNPs (AB) in black. Data are from the Affymetrix platform.

The result of applying TumorBoost is shown in Figure 1 for two chromosomes in the same individual from a pair of matched tumor-normal samples. The top row displays observed TCNs (*C*), that is, ratios of total (summarized) signal intensities in the tumor relative to the normal. The other three rows display allelic signals as *allele B fractions *(*β*), that is, the proportion of total signal that comes from allele B. This quantity has been used in cancer studies to detect AI [5-7]. Both *C *and *β *are formally defined in Section 'Observed summarized allele-specific signals'. The second and third rows display raw values of *β *for the normal and the tumor, while the last row displays TumorBoost-normalized values of *β *for the tumor. The copy number changes observed in this figure will be interpreted in terms of normal, gained, deleted, and copy neutral LOH regions in Section 'Notation and motivation' and in Section 'Change points and regions of interest'. Already from a visual comparison it is clear that the signal-to-noise ratio (SNR) is greatly improved after normalization, which makes it easier to identify and locate CN events using existing detection methods [7-13].

The outline of this paper is as follows. In Section 'Methods', after providing the necessary notation, we describe the TumorBoost method and its algorithm and implementation. We then describe the data sets used and propose methods to evaluate the performance of TumorBoost. In Section 'Results' we formally demonstrate that CN events are detected with a greater sensitivity and specificity when allele B fractions are normalized using TumorBoost. At the end, in Section 'Discussion', we mention a dual interpretation of our method, connect it to related works, discuss possible extensions, and give future research directions, before concluding the study in Section 'Conclusions'. The acronyms used in the paper are summarized in Section 'List of abbreviations'.

## Methods

### Notation and motivation

#### Parental and total copy numbers

We define the (true) *parental copy number *(PCN) as the number of copies of each of the two parental chromosomes (without specifying the phase). Formally, we denote the unphased PCN, or PCN for short, for sample *i *∈ {1, ..., *I*} at locus *j *∈ {1, ..., *J*} by (*C*_*ij*1_, *C*_*ij*2_), with *C*_*ij*1 _≤ *C*_*ij*2_. *C*_*ij*1 _and *C*_*ij*2 _are called minor and major CNs, respectively. The true TCN is then *C*_*ij*1 _+ *C*_*ij*2_.

In a region with no chromosomal aberrations, the true PCN is constant (the same for all loci). Consider a non-contaminated homogeneous tumor. In a chromosomal region that is diploid ("normal"), the true PCN is (1, 1); it is (0, 1) for a hemizygous deletion, (0, 0) for a homozygous deletion, (1, 2) for a single gain, and (0, 2) for a copy-neutral LOH.

We wish to emphasize that this paper is not about estimating the true PCN levels or proposing a new copy number change point detection method, but about improving preprocessing in order to enhance the power to detect *changes *in underlying PCN states using existing change point detection methodologies.

#### Observed summarized allele-specific signals

In practice we do not observe regions but individual probes, and for these probes we cannot observe PCNs but only total or allele-specific signals. Specifically, genotyping microarrays target a large number of single nucleotide polymorphisms (SNPs), currently of the order of 10^6 ^for the latest generation of the two most popular assays, Affymetrix's oligo-nucleotide arrays and Illumina's bead arrays.

##### Allele-specific SNP signals

We assume that normalization and preprocessing of probe-level data have already been done using one of several existing methods [5,14-19]. For hybridization *i *and SNP *j*, we then have summarized *allele-specific SNP intensity estimates *(*θ*_*ijA*_, *θ*_*ijB*_).

##### Total intensities and allele B fractions

A convenient alternative representation of these signals is (*θ*_*ij*_, *β*_*ij*_), with(1)

where *θ*_*ij *_is referred to as the *total (non-polymorphic) SNP signal *[16] and *β*_*ij *_as the *allele B fraction*. The latter is also known as the allele B *frequency *(AF or BAF) [5,7] (which should not be mistaken for a frequency in a population) and as the allele ratio (AR) [6]. A closely related quantity is the relative allele score (RAS) [20]. In [9], the authors defined the raw allele A proportion (RAP), which is 1 - *β*. The above transform from (*θ*_*ijA*_, *θ*_*ijB*_) to (*θ*_*ij*_, *β*_*ij*_) is bijective, except for the rare case when *θ*_*ij *_= 0.

It is interesting to note that the total SNP signals {*θ*_*ij*_} in Equation (1) are comparable to the signals obtained by non-polymorphic markers, which exist on the recent Affymetrix and Illumina arrays, and for which allele B fractions are not defined.

##### Total and allele-specific copy numbers

As Affymetrix and Illumina are single-sample technologies and there exist large locus-to-locus variation in total SNP signals, it is not feasible to infer CNs from a single hybridization. Therefore, the *observed *TCN *C*_*ij *_for sample *i *and SNP *j *is calculated relative to the total SNP signal of a reference *R*, as(2)

where *θ*_*Rj *_is the total SNP signal in reference *R*, with the *true *TCN assumed to be two for a diploid SNP.

Allele-specific copy numbers (ASCNs), (*C*_*ijA*_, *C*_*ijB*_), can be calculated analogously:(3)

while the observed TCN is *C*_*ij *_= *C*_*ijA *_+ *C*_*ijB*_.

#### Paired tumor-normal designs

In this paper, we focus on the experimental design where both a normal and a tumor sample are available for a given individual. From the corresponding two hybridizations, we obtain the total copy number in the tumor relative to the normal (*C*_*Tj*_), and allele B fractions for the tumor (*β*_*Tj*_) and the normal (*β*_*Nj*_) as(4)

In a region of no copy number alteration, the true TCN is 2 and the true allele B fraction, denoted by *μ*, is either 0, 1/2 or 1, where 0 and 1 correspond to homozygous genotypes AA and BB, and 1/2 corresponds to the heterozygous genotype AB. This is why allele B fractions in a normal sample (*β*_*N*_) are expected to have three bands, as observed in the second row of Figure 1.

Because of copy-number changes, the true allele B fractions (*μ*_*T*_) of a tumor can take different values in [0, 1], not only {0, 1/2, 1}. We next illustrate this point by giving an interpretation of the chromosome 2 regions observed in Figure 1. The region from 0 to 124 Mb on chromosome 2 is normal, that is, the true PCN is (1, 1) yielding that the true TCN is 2 and true allele B fractions are in {0, 1/2, 1}. The region from 124 Mb to 141 Mb has gained one or more copies. Assuming a single-copy gain, so that the true PCN is (1, 2), then the true TCN is 3 and there exist four possible ASCN states corresponding to the two possible allocations of each parental allele: {AAA, AAB, ABB, BBB}. The corresponding true allele B fractions are therefore {0, 1/3, 2/3, 1}. The region from 141 Mb to the end of chromosome 2 is a region of copy neutral LOH, where the true PCN is (0, 2) yielding true TCN is 2 and true allele B fractions in {0,1}.

Importantly, because of *normal contamination*, that is, the presence of normal cells in what is called the "tumor sample", we observe an unknown mixture of CN signals from tumor and normal cells, which reveals itself both in the observed TCNs and the observed allele B fractions. As further described in Section 'Normal contamination and its impacts', as well as in [5-7], this explains why we observe four bands for *β*_*T *_instead of two in a region of LOH. Among other things, normal contamination also explains why the mean levels of the observed bands deviate from the expected levels of a pure tumor. See also [21-25] for discussions on normal contamination.

##### Distinction between genotypes and ASCNs

In this paper we will use the terms *genotype*, *homozygous *and *heterozygous *only when referring to SNPs in a *normal *sample. In a tumor sample, which may be contaminated by normal cells or other types of tumor cells, the true allele B fractions (*μ*_*T*_) are not necessarily in {0, 1/2, 1}. Because of this, we instead use the term *true allele-specific copy number *(ASCN) for tumors (and not the term genotype).

#### A systematic genotype-specific SNP effect

**Figure 2:**
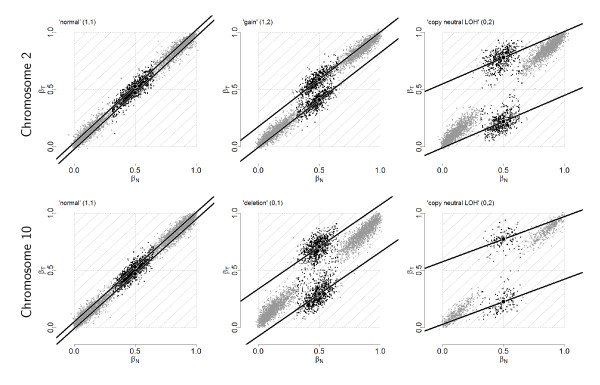
**Raw paired allele B fractions**. Paired observed allele B fractions, (*β*_*N*_, *β*_*T*_), of tumor TCGA-23-1027 versus its matched normal in six regions of constant PCN for the tumor. **Top panels**: normal (left), gained (middle), and copy-neutral LOH (right) regions from chromosome 2. **Bottom panels**: normal (left), deleted (middle), and copy-neutral LOH (right) regions from chromosome 10. SNPs called homozygous (AA and BB) are in gray. Linear models were robustly fitted to the heterozygous SNPs above and below the diagonal (black lines). Black discs mark the center of each cloud.

From Figure 1, there is considerable variation along the genome in both *β*_*T *_and *β*_*N*_, even for a normal sample where the true allele B fractions are either 0, 1/2 or 1. Figure 2 provides another representation of this data, showing scatter plots of raw allele B fractions in the tumor against the normal sample. Each point corresponds to a SNP, and each panel corresponds to a region with constant CN level (no change points) in Figure 1. SNPs called homozygous ( ∈ {0, 1}) are in gray.

The fact that the observed values differ from the true ones - 0, 1/2 or 1 in a region of no copy number alteration in the tumor (left panels) - is an indication of a *SNP effect δ*, which is not taken care of by preprocessing. We formally define this SNP effect for SNP *j *in sample *i *as the deviation(5)

between the observed and true allele B fractions. The fact that the observed values extend along the diagonal provides evidence that this SNP effect is *reproducible between the normal and the tumor*, that is, *δ*_*Tj *_and *δ*_*Nj *_are positively correlated.

In a region of copy number alteration in the tumor (middle and right panels), the true allele B fractions are still 0 and 1 for homozygous SNPs, but they deviate from 1/2 for heterozygous SNPs, as explained above. For example, in a region where one copy has been gained (top middle panel), a SNP which is AB in the normal will be AAB or ABB in the tumor, so that the true allele B fractions are either 1/3 or 2/3 for heterozygous SNPs in absence of contamination by normal cells. This explains why we are observing two clouds of heterozygous SNPs instead of one. However, due to the presence of normal cells in the tumor sample, the observed clouds of points are shrunk toward 1/2, which is the true allele B fraction in the normal.

### Normalization of allele B fractions

Figure 2 illustrates a strong, genotype-specific, positive correlation between *β*_*T *_and *β*_*N*_. The essence of TumorBoost normalization is to take advantage of the normal sample, for which true allele B fractions (genotypes) are not difficult to infer, in order to estimate SNP effects and remove them from the tumor, *without having to detect or control for CN changes in the tumor*.

For any given SNP *j*, we can estimate the SNP effect (5) in the normal sample as(6)

where  is the estimated normal genotype of SNP *j*, that is, either 0, 1/2 or 1. Genotype calling is discussed in Section 'Calling normal genotypes'. We define *TumorBoost-normalized allele B fractions in the tumor *as(7)

where  is an estimate of the SNP effect in the tumor sample, depending on the (normal) genotype, as follows.

**For homozygous SNPs **( ∈ {0, 1}), the observed allele B fractions extend along the diagonal *β*_*T *_= *β*_*N*_, regardless of the underlying CN state. Therefore, a natural estimate of the SNP effect in the tumor is , which yields the following expression for normalized allele B fractions:(8)

**For heterozygous SNPs **( = 1/2), estimating *δ*_*Tj *_as  would lead to overcorrecting allele B fractions. Indeed, the slopes of the regression lines for the heterozygous clusters in Figure 2 are less than one in regions of copy number alteration, as illustrated by the comparison with the gray diagonal lines. Therefore, we estimate the SNP effect in the tumor as , where 0 ≤ *η*_*j*_*≤ *1 is a scale factor set to *β*_*Tj*_/*β*_*Nj *_if *β*_*Tj *_≤ *β*_*Nj*_, and symmetrically as (1 - *β*_*Tj*_)/(1 - *β*_*Nj*_) if *β*_*Tj *_>*β*_*Nj*_. As a result, we normalize allele B fractions for *heterozygous SNPs *as(9)

Note that normalizing the normal sample using these equations, if done, would lead to , which means that the SNP effect would be removed completely from the normal. Note also that the TumorBoost method (Equations (8) & (9)) is applied, not only to each tumor-normal pair independently, but to each SNP independently, which also explains the choice of *η*_*j*_. In particular, it does not require prior knowledge about CN change points and CN regions.

### Total CN signals are not normalized

By design, the proposed method does *not *adjust non-polymorphic signals ({*θ*_*ij*_}) or total CNs ({*C*_*ij*_}), neither for SNPs nor for non-polymorphic CN loci - it corrects only for systematic effects in the tumor allele B fractions ({*β*_*Tj*_}), which by definition exist only for SNPs. This means that change point detection methods operating *solely on TCNs*, will identify the same CN regions regardless of TumorBoost. Only methods utilizing also allele B fractions that will gain from TumorBoost correction.

### Calling normal genotypes

**Figure 3:**
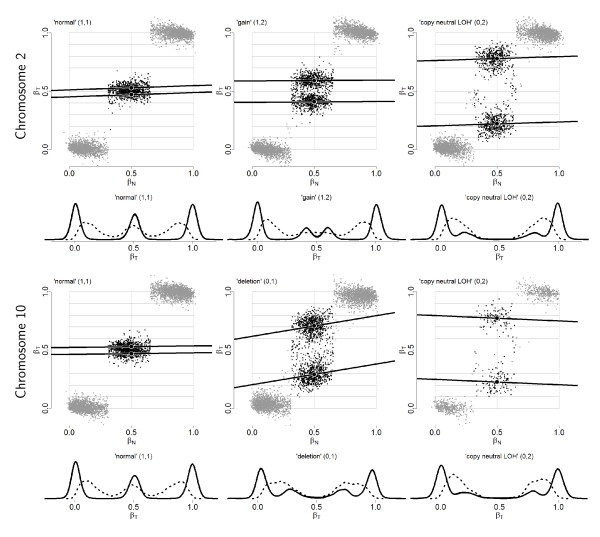
**Paired allele B fractions after TumorBoost normalization**. Paired allele B fractions, (*β*_*N*_, ), and empirical densities of the raw (*β*_*T*_; dashed) and the normalized (; solid) allele B fractions for sample TCGA-23-1027. The same regions, SNPs and annotation as in Figure 2 are used.

TumorBoost normalization relies on normal genotypes in order to call a SNP homozygous or heterozygous. Several methods [17,19,26-29] already exist that provide high-quality genotype calls. These are multi-sample (population-based) methods that leverage the accuracy of the calls by using a large pool of reference samples and/or prior parameter estimates. In case such calls are not available, but also in order to make our method applicable to a single tumor-normal pair, we introduce the following "naive" genotyping algorithm. For each autosomal chromosome in a given normal hybridization, we define genotype classes by thresholding *β*_*Nj *_at the two local minima of the empirical density of {*β*_*Nj*_}. We estimate this density using a Gaussian kernel estimator. Examples of such density distributions for diploid SNPs can be seen in the left panels of Figure 3 (dashed curves). For sex chromosomes that are not diploid, the number of genotype classes and thresholds are adjusted accordingly. All SNPs are genotyped using the same thresholds.

### A pipeline for a single tumor-normal pair

By using an allele-specific version of the single-array CRMA v2 [18] preprocessing method for Affymetrix SNP & CN arrays in combination with the TumorBoost method and the above naive genotyping algorithm, the complete pipeline is applicable to a single pair of tumor-normal hybridizations. To emphasize this, note that the Affymetrix-based signals in all figures of this paper are based on two hybridization (CEL files) only, without utilizing external references or prior parameter estimates.

Analogously to CRMA v2 for Affymetrix, the (proprietary) "XY normalization" method available in the BeadStudio software [19] can be used to process individual Illumina hybridizations.

Being able to process a single tumor-normal pair at the time, independently of others, has several implications: (i) each tumor-normal pair can be analyzed immediately without needing reference samples, (ii) lab and/or batch effects are less of a concern as long as the tumor and normal samples are processed in the same batch, (iii) samples from different individuals can be processed in parallel on different hosts/processors making it possible to decrease the processing time of large data sets, and (iv) contrary to multi-sample methods, there is no need to reprocess the samples from a given individual when new samples are produced, which further saves time and computational resources. Furthermore, (v) the decision to filter out poor quality samples can be postponed, because such samples will not affect the processing of other samples. More importantly, a method applicable to a single tumor-normal pair is (vi) more practical for applied medical diagnostics, because each patient can be analyzed at once, even when they come singly rather than in batches. This may otherwise be a limiting factor in projects with a large number of samples, or, conversely, in projects with a very small number of samples.

### Algorithm and implementation

The TumorBoost normalization method is available in the *aroma.cn *package and CRMA v2 is part of the *aroma.affymetrix *package [30], implemented in R [31] running on any operating system. Both are open source and available via the Aroma Project (http://www.aroma-project.org/), where further documentation exists. The method can be inserted as a normalization step in most preprocessing pipelines, and both low-level and high-level implementations are available. The algorithm is designed to have bounded-memory usage, regardless of the number of samples/arrays processed. Furthermore, the complexity of the algorithm is *O*(*J*), i.e. linear in the number of SNPs. The tumor-normal pairs can be normalized in parallel on multiple hosts/processors. Both TumorBoost and the naive genotyping method apply to estimates obtained by any genotyping microarray technology.

### Data sets

We used data from The Cancer Genome Atlas (TCGA) project [32,33], a collaborative initiative to better understand several types of cancers using existing large-scale whole-genome technologies. From the Data Coordinating Center (http://tcga-data.nci.nih.gov/), we downloaded (May 2009) data for a set of ovarian (serous cystadenocarcinoma, OV) and a set of brain (glioblastoma multiforme, GBM) tumor-normal pairs. For Affymetrix GenomeWideSNP_6, we downloaded the raw data (CEL files), and estimated (*θ*_*A*_, *θ*_*B*_) using an allele-specific version of CRMA v2 [18], where the probe summarization step is done for each allele separately instead for the total signal. For this platform, we also downloaded data summarized by the RMA/median-polish pipeline (ismpolish.data.txt files) to illustrate the results of using TumorBoost with other preprocessing methods (Additional Files 1 and 2). To assess the impact of naive genotyping on TumorBoost normalization, we downloaded the Birdseed [17] genotype calls.

For Illumina, we downloaded the XY-normalized and summarized data (XandYintensity.txt files) containing (*θ*_*A*_, *θ*_*B*_) as calculated by the Illumina BeadStudio software [19,34]. In addition, in order to compare the results from using truly single-sample ASCN estimates with those using optimized population-based estimates [5] (also implemented in BeadStudio), we downloaded the allele B frequency data (BAF.txt files) for the ovarian data set.

### Evaluation methods

#### Change points and regions of interest

We have evaluated the TumorBoost method on a large number of the downloaded samples as well as on other data sets (not shown). However, for the purpose of illustrating our method, we will focus on chromosomes 2 and 10 of OV sample TCGA-23-1027 based on Affymetrix GenomeWideSNP_6 data (Figure 1). Results based on Illumina Human1M-Duo data and other preprocessing methods are available in the Additional Files 3, 4, 5, 6, 7, 8.

For completeness, in order to illustrate the performance of TumorBoost on a different tumor type and different platform, the results of an evaluation of TumorBoost sample TCGA-02-0001 are given in Additional Files 9, 10, 11, 12. This is a GBM tumor which has been assayed by Affymetrix GenomeWideSNP_6 and Illumina HumanHap550.

The evaluation is done at four different allele B fraction change points corresponding to four common PCN-state transitions, and at one region with no change point (negative control) as summarized in Table 1. It is worth noting that the general conclusions of this study will be the same whether the true underlying PCN states are known or not. However, in order to simplify the discussion we choose to label the states and propose the following biological interpretation.

**Table 1:** Change points and genomic regions studied.

Label	Chr.	Region	Change point	Safety region	PCN_1_	PCN_2_
N/G	2	108.0-140.0	124.0	1.00	normal (1,1)	gain (1,2)

G/L	2	125.0-157.0	141.0	1.00	gain (1,2)	copy-neutral LOH (0,2)

N/D	10	80.0-109.0	94.0	1.00	normal (1,1)	deletion (0,1)

D/L	10	106.5-113.5	110.0	1.00	deletion (0,1)	copy-neutral LOH (0,2)

N/N	2	55.0-75.0	60.0	1.00	normal (1,1)	normal (1,1)

Each change point, covered by a 1.00 Mb safety region, separates two unique PCN regions (PCN_1 _and PCN_2_). The change points on the same chromosome share parts of the same PCN region. All lengths are in units of mega base pairs (Mb).

For chromosome 2, we believe that the tumor has (i) gained a region from 124 Mb to the end of the chromosome, and (ii) lost the other parental chromosome from 141 Mb to the end of the chromosome. As a result, we observe a region with two PCN change points: N/G between the (1, 1) normal region and the (1, 2) gain, and G/L between the (1, 2) gain and the (0, 2) copy-neutral LOH region. For chromosome 10, a possible scenario is that the tumor has (i) lost a region from 94 Mb to the end of the chromosome, and then (ii) gained the other parental chromosome from 110 Mb to 114 Mb. As a result, we observe a chromosomal region with two PCN change points: N/D between the (1, 1) normal region and the (0, 1) deletion, and D/L between the (0, 1) deletion and the (0, 2) copy-neutral LOH region.

#### Detecting CN events from allelic signals

##### Mirrored allele B fractions

Detecting PCN changes using {*β*_*ij*_} is not straightforward because its distribution has up to four modes in a given ASCN region (Figure 1 and Figure 3). However, as the distribution of {*β*_*ij*_} is expected to be symmetric around the heterozygous band, it is convenient to work with mirrored allele B fractions: |*β*_*ij *_- 1/2| as defined in [7]. Note that this quantity is also related to the folded BAF in [24] and to the observed major copy proportion (MCP) defined by [9]. The TumorBoost-normalized version of this quantity is given by ||.

##### Decrease in Heterozygosity

Although TumorBoost corrects allele B fractions for all SNPs, it is typically only heterozygous SNPs that are used for downstream PCN analyses, as they carry all the information regarding AI [5,7,10,24]. We define the *Decrease in Heterozygosity *(DH) in the tumor sample for a heterozygous SNP *j *as(10)

DH is close to zero when there is balance between the alleles and parental chromosomes (*C*_1 _= *C*_2_), and deviates from zero when there is AI, e.g. DH is close to one in a region of LOH if it is a pure tumor. The TumorBoost-normalized version of DH is defined (for a heterozygous SNP *j*) by(11)

by Equation (9). Note that in this setup the essence of TumorBoost lies in the difference |*β*_*Tj *_- *β*_*Nj*_| with a correction factor. In [5], the authors briefly mention (in the caption of Figure 7) that "the allele frequency *difference *between normal and tumor genotypes is very distinct", unfortunately without further discussion. Independently, we have found that a normalization that leaves out this correction factor also improves the power to detect PCN change points with respect to raw DH, but is suboptimal because it overcorrects allele B fractions for heterozygous SNPs, as explained in Section 'Normalization of allele B fractions' above. In a region of constant PCN, the distribution of decrease-in-heterozygosity signals (DHs) has at most two modes, and at most one if only heterozygous SNPs are considered. The latter property makes it possible to use existing segmentation methods originally proposed for total CN analysis [35-37] to detect PCN changes [5,7,10,24].

Inspired by how these segmentation methods work, we propose an evaluation framework that quantifies the power to detect a PCN change point from {*ρ*_*Tj*_} and {}. Analogously to [18,38], we assume that we know the location of a PCN change point with high precision and that there exist no other change points nearby. To protect ourselves against errors in the location, we add a safety margin on each side such that the true location is within the safety region with high confidence (see also Table 1). Furthermore, we assume that the change point, together with the safety region, separates two flanking regions with constant parental copy numbers PCN_1 _and PCN_2_. We know that the true decrease in heterozygosity differs between regions but not within regions. Differences observed within a region are assumed to be due to random errors. By comparing the DHs for heterozygous SNPs in the two regions, we can assess if they differ and if so, by how much.

##### Testing for equal mean levels

The simplest way to test whether {*ρ*_*Tj*_} for the two regions originates from the same class of PCNs or not is to use Student's *t*-test to test if the means of the heterozygous SNPs are equal. This is also the most common test used by existing segmentation methods. We calculate the *t *statistic for each change point in Table 1, where a false change point (N/N) has been added as a negative control. We also calculate the *t *statistic based on the total CNs for the same heterozygous SNPs as are used for the allele B fractions. Non-polymorphic loci are not considered as they are not affected by the TumorBoost method.

As the *t *statistic depends on sample size, and as different regions have different sizes, the test statistics may not be comparable across regions and methods. We therefore sampled a fixed number of heterozygous SNPs for each method and each region (*J' *= 250). Because the observed *t *statistic depends on the sampled data points, we use bootstrap techniques (resampling *B *= 100 times) to estimate the mean and standard deviation of each test statistic.

##### ROC curve analysis

An alternative is to assess how well the two regions on each side of the change point *separate*. For each change point, heterozygous data points (excluding those in the safety region) are annotated as belonging to either of the two CN states. In order to control for sample-size effects, we balance the number of true positives and true negatives, by sampling so that both regions have the same number of data points. In order to control for the fact that different genotyping methods yield different numbers of SNPs called heterozygous, we also constrain the number of data points sampled to be the same when comparing results involving different genotyping methods. Next, we use receiver operating characteristic (ROC) analysis to assess how well raw and normalized DHs discriminate the two states studied. This evaluation is done on full-resolution (*H *= 1) as well as smoothed signals, where DHs are averaged (non-robust) in non-overlapping bins of *H *= 2 and *H *= 4 data points per bin. We will return to the smoothed CNs in Section 'Influence of genotype calls on normalization' when discussing sensitivity to genotyping errors. For each comparison, we define the "positive" state as the state with TCN different from two. A similar approach was used in [18,38] for assessing total CN separation.

#### Robustness against genotyping errors

As genotypes are used for TumorBoost normalization, the performance of our method depends on genotype quality. To assess TumorBoost's sensitivity to errors in genotype calls, we also use genotype calls from population-based methods: Birdseed [17] for Affymetrix data, and BeadStudio [19] for Illumina data. Like most available methods for detecting CN changes using DH, our *evaluation *itself focuses on heterozygous SNPs, which makes it depend on the genotyping algorithm. For consistency, TumorBoost-normalized DHs are evaluated based on the same genotyping method as was used for normalization. The evaluation of raw DHs is done using the best genotyping method. Genotyping errors are discussed further in Section 'Influence of genotype calls on normalization' and Section 'Influence of genotyping errors'.

#### Normal contamination and its impacts

As with many tumor samples, tumor TCGA-23-1027 is also contaminated with normal (and possibly also other) cells. As a result, we do not observe only two but four homozygous allele B fraction bands in LOH regions (Figures 1 &2).

For simplicity, assume that the tumor sample contains one type of tumor cells contaminated with normal cells so that the proportion of tumor cells is *κ *∈ [0, 1] ("tumor purity") and the proportion of normal cells is 1 - *κ *("normal contamination"). We also assume that the average tumor ploidy is two (see Section 'Directions for future research' for a discussion on this point). Then, in a tumor region where the true PCN is given by (*C*_1_, *C*_2_), the true decrease in heterozygosity for heterozygous SNPs [7] is(12)

**Figure 4:**
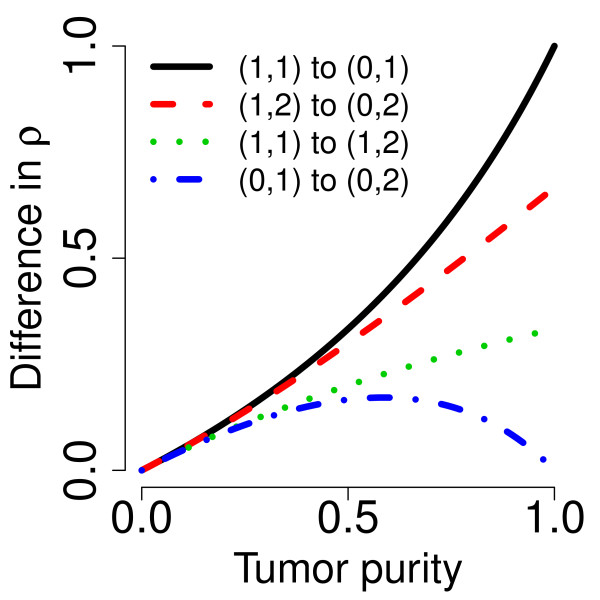
**Differences in (true) decrease in heterozygosity**. Differences in (true) decrease in heterozygosity (for heterozygous SNPs) between different pairs of flanking PCN regions as a function of tumor purity (*κ*).

If we assume that the variance of DH is independent of its mean level, then *the power to detect a change point in DHs*, using a *t *statistic, is a linear function of the absolute change in its true value,(13)

which is a function of tumor purity (*κ*), parametrized by the true PCNs (PCN_1 _and PCN_2_) of the two flanking regions. In Figure 4, this difference is plotted as a function of tumor purity for each of the four change points in Table 1. Interestingly, although it is in most cases easier to detect a PCN event the more pure the tumor is, this is not the case when the remaining parental chromosome in a deleted region is duplicated (change point D/L). In that case, the difference is greatest at *κ *= 0.59 (= 2 - ) and decreases to zero toward *κ *= 1 and *κ *= 0. Note that Equations (12)-(13) hold provided that there are no additional biases in the allele B fractions. However, because of incomplete offset correction [6,39], differences in platforms [38], and differences in preprocessing methods, the mean levels of the allele B fractions are almost certainly biased, even after normalization. Thus, we only claim that TumorBoost removes systematic effects across SNPs but we do not claim to control for the mean levels. This is why we use the term "normalization" rather than "calibration" [40]. However, as we will see later, although there may still be a global bias in the allele B fractions, the relative ordering suggested by Equations (12)-(13) is still preserved. We also want to emphasize that this paper is neither about estimating the true PCN levels nor about estimating tumor purity. The main objective is to improve the signal-to-noise ratios such that change points are better *detected*.

## Results

### Improvements from applying TumorBoost

Figure 3 displays plots of *β*_*N *_versus TumorBoost-normalized *β*_*T*_. From a direct comparison with the corresponding raw estimates (Figure 2), it is clear that *β*_*T *_and *β*_*N *_are much less correlated after normalization (when stratified on genotype). This implies that most of the SNP effects have been removed: the regression lines are close to horizontal after normalization. This in turn results in greater SNRs, because the modes of allele B fractions are sharper and more distinct after TumorBoost normalization, as seen from the density curves in Figure 3.

The improvement in SNR is also illustrated by the comparison between allele B fractions before and after normalization along chromosomes 2 and 10 in Figure 1 (bottom two rows). However, we note in this Figure that TumorBoost does introduce a few outliers in regions of decreased heterozygosity in the tumor: after 140 Mb in chromosome 2 and after 95 Mb in chromosome 10. These outliers are due to genotyping errors. They are discussed in detail in Section 'Influence of genotype calls on normalization', where we show that they are of second order when compared to the gain achieved by TumorBoost, and in Section 'Influence of genotype calls on normalization', where we demonstrate how they can be avoided by existing downstream change-point detection methods.

**Figure 5:**
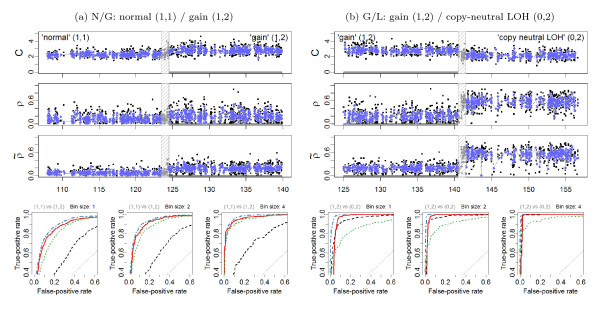
**ROC evaluation (Chr 2)**. **(a) Left panels: **The region 108.0-140.0 Mb on Chr 2 in tumor-normal sample TCGA-23-1027 has a change point at approximately 124.0 Mb, which separates a normal diploid state from a gain. 1,171 loci in each of these two states are used for the evaluation. All 79 loci in the safety region have been excluded. **(b) Right panels: **The region 125.0-157.0 Mb on Chr 2 in tumor-normal sample TCGA-23-1027 has a change point at approximately 141.0 Mb, which separates a normal diploid state from a gain. 986 loci in each of these two states are used for the evaluation. All 64 loci in the safety region have been excluded. The top three rows show the total CNs (*C*), and the raw (*ρ*) and normalized () heterozygous DHs, respectively. A 1000 kb safety region (dashed gray frame) around the change point is excluded from the evaluation. The full resolution data points are colored black and the binned (*H *= 4) ones are colored blue. The three panels in the bottom row show the ROC performance of the TCNs (dotted green) and the raw (dashed black) and normalized (solid red and dot-dashed blue for naive and population-based genotypes, respectively) DHs at the full resolution (*H *= 1; no binning), and after binning in non-overlapping windows of size *H *= 2 and *H *= 4 SNPs, respectively.

**Figure 6:**
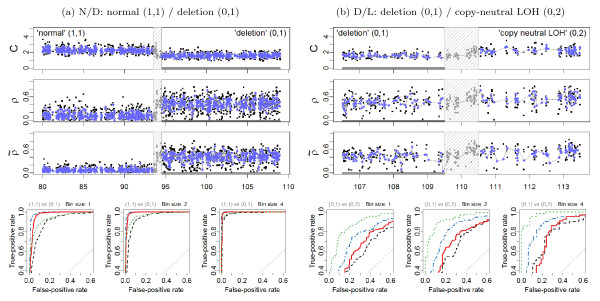
**ROC evaluation (Chr 10)**. **(a) Left panels: **The region 80.0-109.0 Mb on Chr 10 in tumor-normal sample TCGA-23-1027 has a change point at approximately 94.0 Mb, which separates a normal diploid state from a deletion. 1,276 loci in each of these two states are used for the evaluation. All 53 loci in the safety region have been excluded. **(b) Right panels: **The region 106.5-113.5 Mb on Chr 10 in tumor-normal sample TCGA-23-1027 has a change point at approximately 110.0 Mb, which separates a copy-neutral LOH region from a deletion. 254 loci in each of these two states are used for the evaluation. All 59 loci in the safety region have been excluded. The outline is the same as in Figure 5.

Because the SNR increases for each PCN region, it is possible to argue that the SNR for *the difference between DHs in regions flanking a change point *also increases making it easier to detect this change point. Figure 5 and Figure 6, in which DHs before and after normalization are plotted for each change point investigated, confirm that this is the case, at least for the change points N/G, G/L and N/D. To quantify this, we applied a *t*-test for each change point with the null hypothesis that the mean DH levels are equal in the two flanking regions, as described in Section 'Detecting CN events from allelic signals'. The *t *statistics in Table 2 demonstrate that TumorBoost normalization greatly improves the power to detect PCN events using DHs. The test statistics are larger after normalization than before, both when naive and Birdseed genotype calls are used. We also find that the changes are within the error limits for the negative control. These conclusions also hold for data from the Affymetrix platform summarized using the RMA/median-polish pipeline, and for data from the Illumina Human1M-Duo platform (Additional Files 1, 2, 3, 4, 5, 6, 7, 8, 9, 10, 11, 12: Supplemental Table S2).

**Table 2:** Mean and standard deviation of the (absolute) Student's t statistics to test the null hypothesis of equal means in two flanking PCN regions based on heterozygous SNPs only.

Heterozygous SNPs only						
**Signals**	**Genotypes**	**N/G**	**G/L**	**N/D**	**D/L**	**N/N**
Raw DH (*ρ*_*T*_)	Birdseed	7.17 ± 1.13	27.79 ± 1.93	24.22 ± 1.35	8.88 ± 1.04	0.78 ± 0.64
Normalized DH ()	Birdseed	21.00 ± 1.45	39.42 ± 2.54	40.22 ± 2.36	11.04 ± 1.13	0.74 ± 0.59
Normalized DH ()	naive	18.61 ± 1.39	33.38 ± 2.31	35.40 ± 2.07	9.40 ± 1.01	1.06 ± 0.68
TCN (C_*T*_)	Birdseed	16.43 ± 1.05	18.55 ± 1.26	25.69 ± 1.32	20.57 ± 1.23	2.19 ± 0.99

Greater mean values correspond to greater power to detect a change point. Raw (top line) and TumorBoost-normalized (second and third line) DHs, and TCNs (bottom line).

These findings are further confirmed by the ROC analyses of the four change points at the full and the smoothed resolutions, as summarized by the ROC curves in Figure 5 and Figure 6. Specific points raised by these results are addressed in the following sections.

### Influence of genotype calls on normalization

In general, the influence of the genotyping method is of second order: the results obtained when using naive genotyping are almost as good as when using more elaborate (population-based) genotype calls. This result holds regardless of the normalization and summarization method (see Additional Files 1, 2, 3, 4, 5, 6, 7, 8, 9, 10, 11, 12). For Affymetrix data preprocessed using CRMA v2, this can be seen by comparing the corresponding ROC curves in Figure 5 and Figure 6 and by comparing the rows of Table 2. However, we note that for the G/L change point, normalization based on the former does worse than not normalizing if the false-positive (FP) rate *of an average SNP *is required to be small enough, i.e. here FP rate ∈ [0, 0.05]. The reason for this is that there exists a set of false positives ("outliers") that are due to *genotyping errors *- truly homozygous SNPs are incorrectly identified as heterozygous by the naive genotyping method. These outliers can be spotted in the non-normalized DH track in Figure 5.

However, as these false positives are *individual SNP *scattered along the genome, we argue that this is not a serious problem for downstream copy number change point detection methods (in particular segmentation methods), because their objectives are not to call *individual SNPs*, but to infer *segments of several consecutive SNPs *with the same PCN. Furthermore, segmentation methods usually require more than one data point in order to calculate the test statistic. When allowing for two or more data points per CN region, the false-positive rate goes down substantially while remaining a significantly improved true-positive rates. This is confirmed by the ROC analysis of (*H *= 2, 4) smoothed signals (Figure 5 and Figure 6), and the *t *statistics (Table 2).

In Section 'Influence of genotyping errors' we discuss further options for decreasing the number of genotyping errors, and suggest how segmentation methods can be made more robust against them.

### Power to detect PCN change points

The above comparison of the results obtained using normalized DHs across change points implies an ordering in the power to detect these change points, which can be seen from ranking either the test statistics in Table 2 or the ROC curves in Figure 5 and Figure 6. These results suggest that it is much easier to detect a change point between a gain and copy-neutral LOH region (G/L) or between a loss and a normal region (N/D), than it is to detect a change point between a gain and a normal region (N/G). The hardest change point to detect is the one between a deletion and a copy-neutral LOH region (D/L). Note that this is consistent with the differences in true DHs (Equations (12)-(13)), which are depicted in Figure 4. We expect to have little power to detect a D/L change point using DH.

From this it also follows that, although the power to detect either of the two change points of a gain, or a loss, surrounded by a copy-neutral region is the same for total CNs, this is not the case for DHs. A consequence of this is that, even with a detection method that takes advantage of both TCN and DH, we are more likely to detect certain types of change points before others, and the precision in locating them will also differ.

Note that the evaluation presented is not designed to compare the power of TCN and DH to detect PCN change points, as we are comparing the average detection power of *heterozygous SNPs *only. In order to perform such a comparison, we would also need to take into account homozygous SNPs and non-polymorphic loci for TCNs, and compare ROC curves at a resolution defined by bin widths and not bin counts.

### Other platforms and preprocessing methods

The aforementioned results are all based on Affymetrix GenomewideSNP_6 data that was preprocessed by the CRMA v2 method. In order to show that the results hold for other preprocessing methods and microarray genotyping platforms, we applied TumorBoost to the same Affymetrix data set after RMA/median-polish preprocessing (by Birdseed), as well as to the Illumina data sets preprocessed using BeadStudio. In all cases the conclusion is that TumorBoost improves the SNRs and the power to detect change points, and that the relative power of different types of change points is consistent with the ones expected by theory. It is interesting to notice that the allele B fractions obtained by the RMA/median-polish method are attenuated, and that TumorBoost also corrects for this. As discussed further in Section 'Discussion', this compression is due to incomplete offset and crosstalk correction. For the Illumina data set, we only report the results obtained with naive genotype calls. We did not perform a comparison with the results obtained with BeadStudio genotype calls as a substantial proportion of SNPs (4%) were not called by BeadStudio, making the results of the comparison depend on the (unknown) reason why these SNPs were not called by BeadStudio. However we note that naive genotype calls already perform near perfectly for this data set.

## Discussion

### Influence of genotyping errors

Above we have noted that although our normalization method leads to an improved signal ratio at the chromosome or at the genome scale, SNPs that have been incorrectly called heterozygous will still appear as outliers after TumorBoost normalization. We have argued that this is not a major problem for downstream analysis methods. In this section we show how genotyping errors by our naive genotyping algorithm can be avoided, and suggest ways to make segmentation methods robust against them.

By construction of our naive genotype calling algorithm, genotyping errors correspond to SNPs for which the allele B fraction is close to the estimated minimum of the density. Therefore, some of these errors can be avoided by making more conservative heterozygous calls in the first place. Our results show that if we remove the 10% SNPs with lowest confidence scores for each method compared, the power *per SNP *obtained by TumorBoost using naive genotype calls increases and becomes comparable to that achieved by more elaborated population-based genotyping algorithms (ROC curves in Additional Files 2, 4, 6, 8, 10, 12).

Importantly, we observe a gain after taking confidence scores into account for TumorBoost-normalized data with naive genotype calls even after adjusting for the loss in resolution due to the discarding of 10% of the data points. This can be seen from the comparison between *t *statistics *across *choices of genotype confidence-score thresholds, where we adjusted the number of heterozygous SNPs accordingly. For example, when restricting to the 90% best genotype calls, we used *J' *× 90% = 225 points (Additional Files 1, 2, 3, 4, 5, 6, 7, 8, 9, 10, 11, 12: Supplemental Table S2).

Furthermore, a two-dimensional genotyping algorithm that takes advantage of the fact that the genotype clusters are better separated in the (*β*_*N*_, *β*_*T*_) space (Figure 2) is likely to perform better than a naïve genotyping algorithm that is based on *β*_*N *_alone.

Finally, we note that it is possible to make existing segmentation methods more robust against genotyping errors when genotype confidence scores are available, such as scores from the above naive genotyping algorithm, scores provided by existing genotyping algorithms, or generic scores [41]. Confidence scores can be used to give greater weights to SNPs with better genotype calls. Recently the authors of Circular Binary Segmentation (CBS) added support for such weights to their method [37]. On top of this, one can utilize an iterative re-weighted approach where the outliers found from one iteration of segmentation are down-weighted in the following iteration until convergence.

### Interpretation in terms of allelic crosstalk

**Figure 7:**
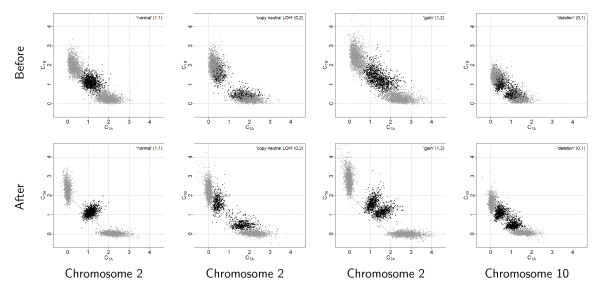
**Influence of TumorBoost normalization on allele-specific copy numbers**. Allele-specific CNs, (*C*_*TA*_, *C*_*TB*_), of tumor TCGA-23-1027 before (top panels) and after (bottom panels) TumorBoost normalization in a normal region (column 1), in a copy-neutral LOH region (column 2), in a gain (column 3), and in a deletion (column 4). These are some of the regions in Figure 2 using the same SNPs and annotations.

From Equations (1), (8) & (9) one can show that TumorBoost can also be written as(14)

where(15)

and(16)

being a scale factor controlling for the total copy number and protecting against overcorrection (dual to *η*_*j*_). From Equations (14)-(15) one see that for an AA SNP, any extra signal observed in allele B of the normal is (partly) subtracted from the allele B, and added back to allele A, of the tumor. A BB SNP is corrected analogously. For a SNP that is AB, the correction is toward the diagonal along the line *θ*_*B *_= *θ *- *θ*_*A *_such that if it would be applied to the normal, the normalized signals would be (*θ*/2, *θ*/2). This is an interpretation of as well as a dual motivation for TumorBoost - there exists a SNP-specific crosstalk between the two alleles such that one or both alleles "pull" signal from the others, while their total signal is preserved (a necessary identifiability constraint in the paired setup). The effect of TumorBoost on the ASCNs, calculated as in Equation (3), can be seen in Figure 7. In a region of diploid ASCNs, there exist a large SNP-to-SNP variation within each genotype group, a variation which is decreased after normalization. Similarly, for aberrant regions, the ASCN clusters are more distinct after normalization.

### Population-based estimation of SNP effects

The main idea behind the TumorBoost method is that there exist SNP-specific effects that to a large extent are shared by the tumor and normal samples, and that it is easy to estimate them from the normal. In the case when there exist no matched normal, or when one wishes to correct the normal itself, this approach will not work. It has been previously reported that {(*θ*_*ijA*_, *θ*_*ijB*_)}_*i*_, {(*θ*_*ij*_, *β*_*ij*_)}_*i*_, or variants of these, cluster into distinct genotype groups across samples [5,17,27,29,42,43], which suggests that the SNP effects are shared across samples. This is one of the rationales behind TumorBoost. A population-based generalization of TumorBoost is then to estimate how the observed ASCNs is as a function **f**_*j *_of the true ASCNs, and then use the inverse of this function to backtransform a new sample, i.e. . One possibility is to use an affine function for each **f**_*j *_and estimate it from a large population of normal samples for which the true ASCNs can be called (0, 2), (1, 1) or (2, 0). Major challenges are how to choose and constrain **f**_*j*_, and how to deal with batch and lab effects, small sample sizes, as well as rarely-observed genotype groups. In this context, TumorBoost can be thought of as estimating a highly constrained linear function **f**_*j *_defined in a neighborhood of the genotype center as given by the normal sample, see Equations (14)-(16). This function cannot be used to correct for data at other genotypes, which is why TumorBoost applies only to paired tumor-normal samples.

Continuing, to the best of our knowledge, Illumina's proprietary method [5], which normalizes TCNs and allele B fractions by regressing on normal training data for each SNP separately, is the only method that does this and is readily implemented and widely tested and used. Note that TumorBoost still improves upon these estimates (Additional Files 7-8), which can be because TumorBoost can also correct for systematic effects specific to, but not shared across, individuals and/or batches.

In addition to Illumina's method, variants of the above ideas have been proposed by others, such as the allele-specific mixture CN model [43] and the Birdsuite method [17]. Unfortunately, these were either only proposed in theory, specific to one technology or to older chip types, used only for calling discrete ASCNs in copy-number polymorphic regions, or perform suboptimal corrections. We are aware of a few related methods under development that try to close this gap. We look forward to these when they become available.

### Directions for future research

#### Simultaneous segmentation of TCN and DH

The results presented in this paper suggest that both TCN and DH may carry information regarding the location of a PCN change point. Therefore, analyzing these two quantities separately is sub-optimal. This motivated the development of Hidden Markov Model (HMM)-based methods that make use of both pieces information simultaneously, such as PICNIC [11], GenoCN [12] and PSCN [13].

To the best of our knowledge, such joint analyses of TCN and DH cannot be done with current segmentation methods, although multi-dimensional segmentation methods for total copy number data have been developed in different contexts: multi-platform segmentation [44], and joint segmentation from multiple biological samples [45-47]. We advocate the development of joint TCN and DH segmentation methods that would close this gap.

#### Calling PCN states

A natural step downstream of the detection of PCN changes is the calling of PCN states. Besides the true copy numbers in the tumor, two biological parameters influence the observed copy number intensity levels: normal contamination, as explained in Section 'Normal contamination and its impacts', and tumor ploidy. As the total mass of DNA - not the total number of cells - is fixed by the assay, tumor ploidy in fact acts as an unknown scale factor on observed copy number intensities [6,11].

Even in situations when tumor ploidy is assumed to be known or can be estimated, calling PCN states when *κ *is unknown and estimating *κ *when PCN states are unknown are difficult problems. In order to disentangle these two problems, a natural idea is to take advantage of the fact that although PCN states differ across PCN regions, *κ *is the same across regions for a given sample.

However, we note that Equation (12) makes the strong assumption that the tumor is homogeneous, that is, the "tumor sample" is a mixture of normal tissue and *one *tumor tissue. Another complication is that DHs after normalization need to be *calibrated*; the mean DH value in a given PCN region might be a biased estimator of the true PCN, also after TumorBoost normalization.

Furthermore, as noted in Section 'Power to detect PCN change points', with joint segmentation of TCN and DH, there is a greater risk that one of the change points flanking a constant PCN region is more likely to be detected than the other. This complicates the calling and inference of the underlying PCN states. On the other hand, understanding how this bias works can help locate such expected but "missing" change points.

We are looking forward to further scientific contributions to these problems.

#### Call for the use of matched normals

Finally, we wish to emphasize the great value of including matched normals in tumor studies. To start, with paired tumor-normal data there are great opportunities for quality control, e.g. validating sample annotations and identify poor or failed hybridizations. More importantly, it is basically only with a matched normal it is possible to tell if an event is somatic or in the germline. Moreover, as reported by others and explained here, matched normals are useful for identifying homozygous SNPs, which when excluded greatly helps identifying regions of AI in the tumor. Not to mention that with a matched normal it is easier to infer the amount of normal contamination. In addition to these, we have in this study shown that with matched normals it is possible to obtain tumor CNs with significantly higher SNRs, which further helps us identify chromosomal events. For these reasons, we strongly suggest that it becomes standard to collect normal DNA (blood or tissue) along with the tumor.

## Conclusions

TumorBoost increases the power to detect somatic copy-number events (including copy-neutral LOH) in the tumor from allelic signals of Affymetrix, Illumina and alike origins. Because each SNP is normalized separately, TumorBoost does not require prior knowledge about copy number change points or copy number regions, and its complexity is linear in the number of SNPs.

Importantly, high-precision allelic estimates can be obtained from a single pair of tumor-normal hybridizations, if TumorBoost is combined with single-array preprocessing methods such as (allele-specific) CRMA v2 for Affymetrix or BeadStudio's (proprietary) XY-normalization method for Illumina. Based on these results, we recommend the use of matched normal samples in cancer DNA copy number studies.

## List of abbreviations

AI: allelic imbalance; ASCN: allele-specific copy number; CN: copy number; DH: decrease in heterozygosity; LOH: loss of heterozygosity; PCN: parental copy number; ROC: receiver operating characteristic; SNP: single nucleotide polymorphism; SNR: signal-to-noise ratio; TCGA: The Cancer Genome Atlas; TCN: total copy number.

## Authors' contributions

HB and PN developed and iterated the method with help from TS. All authors contributed to the final method and manuscript.

## Supplementary Material

Additional file 1 **Affymetrix GenomeWideSNP_6 data after CRMAv2 preprocessing (sample TCGA-23-1027)**. Assessment of TumorBoost based on tumor/normal pair TCGA-23-1027 in the Affymetrix GenomeWideSNP_6 data set preprocessed with the CRMAv2 method. Click here for file

Additional file 2 **Affymetrix GenomeWideSNP_6 data after CRMAv2 preprocessing (sample TCGA-23-1027; with confidence scores)**. Assessment of TumorBoost based on tumor/normal pair TCGA-23-1027 in the Affymetrix GenomeWideSNP_6 data set preprocessed with the CRMAv2 method using the SNPs with 90% highest confidence scores. Click here for file

Additional file 3 **Affymetrix GenomeWideSNP_6 data after ismpolish preprocessing (sample TCGA-23-1027)**. Assessment of TumorBoost based on tumor/normal pair TCGA-23-1027 in the Affymetrix GenomeWideSNP_6 data set preprocessed with the ismpolish method. Click here for file

Additional file 4 **Affymetrix GenomeWideSNP_6 data after ismpolish preprocessing (sample TCGA-23-1027; with confidence scores)**. Assessment of TumorBoost based on tumor/normal pair TCGA-23-1027 in the Affymetrix GenomeWideSNP_6 data set preprocessed with the ismpolish method using the SNPs with 90% highest confidence scores. Click here for file

Additional file 5 **Illumina Human1M-Duo data after BeadStudio,XY preprocessing (sample TCGA-23-1027)**. Assessment of TumorBoost based on tumor/normal pair TCGA-23-1027 in the Illumina Human1M-Duo data set preprocessed with the BeadStudio,XY method. Click here for file

Additional file 6 **Illumina Human1M-Duo data after BeadStudio,XY preprocessing (sample TCGA-23-1027; with confidence scores)**. Assessment of TumorBoost based on tumor/normal pair TCGA-23-1027 in the Illumina Human1M-Duo data set preprocessed with the BeadStudio,XY method using the SNPs with 90% highest confidence scores. Click here for file

Additional file 7 **Illumina Human1M-Duo data after BeadStudio,BAF preprocessing (sample TCGA-23-1027)**. Assessment of TumorBoost based on tumor/normal pair TCGA-23-1027 in the Illumina Human1M-Duo data set preprocessed with the BeadStudio,BAF method. Click here for file

Additional file 8 **Illumina Human1M-Duo data after BeadStudio,BAF preprocessing (sample TCGA-23-1027; with confidence scores)**. Assessment of TumorBoost based on tumor/normal pair TCGA-23-1027 in the Illumina Human1M-Duo data set preprocessed with the BeadStudio,BAF method using the SNPs with 90% highest confidence scores. Click here for file

Additional file 9 **Affymetrix GenomeWideSNP_6 data after CRMAv2 preprocessing (sample TCGA-02-0001)**. Assessment of TumorBoost based on tumor/normal pair TCGA-02-0001 in the Affymetrix GenomeWideSNP_6 data set preprocessed with the CRMAv2 method. Click here for file

Additional file 10 **Affymetrix GenomeWideSNP_6 data after CRMAv2 preprocessing (sample TCGA-02-0001; with confidence scores)**. Assessment of TumorBoost based on tumor/normal pair TCGA-02-0001 in the Affymetrix GenomeWideSNP_6 data set preprocessed with the CRMAv2 method using the SNPs with 90% highest confidence scores. Click here for file

Additional file 11 **Illumina HumanHap550 data after BeadStudio,XY preprocessing (sample TCGA-02-0001)**. Assessment of TumorBoost based on tumor/normal pair TCGA-02-0001 in the Illumina HumanHap550 data set preprocessed with the BeadStudio,XY method. Click here for file

Additional file 12 **Illumina HumanHap550 data after BeadStudio,XY preprocessing (sample TCGA-02-0001; with confidence scores)**. Assessment of TumorBoost based on tumor/normal pair TCGA-02-0001 in the Illumina HumanHap550 data set preprocessed with the BeadStudio,XY method using the SNPs with 90% highest confidence scores. Click here for file
