# Supplementary material for: TumorBoost: Normalization of allele-specific tumor copy numbers from a single pair of tumor-normal genotyping microarrays
Source: BMC Bioinformatics. 2010 May 12;11:245. doi: 10.1186/1471-2105-11-245 (PMC2894037; doi:10.1186/1471-2105-11-245)
Supplement: Additional file 3 — Affymetrix GenomeWideSNP_6 data after ismpolish preprocessing (sample TCGA-23-1027). Assessment of TumorBoost based on tumor/normal pair TCGA-23-1027 in the Affymetrix GenomeWideSNP_6 data set preprocessed with the ismpolish method. [file 1471-2105-11-245-S3.PDF]

Supplementary Note: Assessment of TumorBoost based on tumor/normal pair TCGA-23-1027 in the Affymetrix GenomeWideSNP_6 data set TCGA,OV,Birdseed,ismpolish

Henrik Bengtsson, Pierre Neuvial, Terence P. Speed

March 9, 2010

Contents

1 Introduction **3**

2 Data set **3**
2.1 Preprocessing methods . . . 3
2.2 Stratification on genotype confidence scores . . . 3
2.3 List of change points . . . 3

3 Region: TCGA-23-1027:Chr2@108-140,cp=124+/-0.5,s=0/1 **4**
3.1 Decrease in Heterozygosity (DH) and total copy-number tracks . . . 4
3.2 Allele B fraction density plots . . . 5
3.3 ROC curves . . . 5
3.4 (β_N, β_T) plots . . . 6
3.5 Allele-specific copy number estimates . . . 7

4 Region: TCGA-23-1027:Chr2@125.0-157.0,cp=141.0+/-0.5,s=1/3 **8**
4.1 Decrease in Heterozygosity (DH) and total copy-number tracks . . . 8
4.2 Allele B fraction density plots . . . 9
4.3 ROC curves . . . 9
4.4 (β_N, β_T) plots . . . 10
4.5 Allele-specific copy number estimates . . . 11

5 Region: TCGA-23-1027:Chr10@80-109,cp=94+/-0.5,s=0/2 **12**
5.1 Decrease in Heterozygosity (DH) and total copy-number tracks . . . 12
5.2 Allele B fraction density plots . . . 13
5.3 ROC curves . . . 13
5.4 (β_N, β_T) plots . . . 14
5.5 Allele-specific copy number estimates . . . 15

6 Region: TCGA-23-1027:Chr10@106.5-113.5,cp=110+/-0.5,s=2/3 **16**
6.1 Decrease in Heterozygosity (DH) and total copy-number tracks . . . 16
6.2 Allele B fraction density plots . . . 17
6.3 ROC curves . . . 17
6.4 (β_N, β_T) plots . . . 18
6.5 Allele-specific copy number estimates . . . 19

7 Region: TCGA-23-1027:Chr2@55-75.0,cp=65.0+/-0.5,s=0/1 **20**
7.1 Decrease in Heterozygosity (DH) and total copy-number tracks . . . 20
7.2 Allele B fraction density plots . . . 21
7.3 ROC curves . . . 21
7.4 (β_N, β_T) plots . . . 22
7.5 Allele-specific copy number estimates . . . 23

8 Bootstrap estimates of test statistics for all regions **24**

A Data files **26**
A.1 Total copy numbers 26
A.2 Allele B fractions 26
A.3 Genotype calls 26

B Session information **28**

1 Introduction

This report, which is automatically generated, assesses the performance of the TumorBoost method based on a few change points in a particular tumor/normal pair. For more details on the evaluation methods, see the main TumorBoost manuscript.

2 Data set

The evaluation is this report is based on the tumor/normal pair (01A,10A) for individual TCGA-23-1027 in the data set TCGA,OV,Birdseed,ismpolish.

2.1 Preprocessing methods

The data was generated on the Affymetrix GenomeWideSNP_6 chip type.

There is one CEL file per hybridization. The CEL files were preprocessed using Birdseed/median polish Korn *et al.* (2008) as part of a larger batch of CEL files.

2.2 Stratification on genotype confidence scores

No stratification on genotype confidence scores is done. All heterozygous SNPs are used in this evaluation.

2.3 List of change points

For this data set, we have selected a few regions for which one can safely assume that there exists a single copy number change point. By definition, each change point separates two sets of genomic loci such that the true Decrease in Heterozygosity (DH) is the same within one set of loci but differs between the two sets. These regions were selected visually. For each region we chose a large enough safety margin to make our evaluation independent of the uncertainty on the true location of the change point.

Chr	Region	Change point	Margin	Before	After
2	108-140	124	0.5	'normal' (1,1)	'gain' (1,2)
2	125-157	141	0.5	'gain' (1,2)	'copy neutral LOH' (0,2)
10	80-109	94	0.5	'normal' (1,1)	'deletion' (0,1)
10	106.5-113.5	110	0.5	'deletion' (0,1)	'copy neutral LOH' (0,2)
2	55-75	65	0.5	'normal' (1,1)	'gain' (1,2)

Table 1: Regions in TCGA-23-1027 used for the evaluation and that each contain a single changepoint. All positions and lengths are in units of Mb.

We next compare how well each of these change points is detected using the above preprocessed signals followed or not by TumorBoost normalization using the ROC analysis described in the main TumorBoost manuscript at the full resolution as well as smoothed resolution with bin sizes $h = \{1, 2, 4\}$. Specifically, we compare the following three methods: (1) **"raw"**: preprocessed signals without TumorBoost normalization. (2) **"TBN,Birdseed"**: preprocessed signals followed by TumorBoost normalization using Birdseed genotype calls. (3) **"TBN,NGC"**: preprocessed signals followed by TumorBoost normalization using NGC genotype calls. For completeness we also include an evaluation of Total copy numbers (TCN).

3 Region: TCGA-23-1027:Chr2@108-140,cp=124+/-0.5,s=0/1

3.1 Decrease in Heterozygosity (DH) and total copy-number tracks

(a) raw,Birdseed.

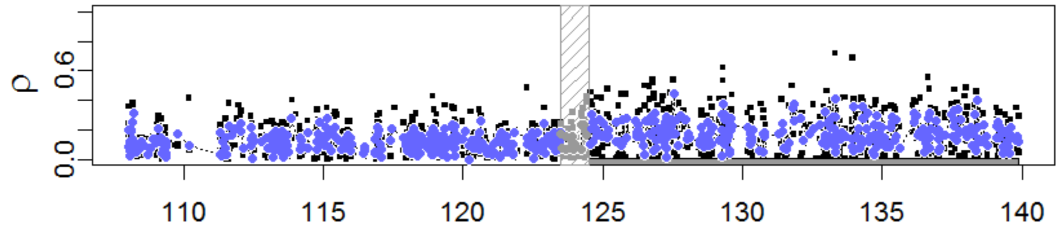

(b) TBN,Birdseed,Birdseed.

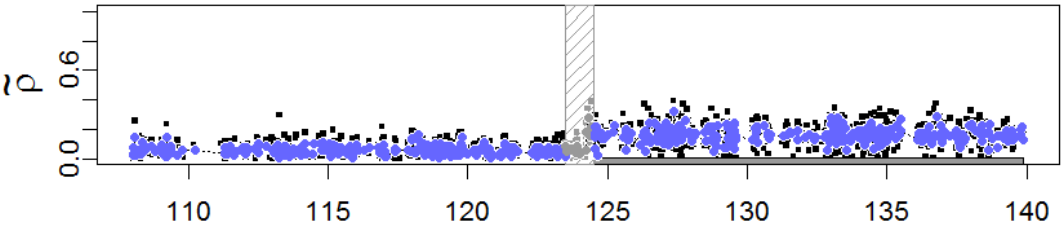

(c) TBN,NGC,NGC.

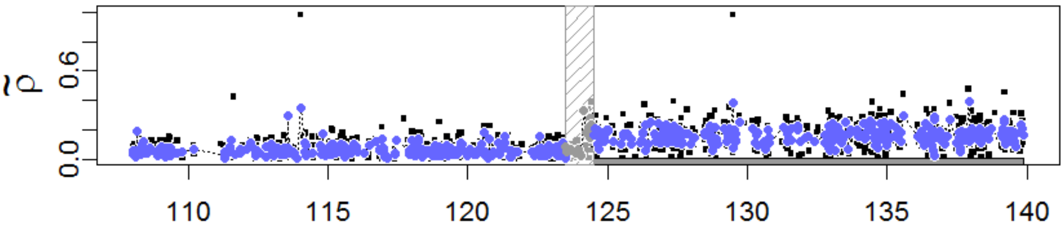

(d) TCN,Birdseed.

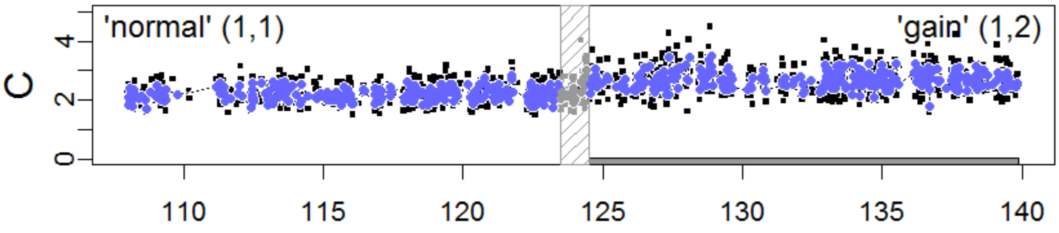

Figure 1: Decrease in Heterozygosity (DH) and total copy numbers for region TCGA-23-1027:Chr2@108-140,cp=124+/-0.5,s=0/1. Only heterozygous SNPs are plotted. There are 1171 loci of state 'normal' (1,1) ("negatives") and 1171 loci of state 'gain' (1,2) ("positives"), where the latter are highlighted with a solid bar beneath. In total 79 loci within the safety margin were excluded.

3.2 Allele B fraction density plots

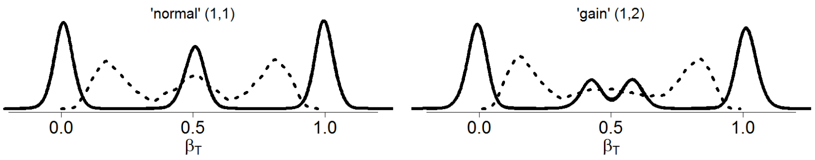

(a) 'normal' (1,1).

(b) 'gain' (1,2).

Figure 2: Density of raw (dashed lines) and TumorBoost-normalized (solid lines) allele B fractions for region TCGA-23-1027:Chr2@108-140,cp=124+/-0.5,s=0/1.

3.3 ROC curves

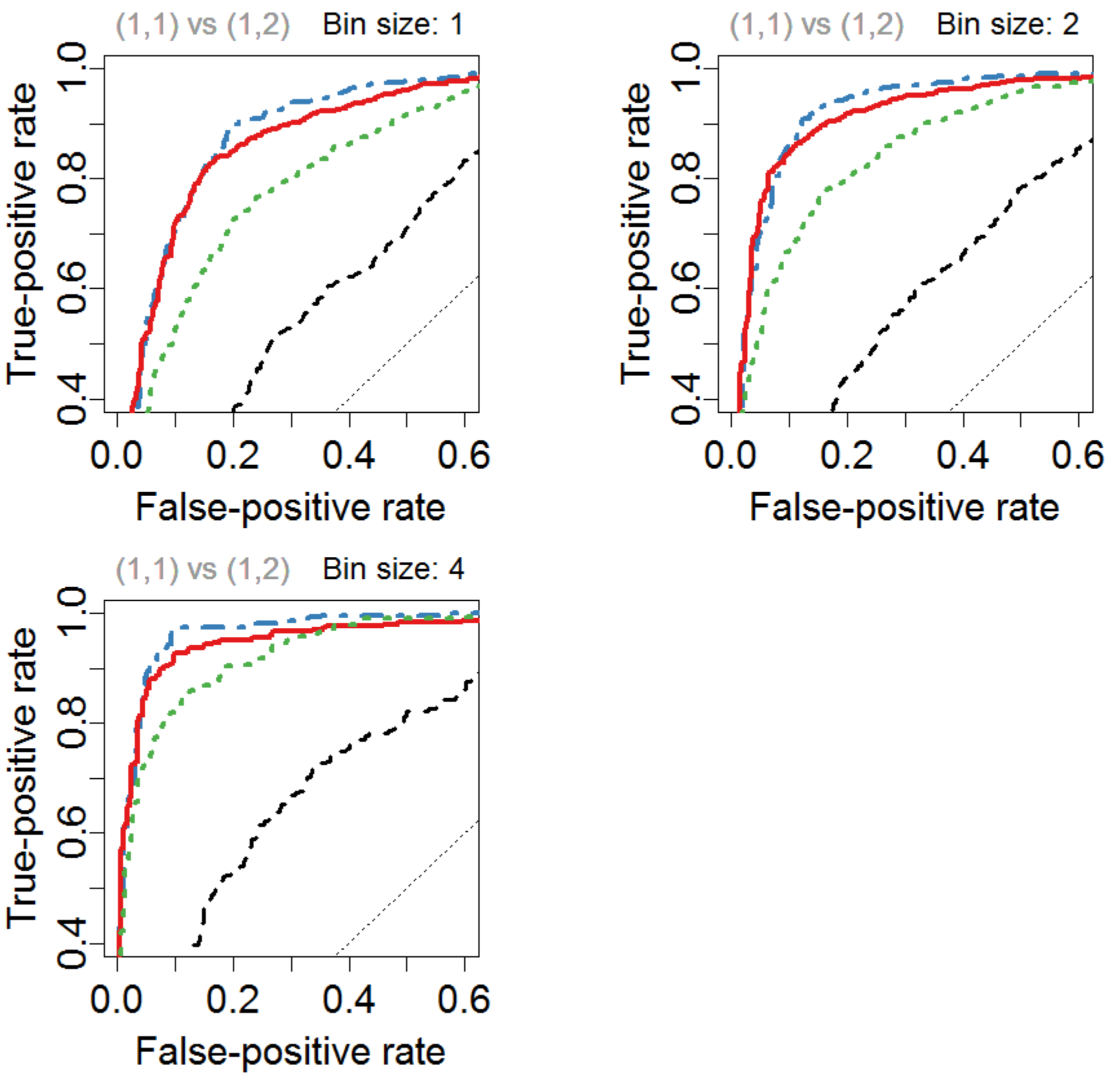

Figure 3: ROC curves for each preprocessing method at the full resolution as well as 2 different amounts of smoothing (using the mean() function) for region TCGA-23-1027:Chr2@108-140,cp=124+/-0.5,s=0/1. Legend: raw,Birdseed (dashed; #000000), TBN,Birdseed,Birdseed (dash-dotted; #377EB8), TBN,NGC,NGC (solid; #E41A1C) and TCN,Birdseed (dotted; #4DAF4A).

3.4 (β_N, β_T) plots

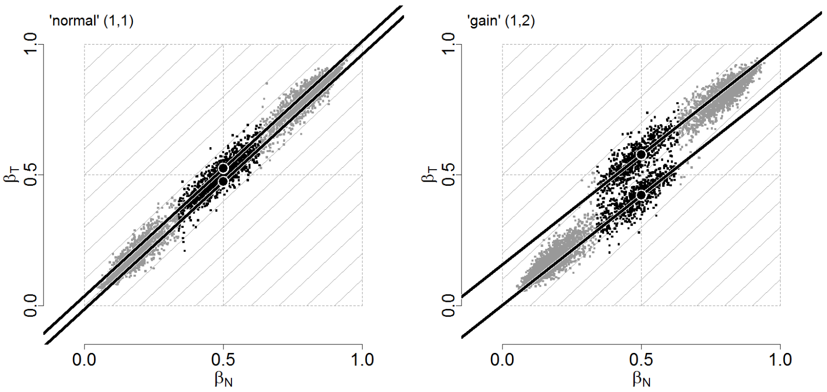

Figure 4: raw,NGC for region TCGA-23-1027:Chr2@108-140,cp=124+/-0.5,s=0/1.

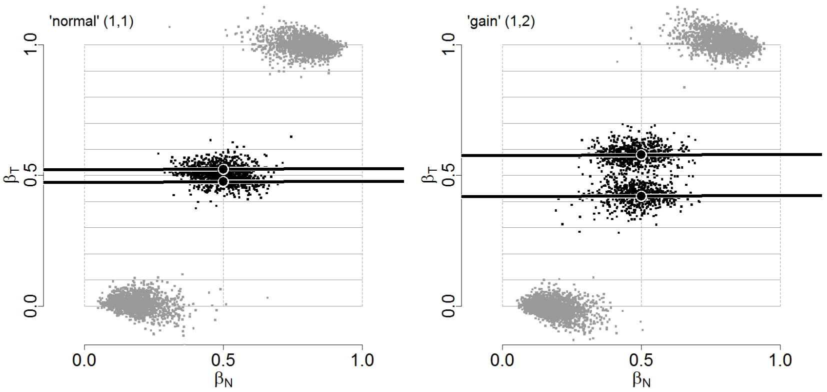

Figure 5: TBN,Birdseed,Birdseed for region TCGA-23-1027:Chr2@108-140,cp=124+/-0.5,s=0/1.

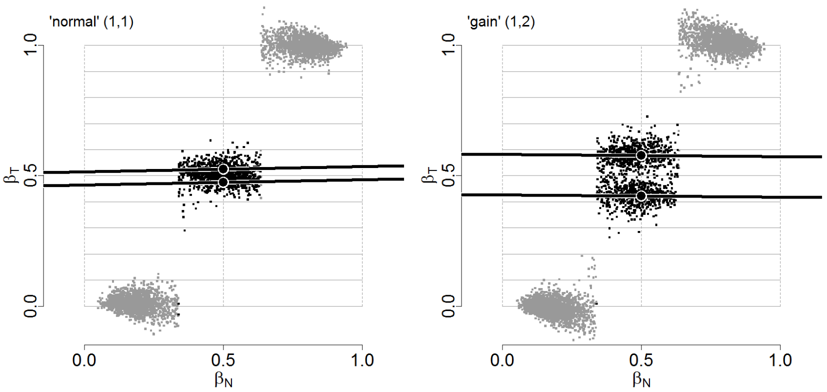

Figure 6: TBN,NGC,NGC for region TCGA-23-1027:Chr2@108-140,cp=124+/-0.5,s=0/1.

3.5 Allele-specific copy number estimates

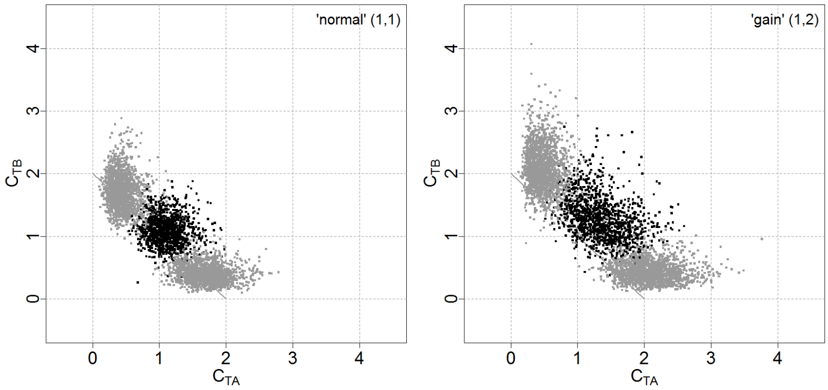

Figure 7: raw,NGC for region TCGA-23-1027:Chr2@108-140,cp=124+/-0.5,s=0/1.

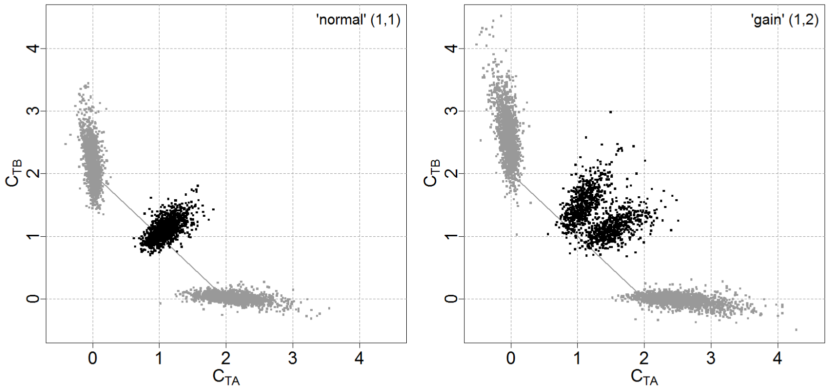

Figure 8: TBN,Birdseed,Birdseed for region TCGA-23-1027:Chr2@108-140,cp=124+/-0.5,s=0/1.

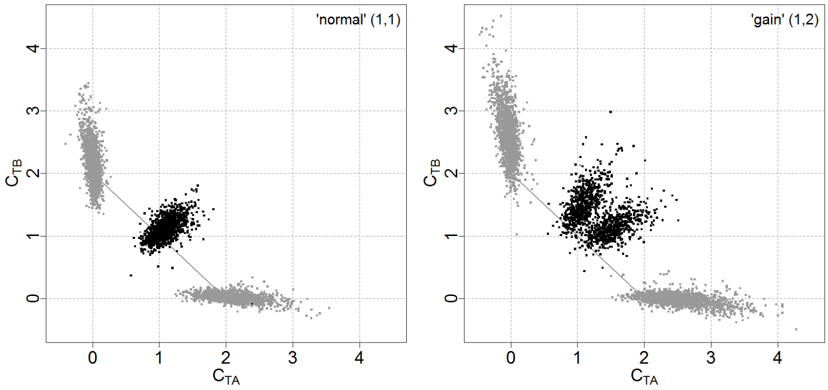

Figure 9: TBN,NGC,NGC for region TCGA-23-1027:Chr2@108-140,cp=124+/-0.5,s=0/1.

4 Region: TCGA-23-1027:Chr2@125.0-157.0,cp=141.0+/-0.5,s=1/3

4.1 Decrease in Heterozygosity (DH) and total copy-number tracks

(a) raw,Birdseed.

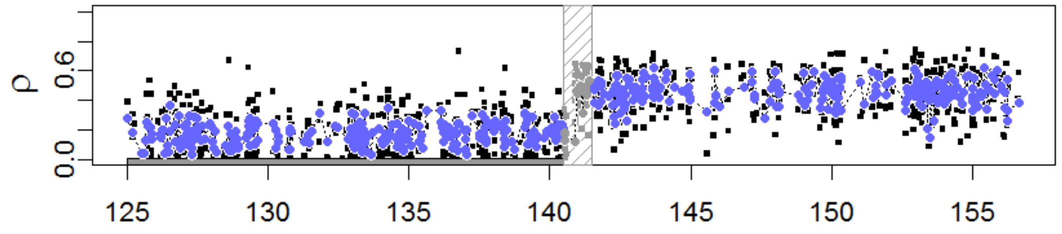

(b) TBN,Birdseed,Birdseed.

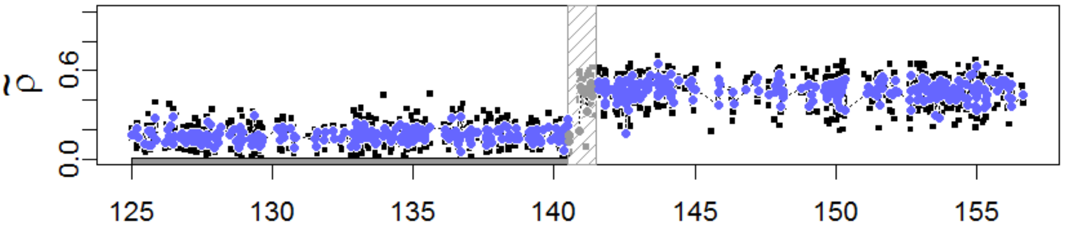

(c) TBN,NGC,NGC.

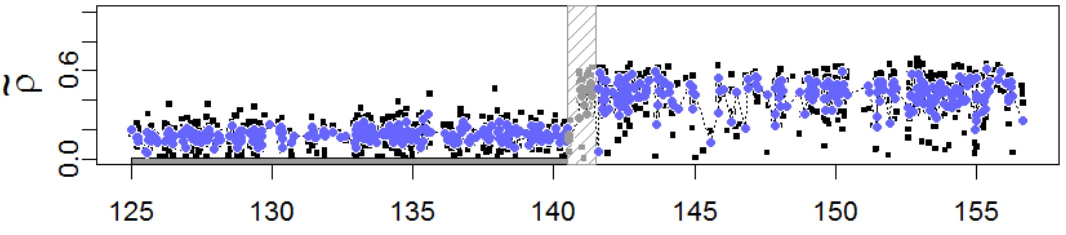

(d) TCN,Birdseed.

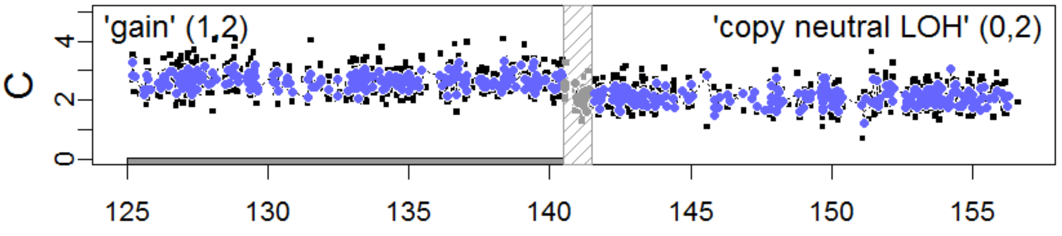

Figure 10: Decrease in Heterozygosity (DH) and total copy numbers for region TCGA-23-1027:Chr2@125.0-157.0,cp=141.0+/-0.5,s=1/3. Only heterozygous SNPs are plotted. There are 986 loci of state 'gain' (1,2) ("negatives") and 986 loci of state 'copy neutral LOH' (0,2) ("positives"), where the latter are highlighted with a solid bar beneath. In total 64 loci within the safety margin were excluded.

4.2 Allele B fraction density plots

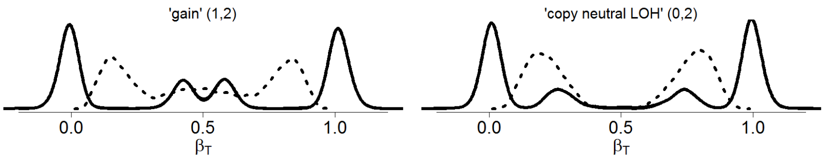

(a) 'gain' (1,2). (b) 'copy neutral LOH' (0,2).

Figure 11: Density of raw (dashed lines) and TumorBoost-normalized (solid lines) allele B fractions for region TCGA-23-1027:Chr2@125.0-157.0,cp=141.0+/-0.5,s=1/3.

4.3 ROC curves

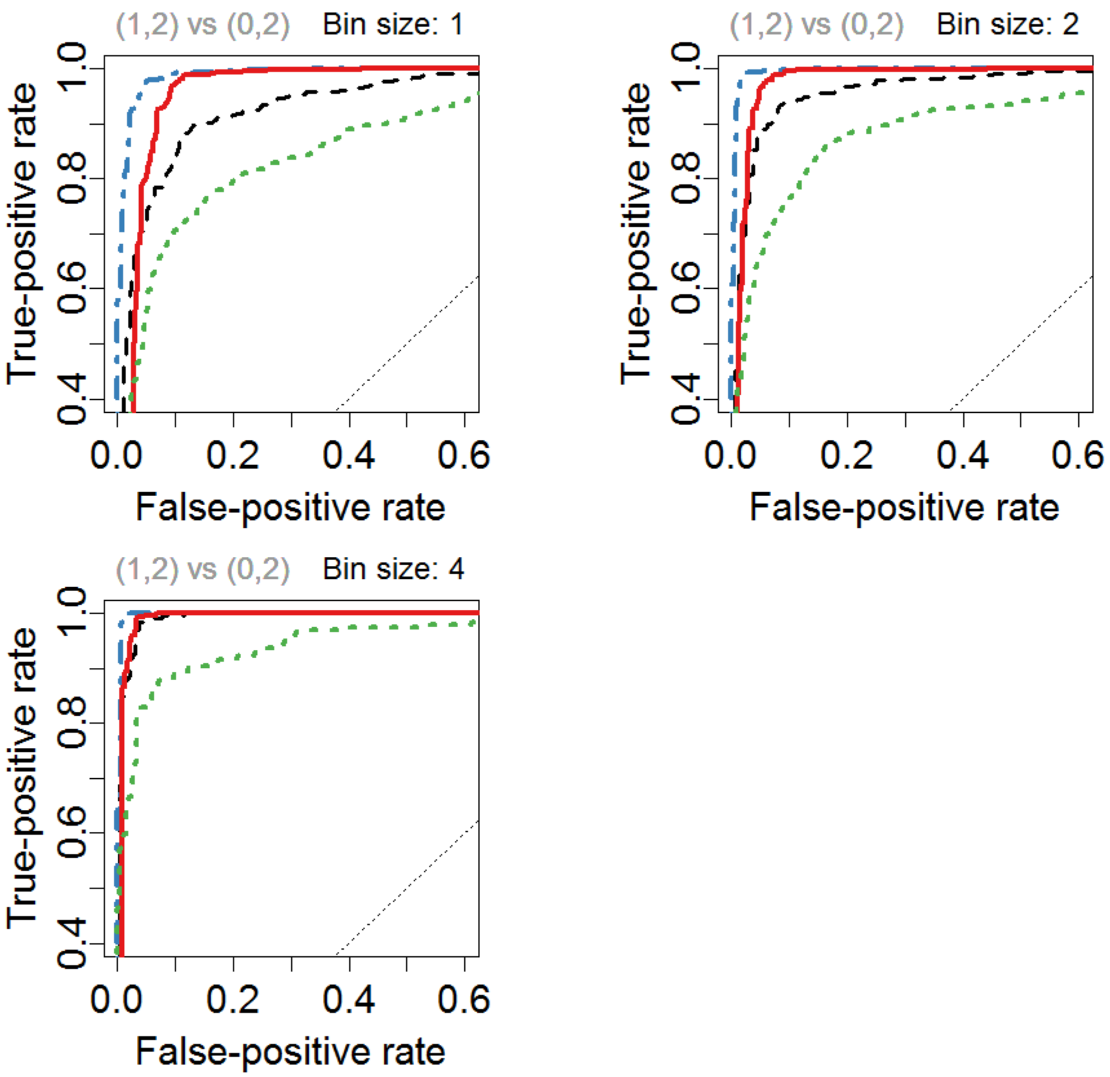

Figure 12: ROC curves for each preprocessing method at the full resolution as well as 2 different amounts of smoothing (using the mean() function) for region TCGA-23-1027:Chr2@125.0-157.0,cp=141.0+/-0.5,s=1/3. Legend: raw,Birdseed (dashed; #000000), TBN,Birdseed,Birdseed (dash-dotted; #377EB8), TBN,NGC,NGC (solid; #E41A1C) and TCN,Birdseed (dotted; #4DAF4A).

4.4 (β_N, β_T) plots

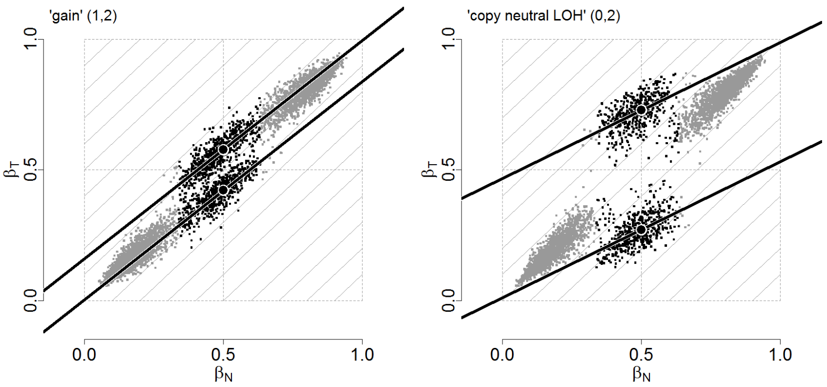

Figure 13: raw,NGC for region TCGA-23-1027:Chr2@125.0-157.0,cp=141.0+/-0.5,s=1/3.

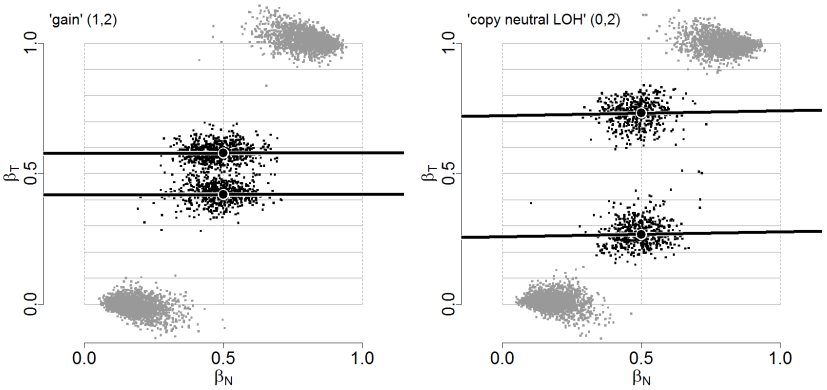

Figure 14: TBN,Birdseed,Birdseed for region TCGA-23-1027:Chr2@125.0-157.0,cp=141.0+/-0.5,s=1/3.

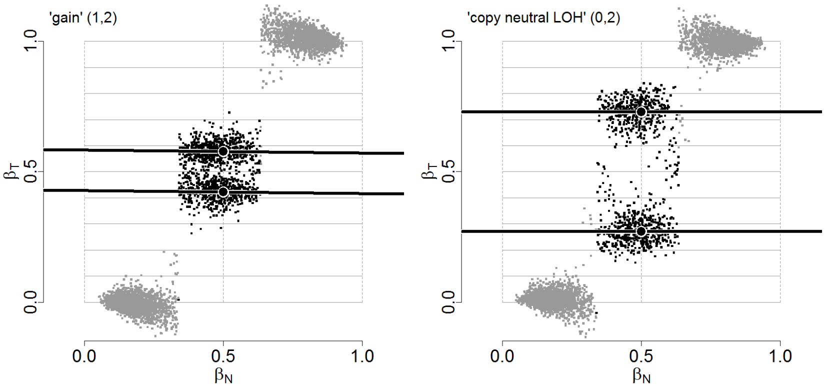

Figure 15: TBN,NGC,NGC for region TCGA-23-1027:Chr2@125.0-157.0,cp=141.0+/-0.5,s=1/3.

4.5 Allele-specific copy number estimates

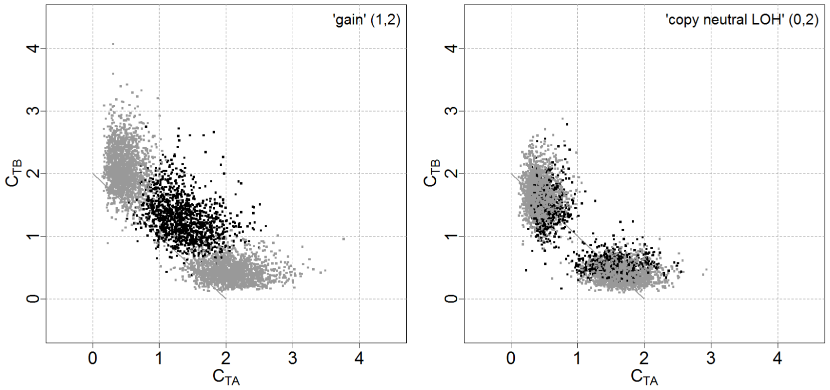

Figure 16: raw,NGC for region TCGA-23-1027:Chr2@125.0-157.0,cp=141.0+/-0.5,s=1/3.

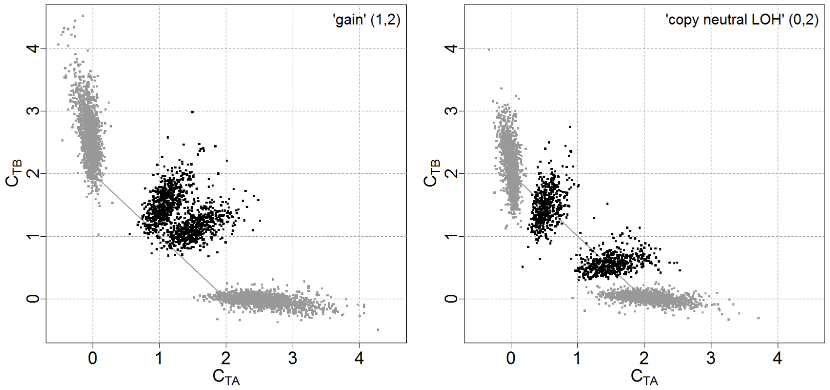

Figure 17: TBN,Birdseed,Birdseed for region TCGA-23-1027:Chr2@125.0-157.0,cp=141.0+/-0.5,s=1/3.

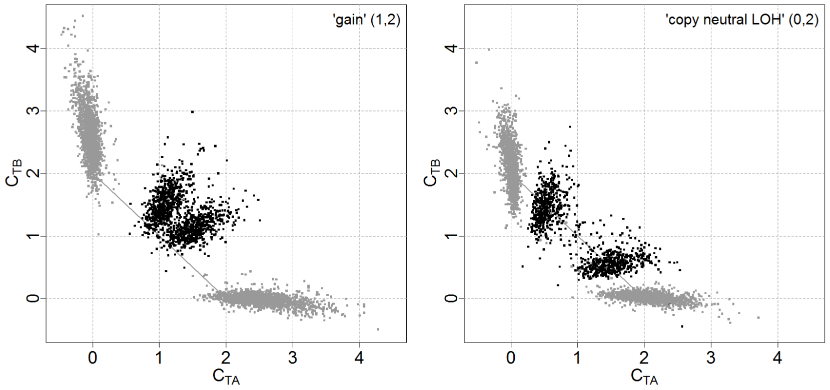

Figure 18: TBN,NGC,NGC for region TCGA-23-1027:Chr2@125.0-157.0,cp=141.0+/-0.5,s=1/3.

5 Region: TCGA-23-1027:Chr10@80-109,cp=94+/-0.5,s=0/2

5.1 Decrease in Heterozygosity (DH) and total copy-number tracks

(a) raw,Birdseed.

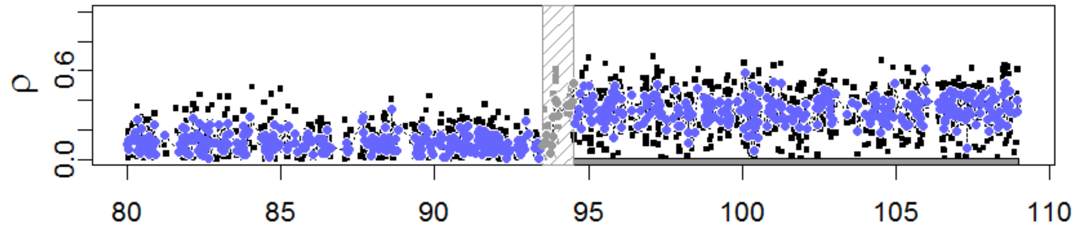

(b) TBN,Birdseed,Birdseed.

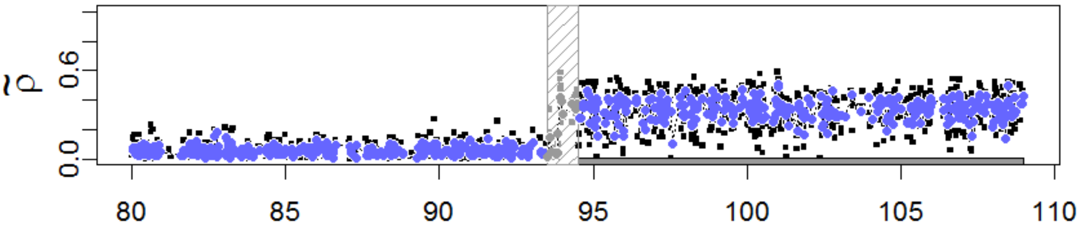

(c) TBN,NGC,NGC.

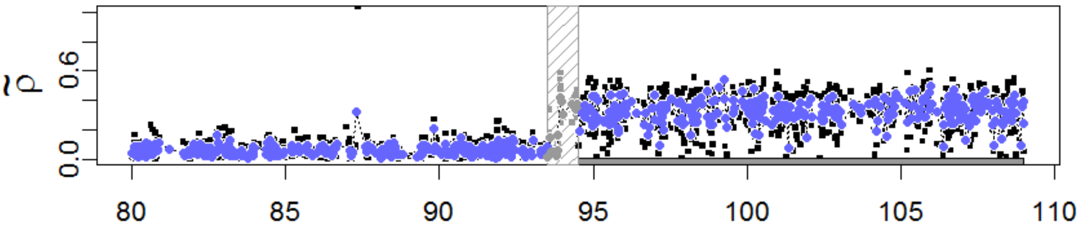

(d) TCN,Birdseed.

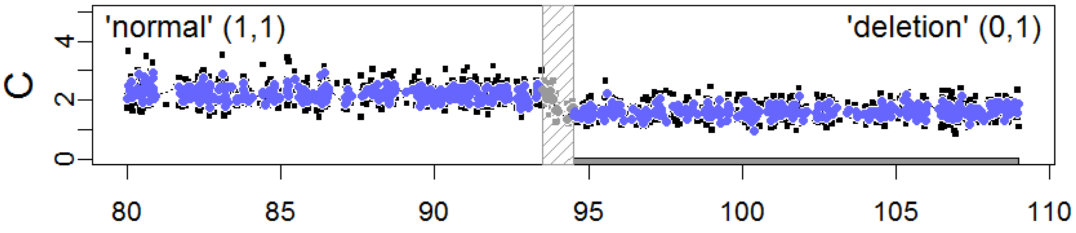

Figure 19: Decrease in Heterozygosity (DH) and total copy numbers for region TCGA-23-1027:Chr10@80-109,cp=94+/-0.5,s=0/2. Only heterozygous SNPs are plotted. There are 1276 loci of state 'normal' (1,1) ("negatives") and 1276 loci of state 'deletion' (0,1) ("positives"), where the latter are highlighted with a solid bar beneath. In total 53 loci within the safety margin were excluded.

5.2 Allele B fraction density plots

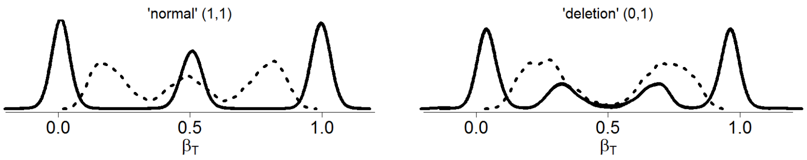

(a) 'normal' (1,1). (b) 'deletion' (0,1).

Figure 20: Density of raw (dashed lines) and TumorBoost-normalized (solid lines) allele B fractions for region TCGA-23-1027:Chr10@80-109,cp=94+/-0.5,s=0/2.

5.3 ROC curves

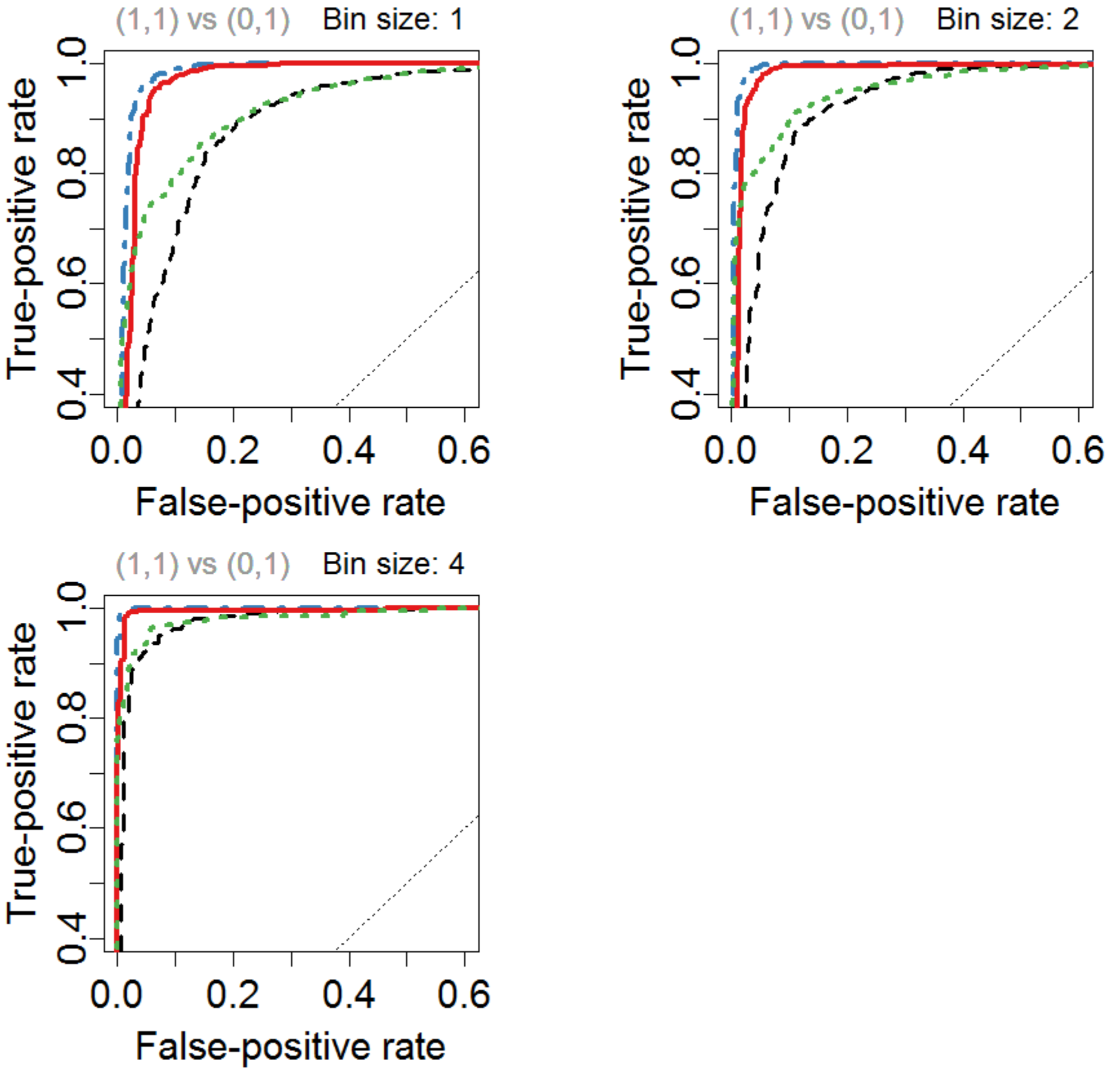

Figure 21: ROC curves for each preprocessing method at the full resolution as well as 2 different amounts of smoothing (using the mean() function) for region TCGA-23-1027:Chr10@80-109,cp=94+/-0.5,s=0/2. Legend: raw,Birdseed (dashed; #000000), TBN,Birdseed,Birdseed (dash-dotted; #377EB8), TBN,NGC,NGC (solid; #E41A1C) and TCN,Birdseed (dotted; #4DAF4A).

5.4 (β_N, β_T) plots

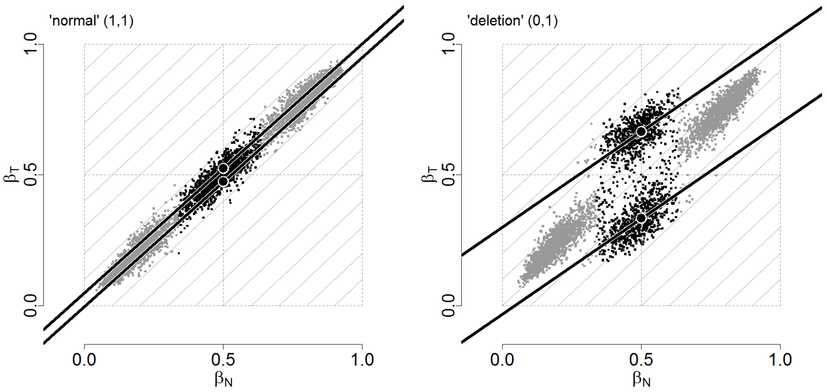

Figure 22: raw,NGC for region TCGA-23-1027:Chr10@80-109,cp=94+/-0.5,s=0/2.

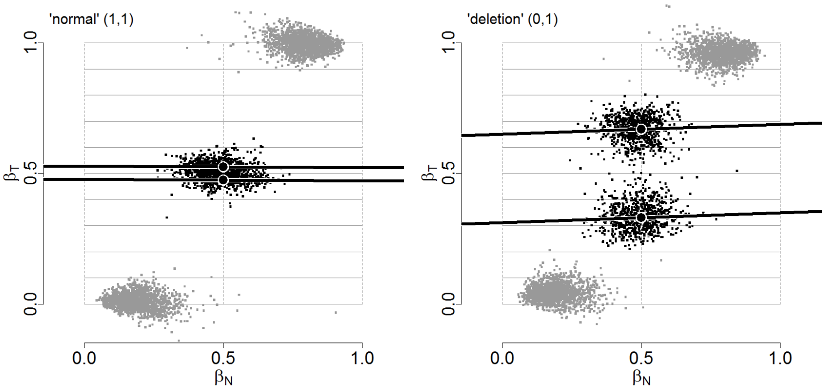

Figure 23: TBN,Birdseed,Birdseed for region TCGA-23-1027:Chr10@80-109,cp=94+/-0.5,s=0/2.

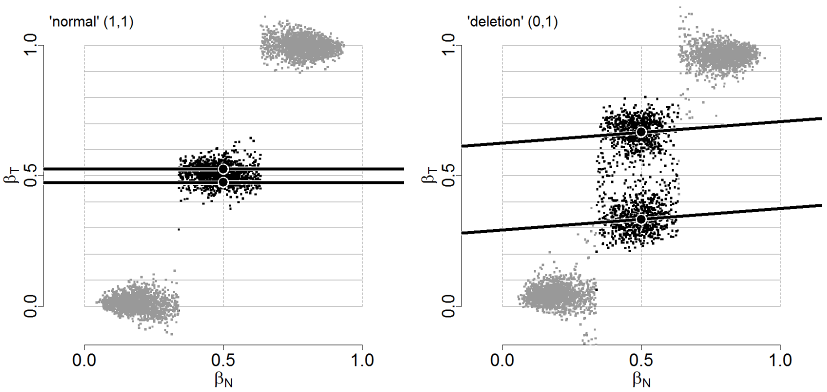

Figure 24: TBN,NGC,NGC for region TCGA-23-1027:Chr10@80-109,cp=94+/-0.5,s=0/2.

5.5 Allele-specific copy number estimates

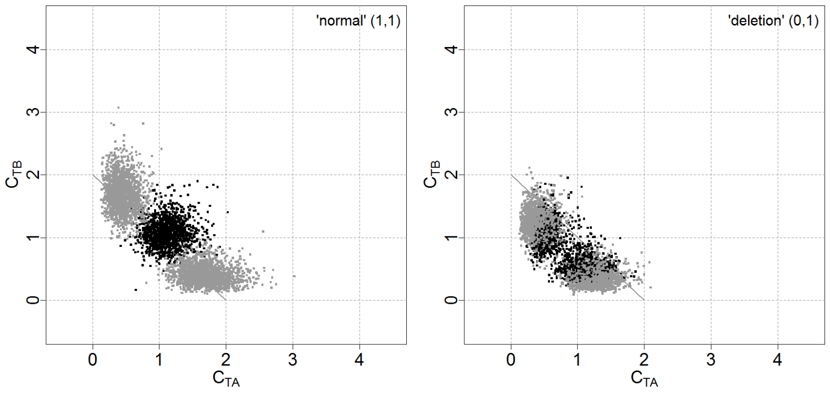

Figure 25: raw,NGC for region TCGA-23-1027:Chr10@80-109,cp=94+/-0.5,s=0/2.

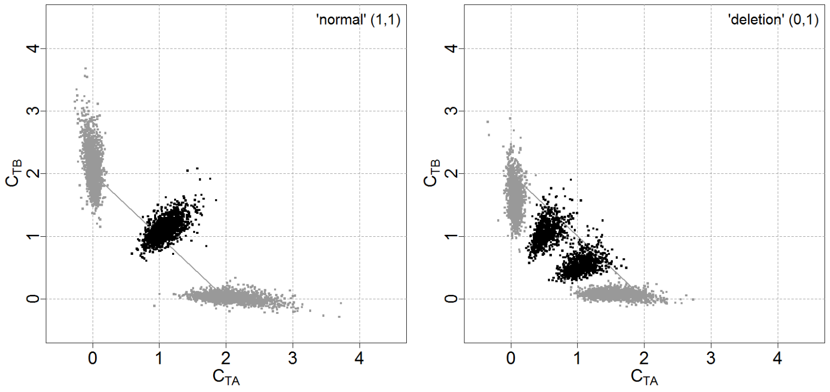

Figure 26: TBN,Birdseed,Birdseed for region TCGA-23-1027:Chr10@80-109,cp=94+/-0.5,s=0/2.

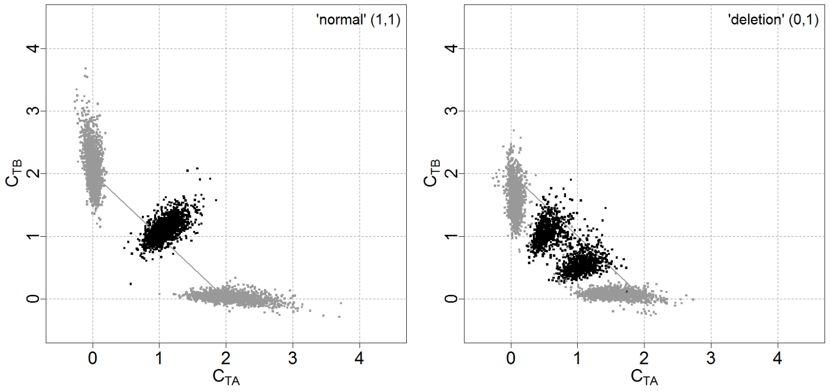

Figure 27: TBN,NGC,NGC for region TCGA-23-1027:Chr10@80-109,cp=94+/-0.5,s=0/2.

6 Region: TCGA-23-1027:Chr10@106.5-113.5,cp=110+/-0.5,s=2/3

6.1 Decrease in Heterozygosity (DH) and total copy-number tracks

(a) raw,Birdseed.

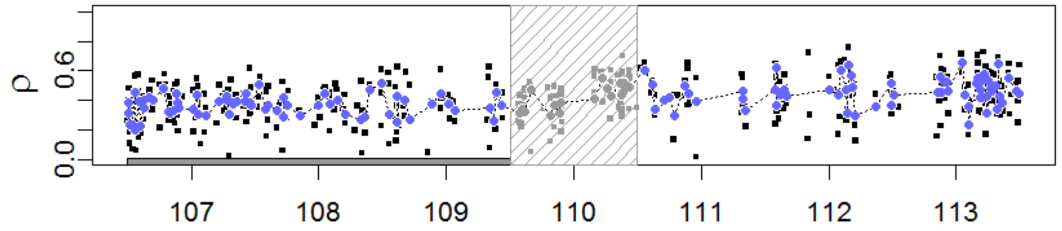

(b) TBN,Birdseed,Birdseed.

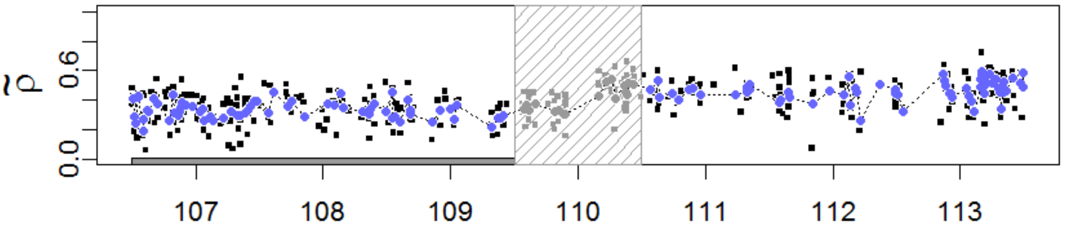

(c) TBN,NGC,NGC.

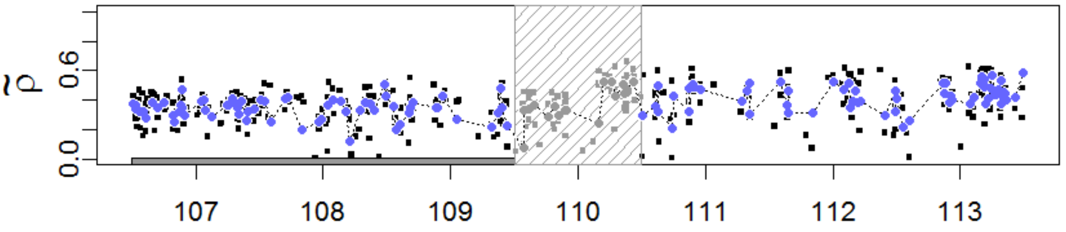

(d) TCN,Birdseed.

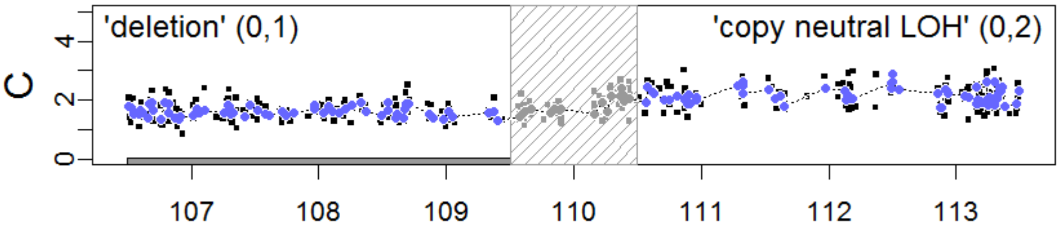

Figure 28: Decrease in Heterozygosity (DH) and total copy numbers for region TCGA-23-1027:Chr10@106.5-113.5,cp=110+/-0.5,s=2/3. Only heterozygous SNPs are plotted. There are 254 loci of state 'deletion' (0,1) ("negatives") and 254 loci of state 'copy neutral LOH' (0,2) ("positives"), where the latter are highlighted with a solid bar beneath. In total 69 loci within the safety margin were excluded.

6.2 Allele B fraction density plots

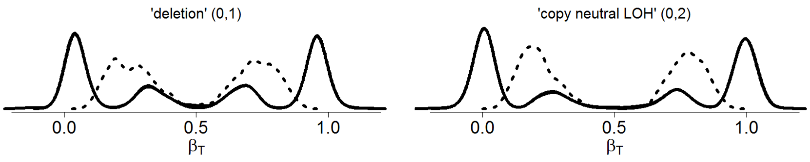

(a) 'deletion' (0,1). (b) 'copy neutral LOH' (0,2).

Figure 29: Density of raw (dashed lines) and TumorBoost-normalized (solid lines) allele B fractions for region TCGA-23-1027:Chr10@106.5-113.5,cp=110+/-0.5,s=2/3.

6.3 ROC curves

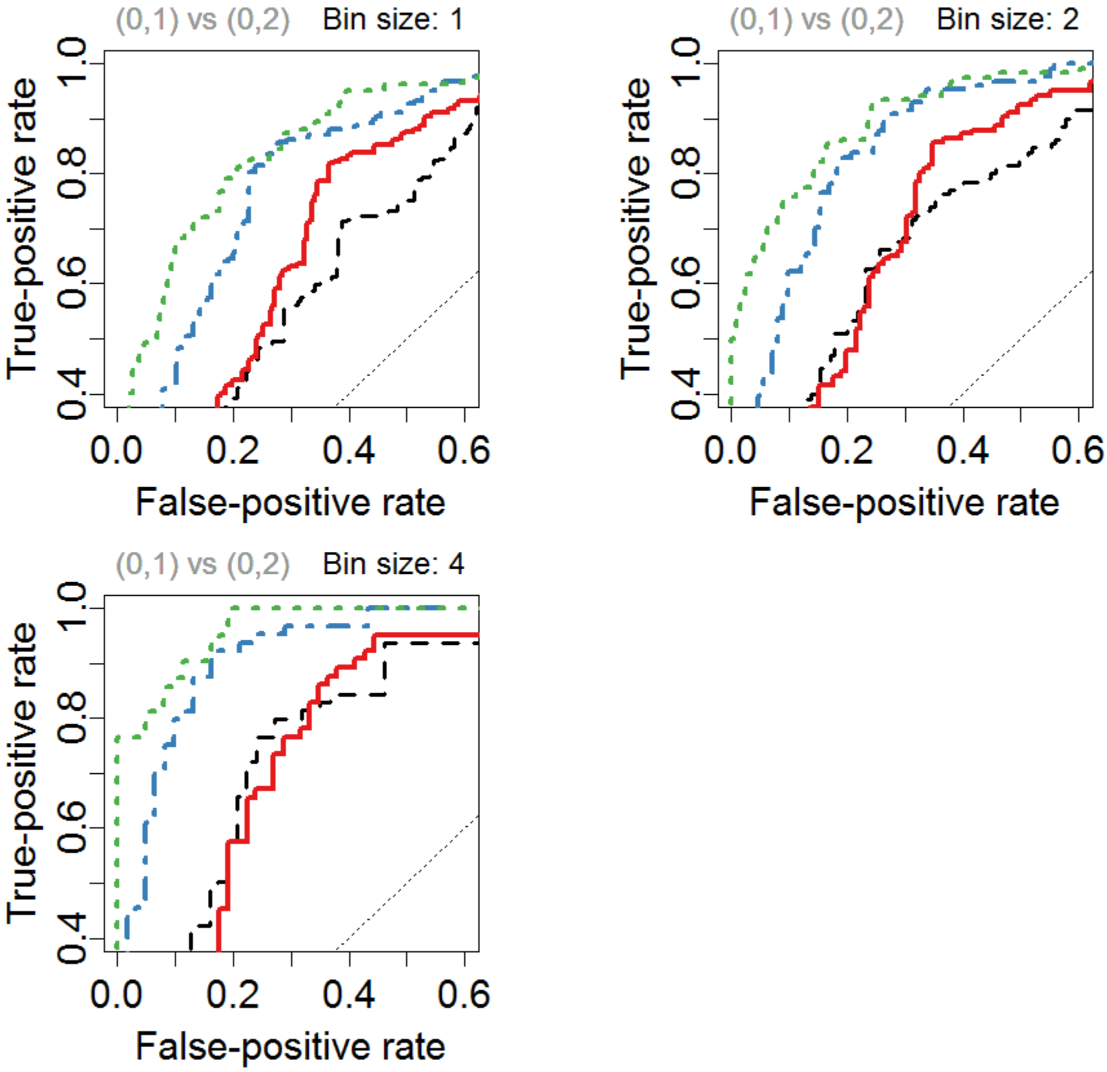

Figure 30: ROC curves for each preprocessing method at the full resolution as well as 2 different amounts of smoothing (using the mean() function) for region TCGA-23-1027:Chr10@106.5-113.5,cp=110+/-0.5,s=2/3. Legend: raw,Birdseed (dashed; #000000), TBN,Birdseed,Birdseed (dash-dotted; #377EB8), TBN,NGC,NGC (solid; #E41A1C) and TCN,Birdseed (dotted; #4DAF4A).

6.4 (β_N, β_T) plots

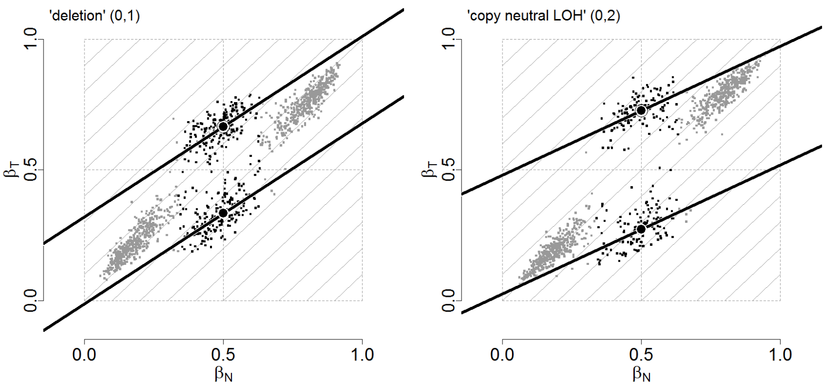

Figure 31: raw,NGC for region TCGA-23-1027:Chr10@106.5-113.5,cp=110+/-0.5,s=2/3.

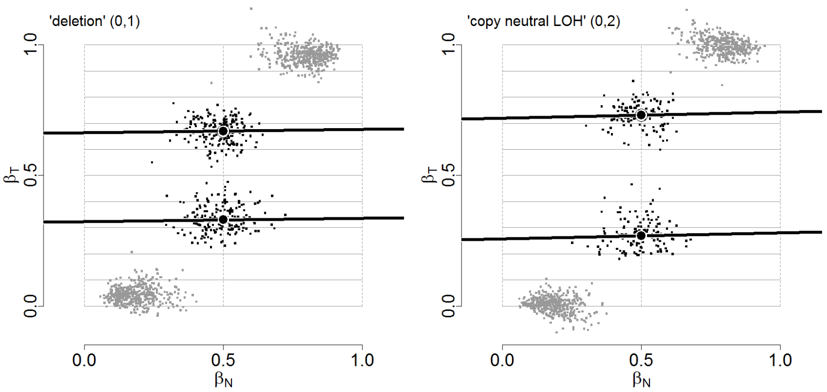

Figure 32: TBN,Birdseed,Birdseed for region TCGA-23-1027:Chr10@106.5-113.5,cp=110+/-0.5,s=2/3.

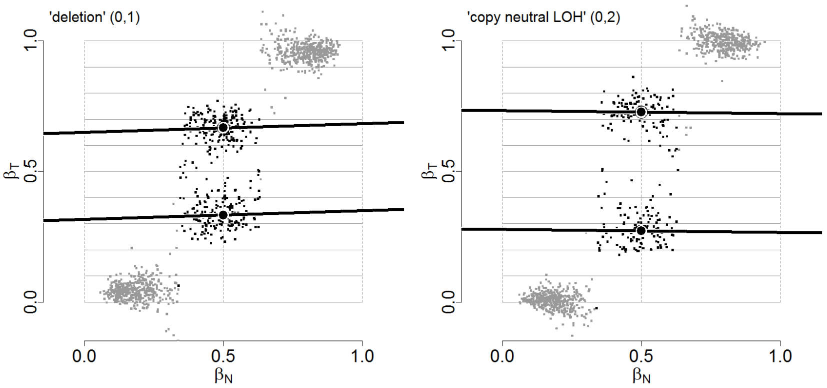

Figure 33: TBN,NGC,NGC for region TCGA-23-1027:Chr10@106.5-113.5,cp=110+/-0.5,s=2/3.

6.5 Allele-specific copy number estimates

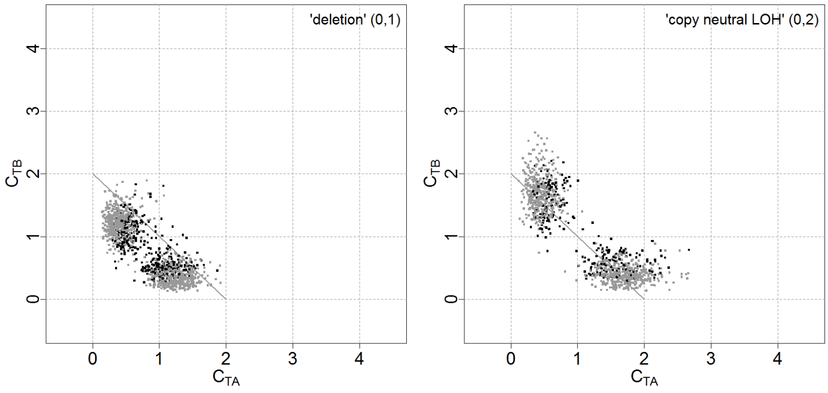

Figure 34: raw,NGC for region TCGA-23-1027:Chr10@106.5-113.5,cp=110+/-0.5,s=2/3.

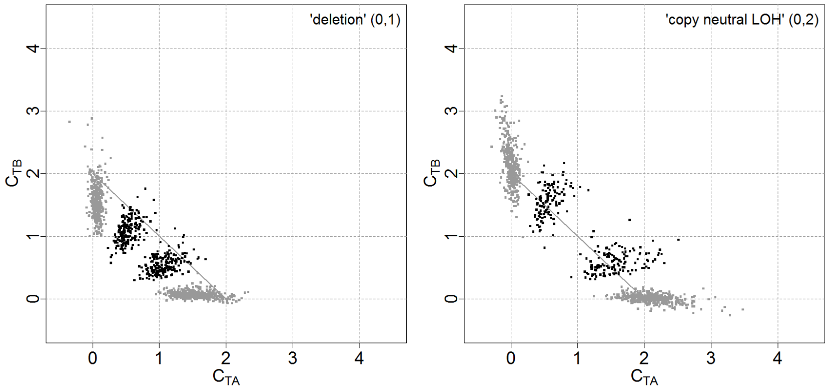

Figure 35: TBN,Birdseed,Birdseed for region TCGA-23-1027:Chr10@106.5-113.5,cp=110+/-0.5,s=2/3.

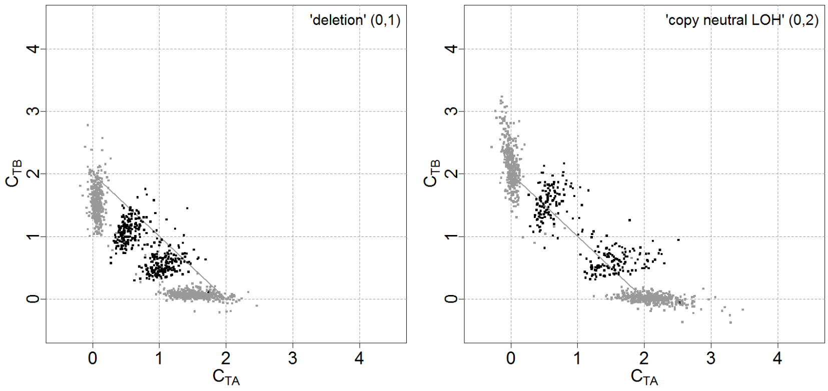

Figure 36: TBN,NGC,NGC for region TCGA-23-1027:Chr10@106.5-113.5,cp=110+/-0.5,s=2/3.

7 Region: TCGA-23-1027:Chr2@55-75.0,cp=65.0+/-0.5,s=0/1

7.1 Decrease in Heterozygosity (DH) and total copy-number tracks

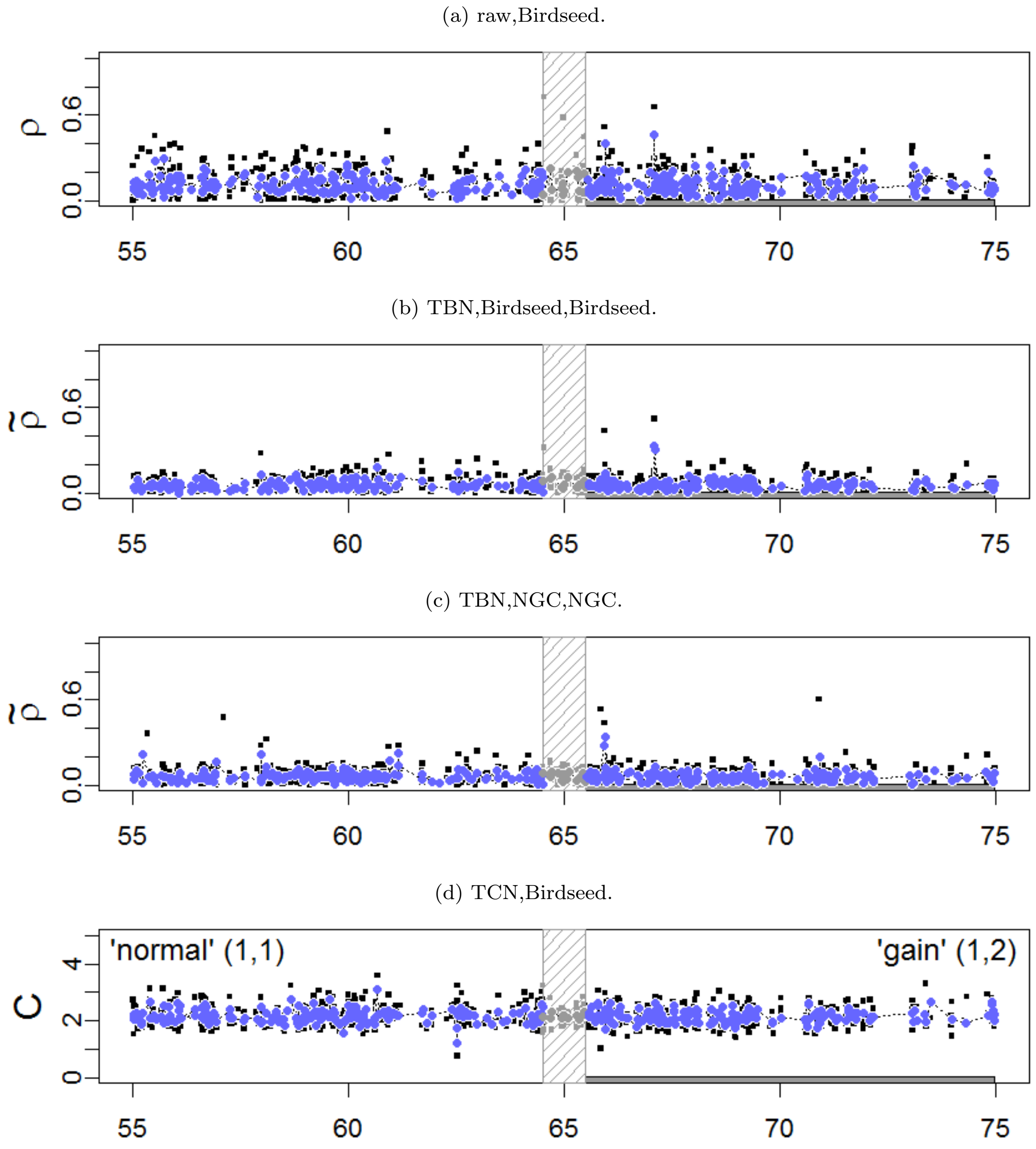

Figure 37: Decrease in Heterozygosity (DH) and total copy numbers for region TCGA-23-1027:Chr2@55-75.0,cp=65.0+/-0.5,s=0/1. Only heterozygous SNPs are plotted. There are 713 loci of state 'normal' (1,1) ("negatives") and 713 loci of state 'gain' (1,2) ("positives"), where the latter are highlighted with a solid bar beneath. In total 60 loci within the safety margin were excluded.

7.2 Allele B fraction density plots

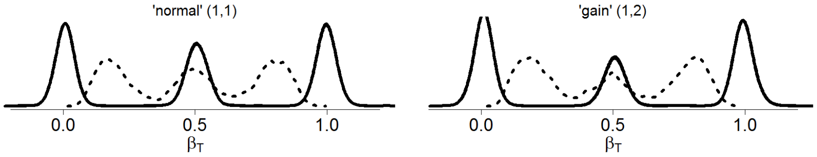

(a) 'normal' (1,1). (b) 'gain' (1,2).

Figure 38: Density of raw (dashed lines) and TumorBoost-normalized (solid lines) allele B fractions for region TCGA-23-1027:Chr2@55-75.0,cp=65.0+/-0.5,s=0/1.

7.3 ROC curves

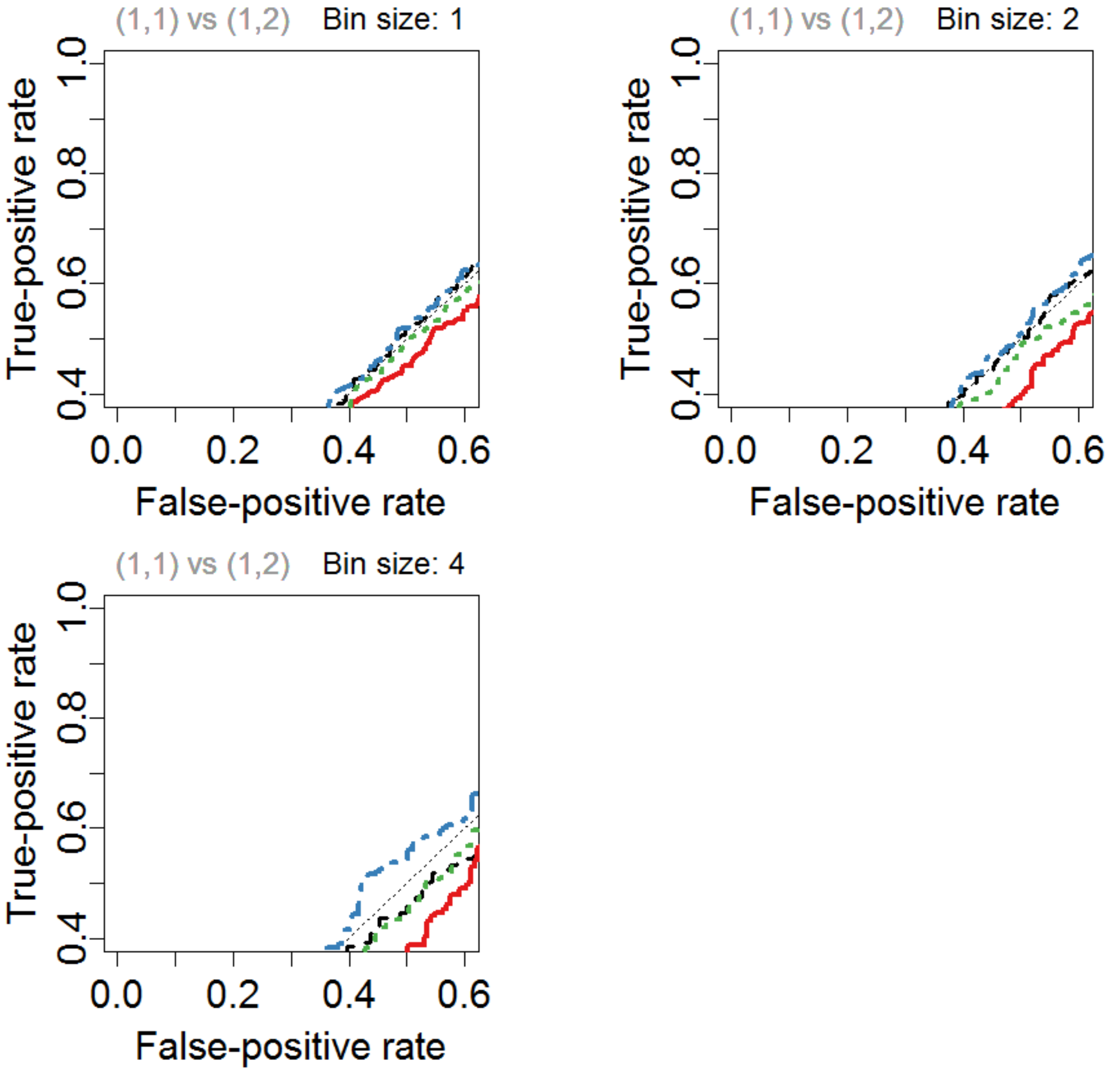

Figure 39: ROC curves for each preprocessing method at the full resolution as well as 2 different amounts of smoothing (using the mean() function) for region TCGA-23-1027:Chr2@55-75.0,cp=65.0+/-0.5,s=0/1. Legend: raw,Birdseed (dashed; #000000), TBN,Birdseed,Birdseed (dash-dotted; #377EB8), TBN,NGC,NGC (solid; #E41A1C) and TCN,Birdseed (dotted; #4DAF4A).

7.4 (β_N, β_T) plots

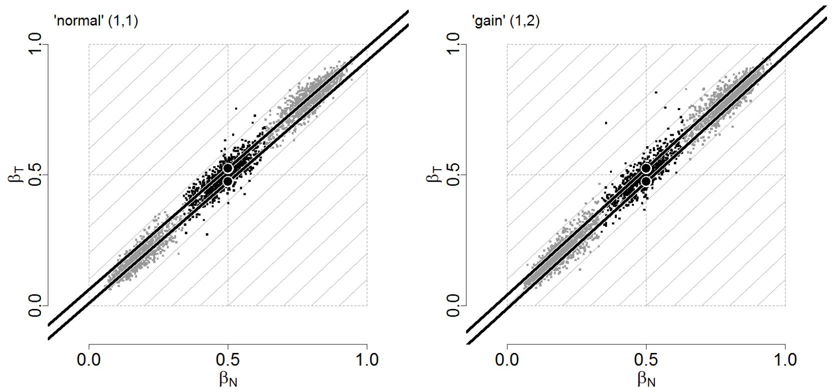

Figure 40: raw,NGC for region TCGA-23-1027:Chr2@55-75.0,cp=65.0+/-0.5,s=0/1.

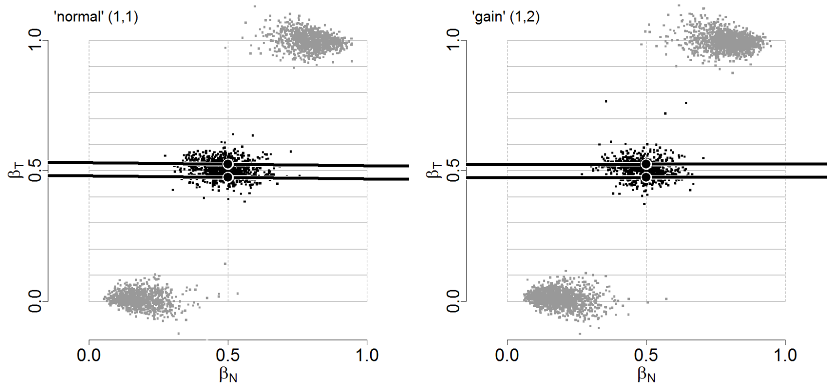

Figure 41: TBN,Birdseed,Birdseed for region TCGA-23-1027:Chr2@55-75.0,cp=65.0+/-0.5,s=0/1.

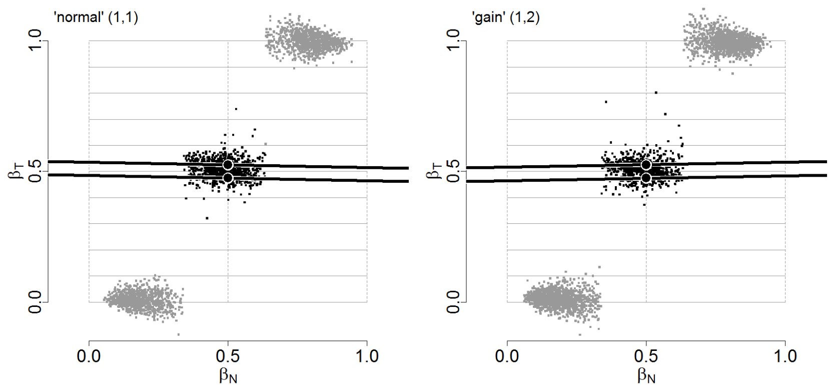

Figure 42: TBN,NGC,NGC for region TCGA-23-1027:Chr2@55-75.0,cp=65.0+/-0.5,s=0/1.

7.5 Allele-specific copy number estimates

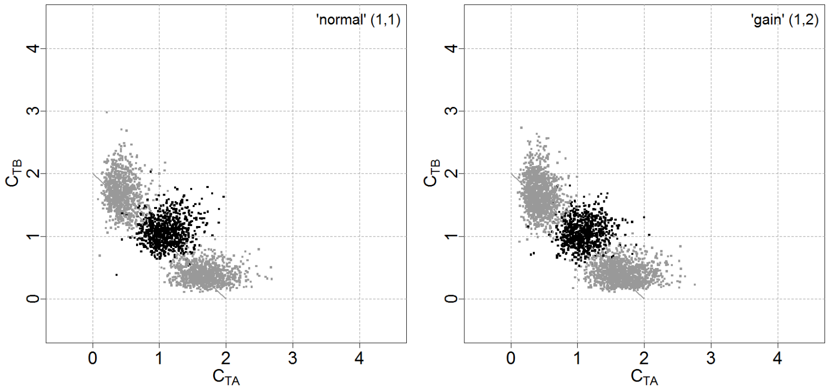

Figure 43: raw,NGC for region TCGA-23-1027:Chr2@55-75.0,cp=65.0+/-0.5,s=0/1.

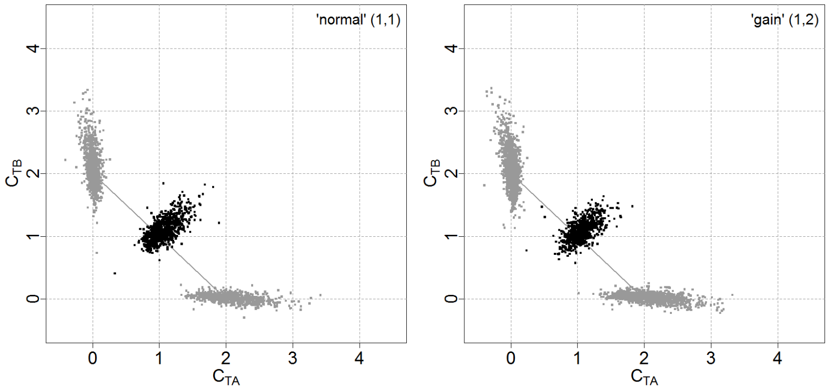

Figure 44: TBN,Birdseed,Birdseed for region TCGA-23-1027:Chr2@55-75.0,cp=65.0+/-0.5,s=0/1.

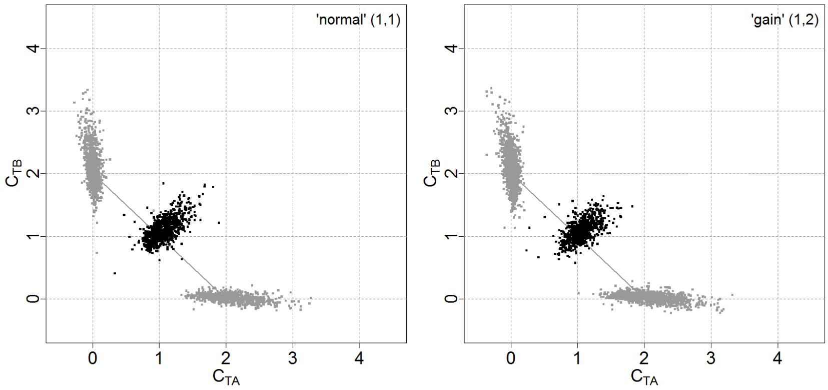

Figure 45: TBN,NGC,NGC for region TCGA-23-1027:Chr2@55-75.0,cp=65.0+/-0.5,s=0/1.

8 Bootstrap estimates of test statistics for all regions

	0/1	1/3	0/2	2/3	0/1
raw,Birdseed	6.338±1.112	25.089±1.722	21.472±1.223	9.674±1.102	0.901±0.665
TBN,Birdseed,Birdseed	20.888±1.302	39.171±2.180	40.371±1.972	13.284±1.177	0.795±0.603
TBN,NGC,NGC	16.826±2.923	31.473±2.553	33.912±2.968	10.838±1.213	0.890±0.648
TCN,Birdseed	14.244±1.013	17.150±1.249	22.369±1.281	18.413±1.053	1.588±0.882

Table 2: Student test statistics of the null hypothesis of equal mean before and after each PCN change point (heterozygous SNPs): raw or TumorBoost-normalized DH, and total copy number (last line). Mean ± standard deviation across 100 samplings of 250 points (for each PCN state) from the original data set. The larger value, the more different the true means are.

References

Korn, J. M., Kuruvilla, F. G., McCarroll, S. A., Wysoker, A., Nemesh, J., Cawley, S., Hubbell, E., Veitch, J., Collins, P. J., Darvishi, K., Lee, C., Nizzari, M. M., Gabriel, S. B., Purcell, S., Daly, M. J., and Altshuler, D. (2008). Integrated genotype calling and association analysis of SNPs, common copy number polymorphisms and rare CNVs. *Nature Genet.*, **40**(10), 1253–1260.

A Data files

A.1 Total copy numbers

$‘TCGA,OV,Birdseed,ismpolish‘
AromaUnitTotalCnBinarySet:
Name: TCGA
Tags: OV,Birdseed,ismpolish
Full name: TCGA,OV,Birdseed,ismpolish
Number of files: 1
Names: TCGA-23-1027
Path (to the first file): rawCnData/TCGA,OV,Birdseed,ismpolish/GenomeWideSNP˙6
Total file size: 7.08 MB
RAM: 0.00MB

A.2 Allele B fractions

$raw
AromaUnitFracBCnBinarySet:
Name: TCGA
Tags: OV,Birdseed,ismpolish
Full name: TCGA,OV,Birdseed,ismpolish
Number of files: 1
Names: TCGA-23-1027
Path (to the first file): totalAndFracBData/TCGA,OV,Birdseed,ismpolish/GenomeWideSNP˙6
Total file size: 7.08 MB
RAM: 0.00MB

$‘TBN,Birdseed‘
AromaUnitFracBCnBinarySet:
Name: TCGA
Tags: OV,Birdseed,ismpolish,TBN,Birdseed
Full name: TCGA,OV,Birdseed,ismpolish,TBN,Birdseed
Number of files: 1
Names: TCGA-23-1027
Path (to the first file): totalAndFracBData/TCGA,OV,Birdseed,ismpolish,TBN,Birdseed/GenomeWideSNP˙6
Total file size: 7.08 MB
RAM: 0.00MB

$‘TBN,NGC‘
AromaUnitFracBCnBinarySet:
Name: TCGA
Tags: OV,Birdseed,ismpolish,TBN,NGC
Full name: TCGA,OV,Birdseed,ismpolish,TBN,NGC
Number of files: 1
Names: TCGA-23-1027
Path (to the first file): totalAndFracBData/TCGA,OV,Birdseed,ismpolish,TBN,NGC/GenomeWideSNP˙6
Total file size: 7.08 MB
RAM: 0.00MB

A.3 Genotype calls

$Birdseed
AromaUnitGenotypeCallSet:
Name: TCGA
Tags: OV,Birdseed,ismpolish,Birdseed
Full name: TCGA,OV,Birdseed,ismpolish,Birdseed
Number of files: 1
Names: TCGA-23-1027

Path (to the first file): callData/TCGA,OV,Birdseed,ismpolish,Birdseed/GenomeWideSNP˙6
Total file size: 3.54 MB
RAM: 0.00MB

$NGC
AromaUnitGenotypeCallSet:
Name: TCGA
Tags: OV,Birdseed,ismpolish,NGC
Full name: TCGA,OV,Birdseed,ismpolish,NGC
Number of files: 1
Names: TCGA-23-1027
Path (to the first file): callData/TCGA,OV,Birdseed,ismpolish,NGC/GenomeWideSNP˙6
Total file size: 3.54 MB
RAM: 0.00MB

B Session information

This report was automatically generated using the R.rsp package.

- R version 2.10.0 Patched (2009-11-21 r50532), `i386-pc-mingw32`
- Locale: `LC_COLLATE=English_United States.1252`, `LC_CTYPE=English_United States.1252`, `LC_MONETARY=English_United States.1252`, `LC_NUMERIC=C`, `LC_TIME=English_United States.1252`
- Base packages: base, datasets, graphics, grDevices, methods, stats, utils
- Other packages: aroma.cn.eval 0.1.1, aroma.core 1.3.5, aroma.light 1.15.1, digest 0.4.1, MASS 7.3-3, matrixStats 0.1.8, R.cache 0.2.0, R.filesets 0.6.5, R.menu 0.0.5, R.methodsS3 1.1.0, R.oo 1.6.6, R.rsp 0.3.6, R.utils 1.2.4, RColorBrewer 1.0-2, xtable 1.5-5
- Loaded via a namespace (and not attached): affxparser 1.18.0
