# Supplementary material for: TumorBoost: Normalization of allele-specific tumor copy numbers from a single pair of tumor-normal genotyping microarrays
Source: BMC Bioinformatics. 2010 May 12;11:245. doi: 10.1186/1471-2105-11-245 (PMC2894037; doi:10.1186/1471-2105-11-245)
Supplement: Additional file 6 — Illumina Human1M-Duo data after BeadStudio,XY preprocessing (sample TCGA-23-1027; with confidence scores). Assessment of TumorBoost based on tumor/normal pair TCGA-23-1027 in the Illumina Human1M-Duo data set preprocessed with the BeadStudio,XY method using the SNPs with 90% highest confidence scores. [file 1471-2105-11-245-S6.PDF]

Supplementary Note: Assessment of TumorBoost based on tumor/normal pair TCGA-23-1027 in the Illumina Human1M-Duo data set TCGA,OV,BeadStudio,XY using the SNPs with 90% highest confidence scores

Henrik Bengtsson, Pierre Neuvial, Terence P. Speed

March 9, 2010

Contents

1 Introduction 3

2 Data set 3
2.1 Preprocessing methods 3
2.2 Stratification on genotype confidence scores 3
2.3 List of change points 3

3 Region: TCGA-23-1027:Chr2@108-140,cp=124+/-0.5,s=0/1 4
3.1 Decrease in Heterozygosity (DH) and total copy-number tracks 4
3.2 Allele B fraction density plots 5
3.3 ROC curves 5
3.4 (β_N, β_T) plots 6
3.5 Allele-specific copy number estimates 7

4 Region: TCGA-23-1027:Chr2@125.0-157.0,cp=141.0+/-0.5,s=1/3 8
4.1 Decrease in Heterozygosity (DH) and total copy-number tracks 8
4.2 Allele B fraction density plots 9
4.3 ROC curves 9
4.4 (β_N, β_T) plots 10
4.5 Allele-specific copy number estimates 11

5 Region: TCGA-23-1027:Chr10@80-109,cp=94+/-0.5,s=0/2 12
5.1 Decrease in Heterozygosity (DH) and total copy-number tracks 12
5.2 Allele B fraction density plots 13
5.3 ROC curves 13
5.4 (β_N, β_T) plots 14
5.5 Allele-specific copy number estimates 15

6 Region: TCGA-23-1027:Chr10@106.5-113.5,cp=110+/-0.5,s=2/3 16
6.1 Decrease in Heterozygosity (DH) and total copy-number tracks 16
6.2 Allele B fraction density plots 17
6.3 ROC curves 17
6.4 (β_N, β_T) plots 18
6.5 Allele-specific copy number estimates 19

7 Region: TCGA-23-1027:Chr2@55-75.0,cp=65.0+/-0.5,s=0/1 20
7.1 Decrease in Heterozygosity (DH) and total copy-number tracks 20
7.2 Allele B fraction density plots 21
7.3 ROC curves 21
7.4 (β_N, β_T) plots 22
7.5 Allele-specific copy number estimates 23

8 Bootstrap estimates of test statistics for all regions **24**

A Data files **26**
A.1 Total copy numbers 26
A.2 Allele B fractions 26
A.3 Genotype calls 26
A.4 Genotype confidence scores 26

B Session information **28**

1 Introduction

This report, which is automatically generated, assesses the performance of the TumorBoost method based on a few change points in a particular tumor/normal pair. For more details on the evaluation methods, see the main TumorBoost manuscript.

2 Data set

The evaluation is this report is based on the tumor/normal pair (01A,10A) for individual TCGA-23-1027 in the data set TCGA,OV,BeadStudio,XY.

2.1 Preprocessing methods

The data was generated on the Illumina Human1M-Duo chip type. Each array was preprocessed separately using Birdseed's "XY-normalization" method, which is principle is a single-array method that relies neither on reference samples nor prior estimates.

2.2 Stratification on genotype confidence scores

We focus on the SNPs in which we are the most confident that they are heterozygous: the evaluation will involve the 90% SNPs with highest genotype confidence scores.

2.3 List of change points

For this data set, we have selected a few regions for which one can safely assume that there exists a single copy number change point. By definition, each change point separates two sets of genomic loci such that the true Decrease in Heterozygosity (DH) is the same within one set of loci but differs between the two sets. These regions were selected visually. For each region we chose a large enough safety margin to make our evaluation independent of the uncertainty on the true location of the change point.

Chr	Region	Change point	Margin	Before	After
2	108-140	124	0.5	'normal' (1,1)	'gain' (1,2)
2	125-157	141	0.5	'gain' (1,2)	'copy neutral LOH' (0,2)
10	80-109	94	0.5	'normal' (1,1)	'deletion' (0,1)
10	106.5-113.5	110	0.5	'deletion' (0,1)	'copy neutral LOH' (0,2)
2	55-75	65	0.5	'normal' (1,1)	'gain' (1,2)

Table 1: Regions in TCGA-23-1027 used for the evaluation and that each contain a single changepoint. All positions and lengths are in units of Mb.

We next compare how well each of these change points is detected using the above preprocessed signals followed or not by TumorBoost normalization using the ROC analysis described in the main TumorBoost manuscript at the full resolution as well as smoothed resolution with bin sizes $h = \{1, 2, 4\}$. Specifically, we compare the following three methods: (1) **"raw"**: preprocessed signals without TumorBoost normalization. (2) **"TBN,NGC"**: preprocessed signals followed by TumorBoost normalization using NGC genotype calls. For completeness we also include an evaluation of Total copy numbers (TCN).

3 Region: TCGA-23-1027:Chr2@108-140,cp=124+/-0.5,s=0/1

3.1 Decrease in Heterozygosity (DH) and total copy-number tracks

(a) raw,NGC.

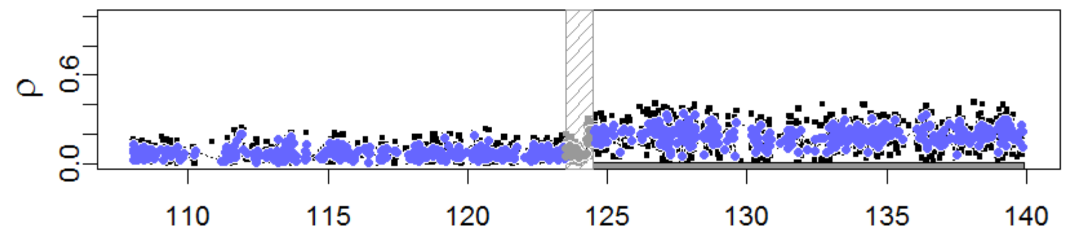

(b) TBN,NGC,NGC.

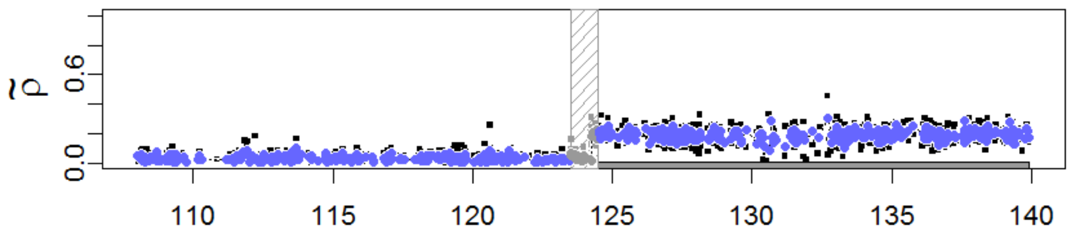

(c) TCN,NGC.

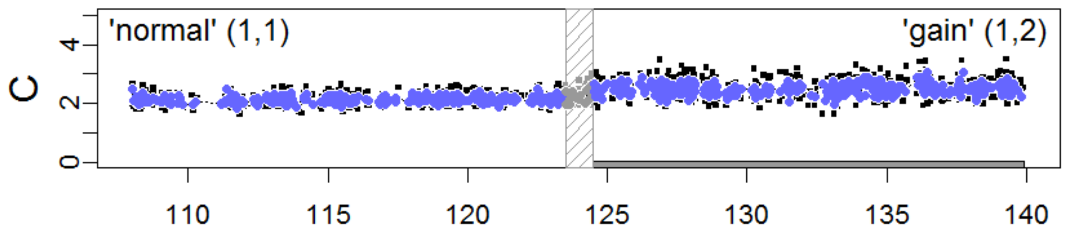

Figure 1: Decrease in Heterozygosity (DH) and total copy numbers for region TCGA-23-1027:Chr2@108-140,cp=124+/-0.5,s=0/1. Only heterozygous SNPs are plotted. There are 1454 loci of state 'normal' (1,1) ("negatives") and 1454 loci of state 'gain' (1,2) ("positives"), where the latter are highlighted with a solid bar beneath. In total 84 loci within the safety margin were excluded.

3.2 Allele B fraction density plots

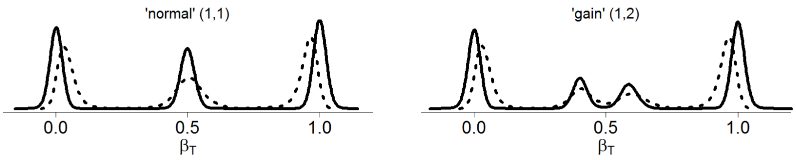

(a) 'normal' (1,1). (b) 'gain' (1,2).

Figure 2: Density of raw (dashed lines) and TumorBoost-normalized (solid lines) allele B fractions for region TCGA-23-1027:Chr2@108-140,cp=124+/-0.5,s=0/1.

3.3 ROC curves

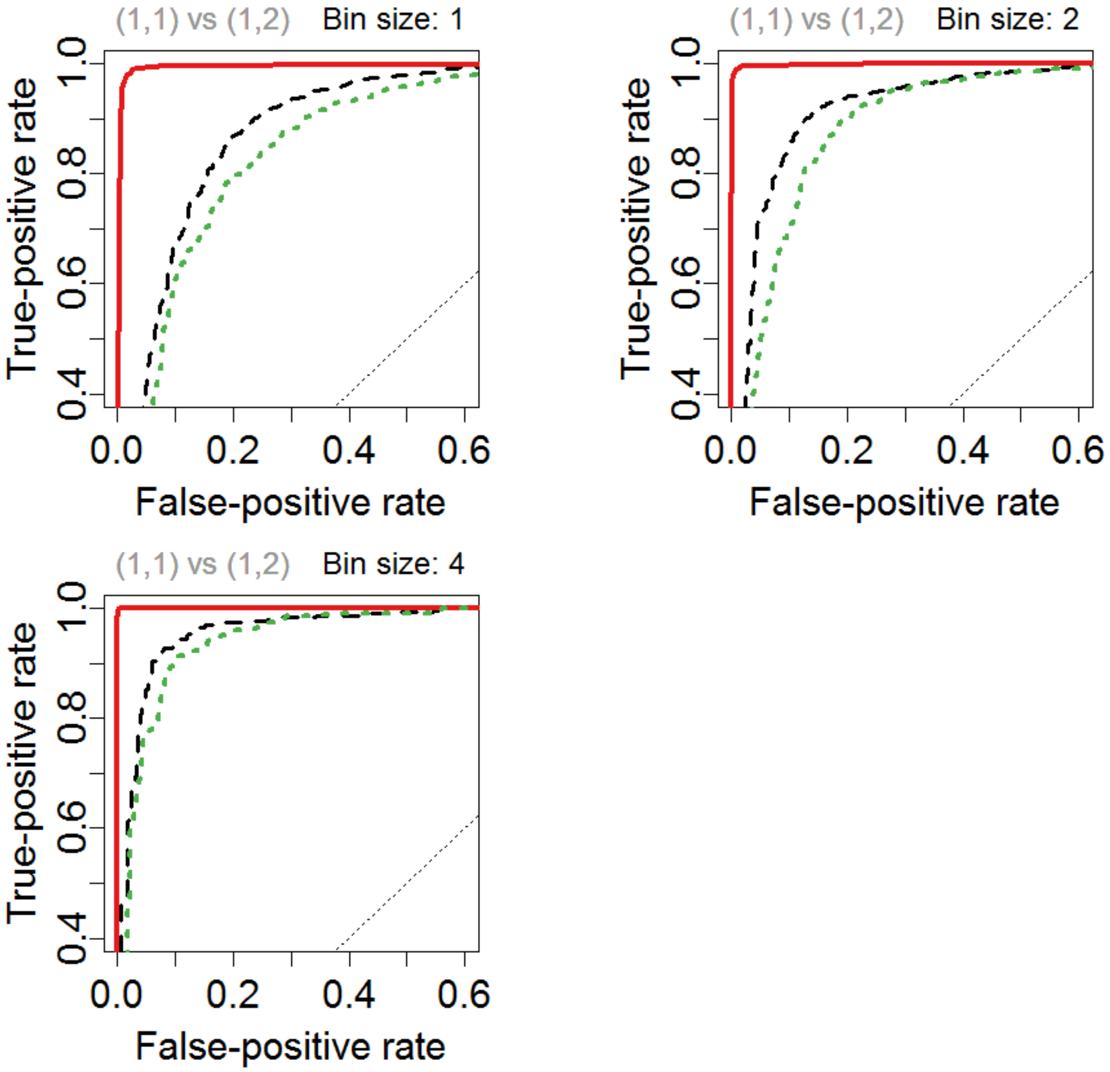

Figure 3: ROC curves for each preprocessing method at the full resolution as well as 2 different amounts of smoothing (using the mean() function) for region TCGA-23-1027:Chr2@108-140,cp=124+/-0.5,s=0/1. Legend: raw,NGC (dashed; #000000), TBN,NGC,NGC (solid; #E41A1C) and TCN,NGC (dotted; #4DAF4A).

3.4 (β_N, β_T) plots

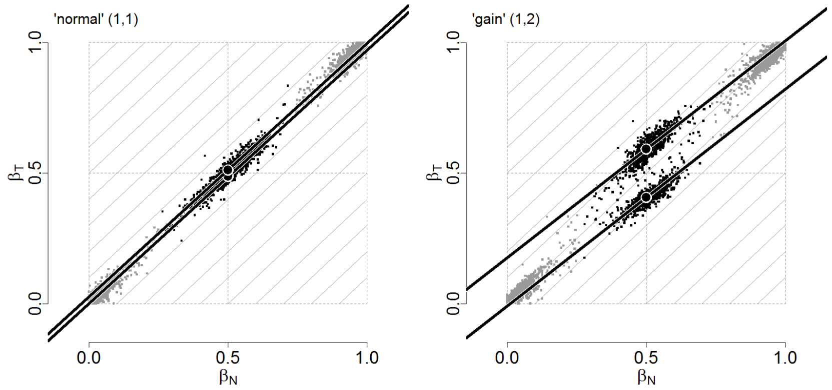

Figure 4: raw,NGC for region TCGA-23-1027:Chr2@108-140,cp=124+/-0.5,s=0/1.

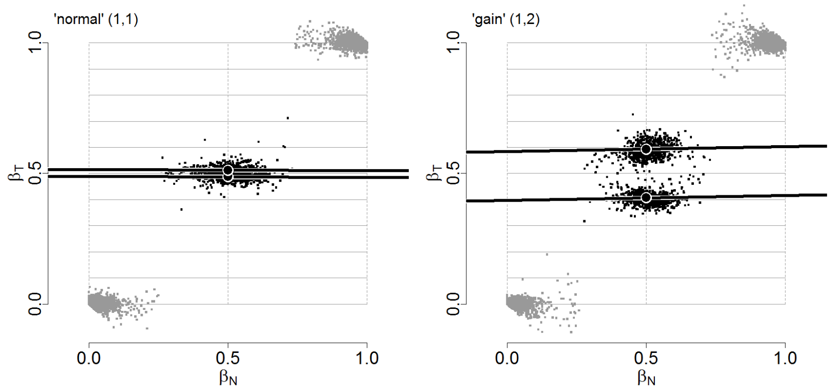

Figure 5: TBN,NGC,NGC for region TCGA-23-1027:Chr2@108-140,cp=124+/-0.5,s=0/1.

3.5 Allele-specific copy number estimates

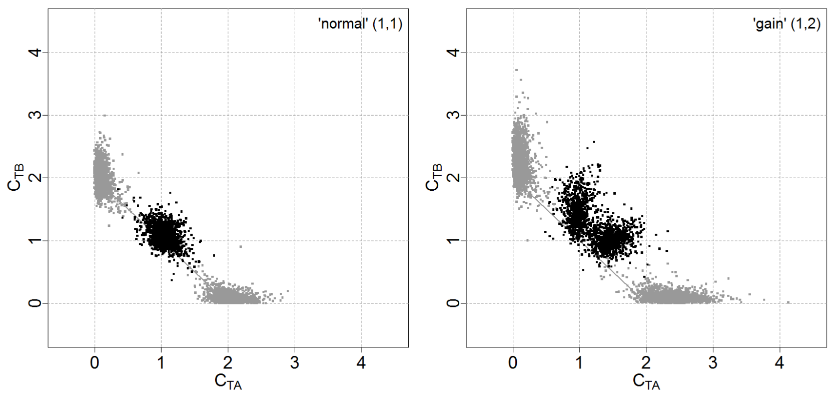

Figure 6: raw,NGC for region TCGA-23-1027:Chr2@108-140,cp=124+/-0.5,s=0/1.

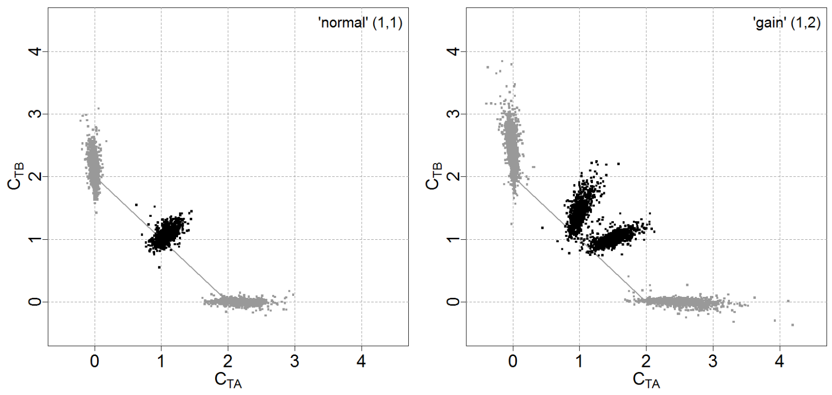

Figure 7: TBN,NGC,NGC for region TCGA-23-1027:Chr2@108-140,cp=124+/-0.5,s=0/1.

4 Region: TCGA-23-1027:Chr2@125.0-157.0,cp=141.0+/-0.5,s=1/3

4.1 Decrease in Heterozygosity (DH) and total copy-number tracks

(a) raw,NGC.

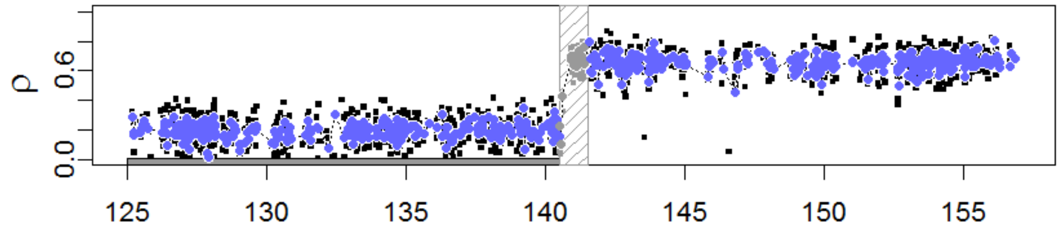

(b) TBN,NGC,NGC.

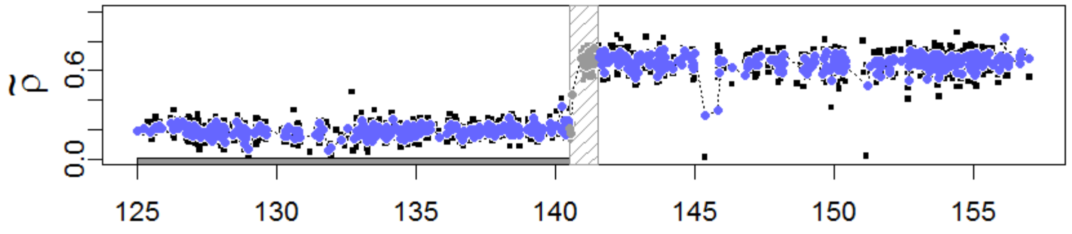

(c) TCN,NGC.

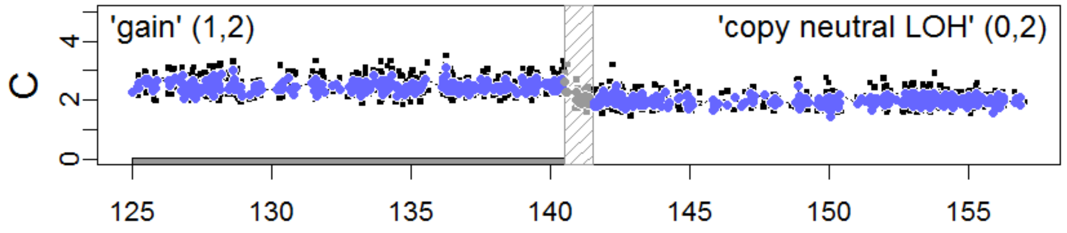

Figure 8: Decrease in Heterozygosity (DH) and total copy numbers for region TCGA-23-1027:Chr2@125.0-157.0,cp=141.0+/-0.5,s=1/3. Only heterozygous SNPs are plotted. There are 1171 loci of state 'gain' (1,2) ("negatives") and 1171 loci of state 'copy neutral LOH' (0,2) ("positives"), where the latter are highlighted with a solid bar beneath. In total 75 loci within the safety margin were excluded.

4.2 Allele B fraction density plots

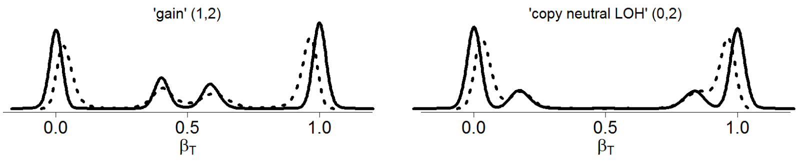

(a) 'gain' (1,2).

(b) 'copy neutral LOH' (0,2).

Figure 9: Density of raw (dashed lines) and TumorBoost-normalized (solid lines) allele B fractions for region TCGA-23-1027:Chr2@125.0-157.0,cp=141.0+/-0.5,s=1/3.

4.3 ROC curves

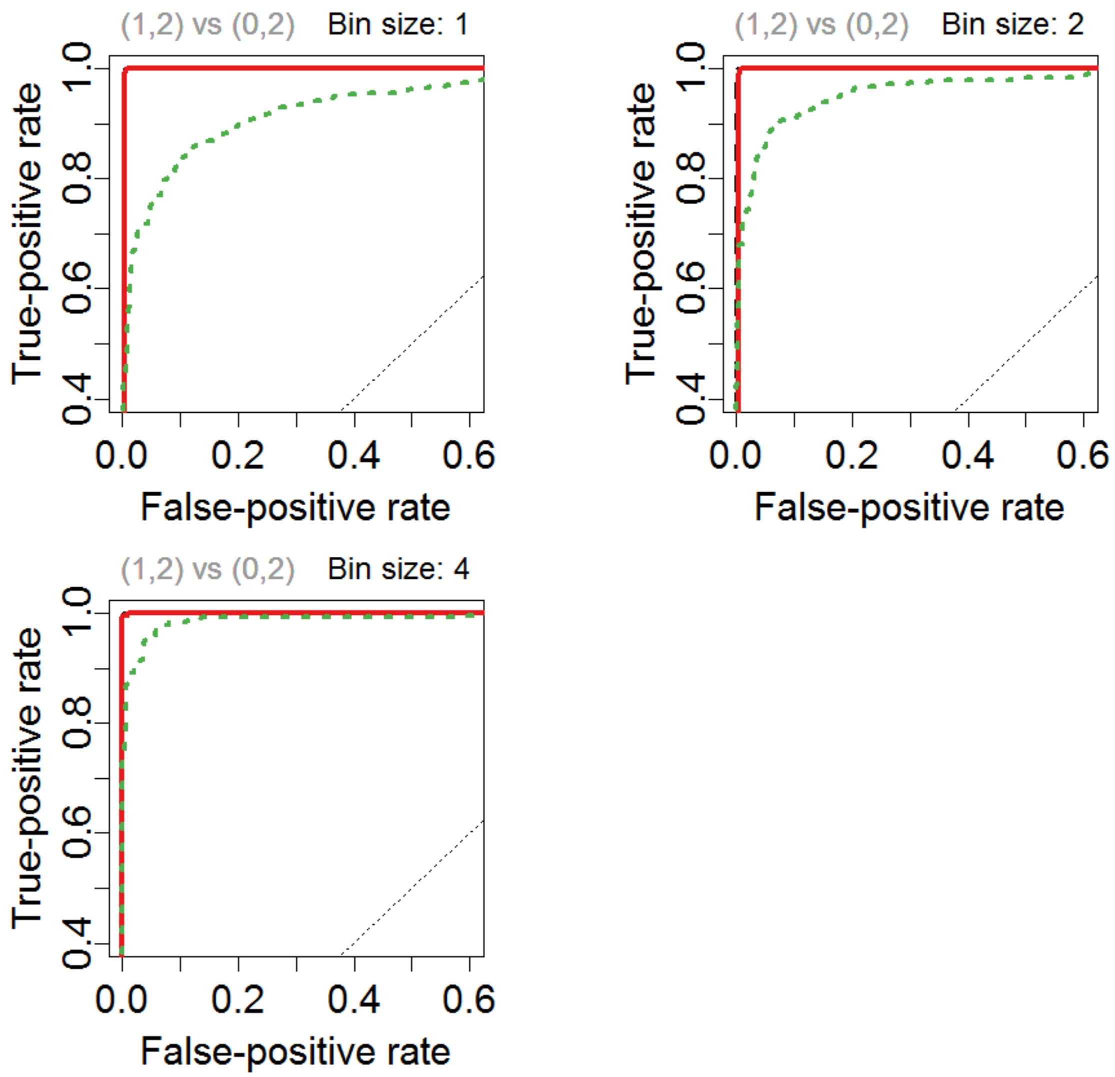

Figure 10: ROC curves for each preprocessing method at the full resolution as well as 2 different amounts of smoothing (using the mean() function) for region TCGA-23-1027:Chr2@125.0-157.0,cp=141.0+/-0.5,s=1/3. Legend: raw,NGC (dashed; #000000), TBN,NGC,NGC (solid; #E41A1C) and TCN,NGC (dotted; #4DAF4A).

4.4 (β_N, β_T) plots

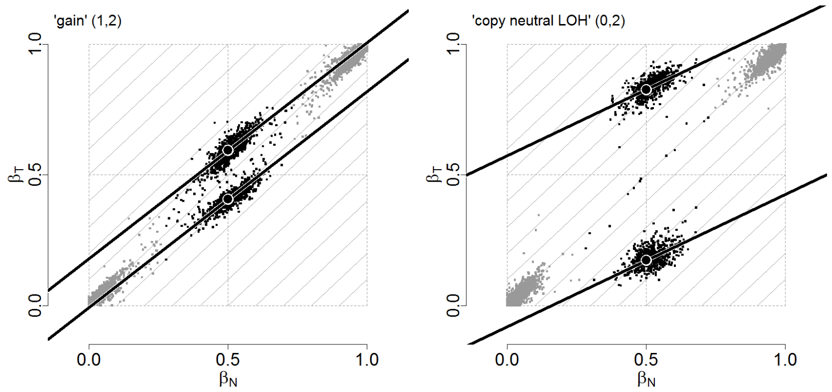

Figure 11: raw,NGC for region TCGA-23-1027:Chr2@125.0-157.0,cp=141.0+/-0.5,s=1/3.

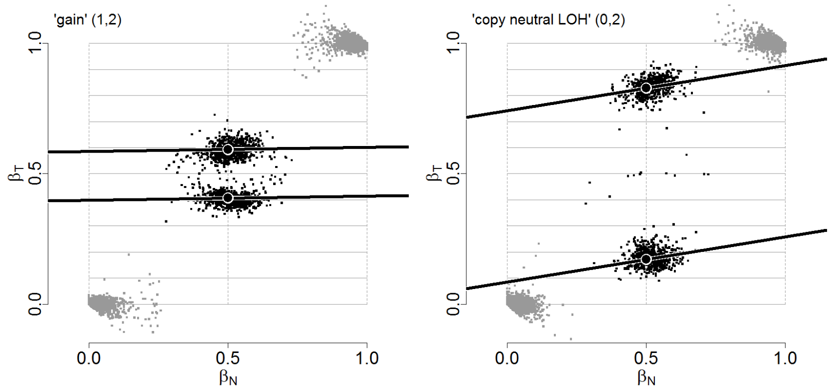

Figure 12: TBN,NGC,NGC for region TCGA-23-1027:Chr2@125.0-157.0,cp=141.0+/-0.5,s=1/3.

4.5 Allele-specific copy number estimates

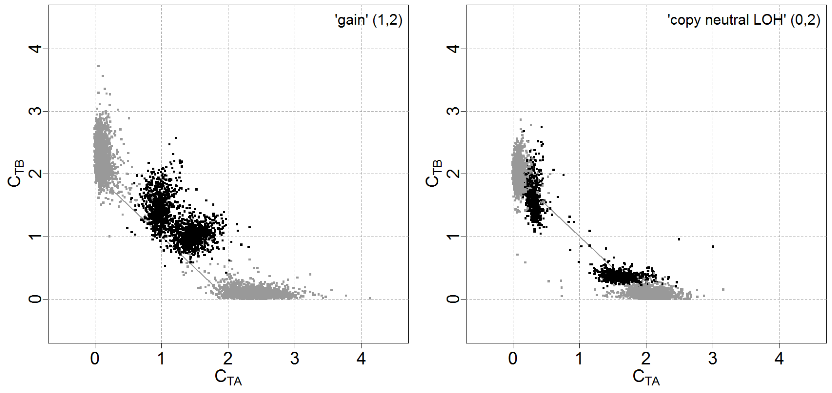

Figure 13: raw,NGC for region TCGA-23-1027:Chr2@125.0-157.0,cp=141.0+/-0.5,s=1/3.

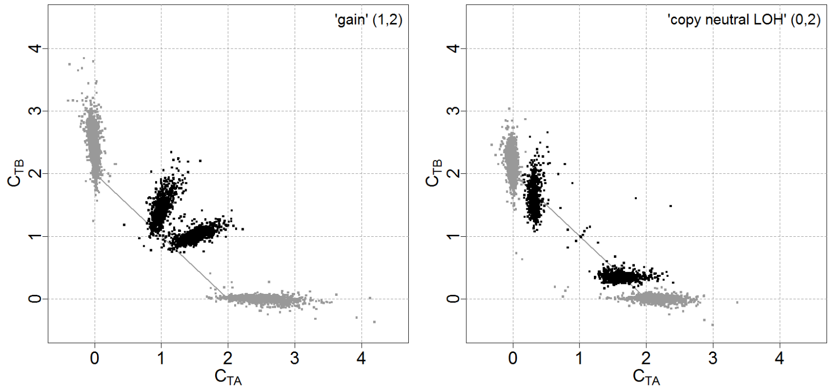

Figure 14: TBN,NGC,NGC for region TCGA-23-1027:Chr2@125.0-157.0,cp=141.0+/-0.5,s=1/3.

5 Region: TCGA-23-1027:Chr10@80-109,cp=94+/-0.5,s=0/2

5.1 Decrease in Heterozygosity (DH) and total copy-number tracks

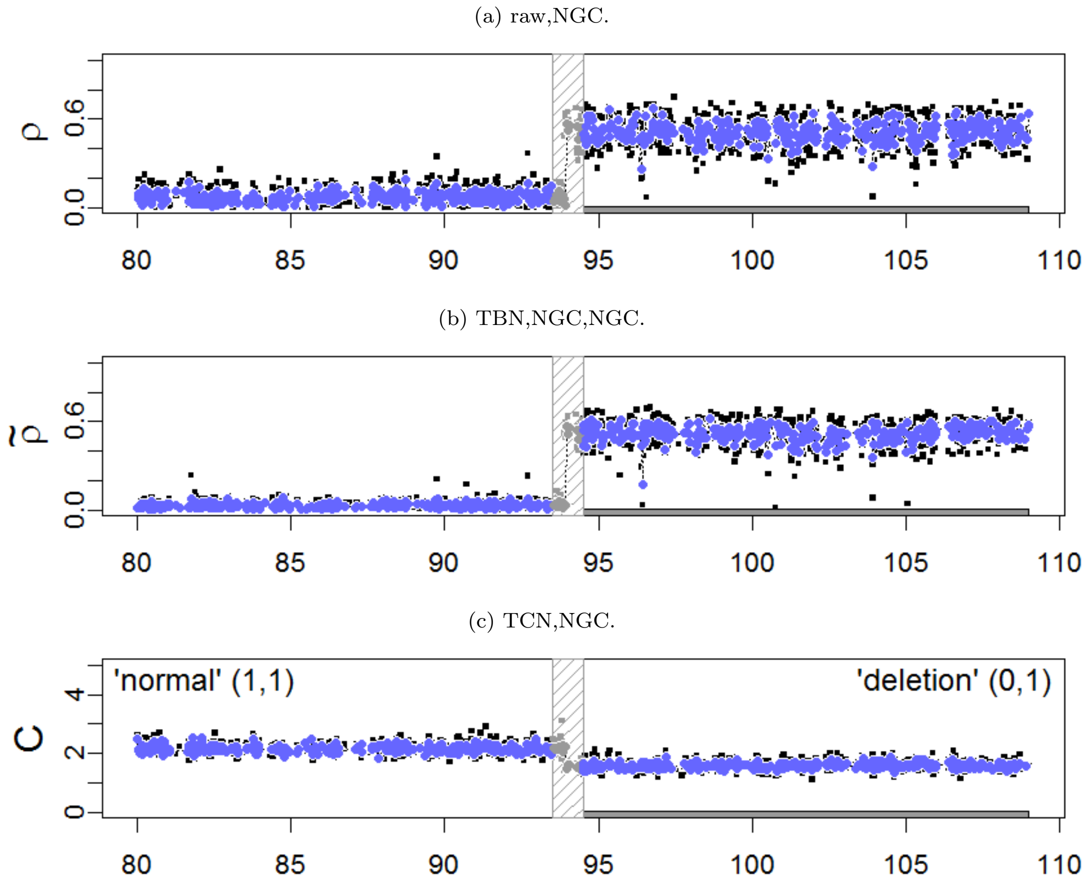

Figure 15: Decrease in Heterozygosity (DH) and total copy numbers for region TCGA-23-1027:Chr10@80-109,cp=94+/-0.5,s=0/2. Only heterozygous SNPs are plotted. There are 1410 loci of state 'normal' (1,1) ("negatives") and 1410 loci of state 'deletion' (0,1) ("positives"), where the latter are highlighted with a solid bar beneath. In total 90 loci within the safety margin were excluded.

5.2 Allele B fraction density plots

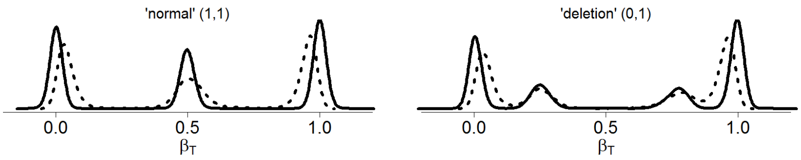

(a) 'normal' (1,1). (b) 'deletion' (0,1).

Figure 16: Density of raw (dashed lines) and TumorBoost-normalized (solid lines) allele B fractions for region TCGA-23-1027:Chr10@80-109,cp=94+/-0.5,s=0/2.

5.3 ROC curves

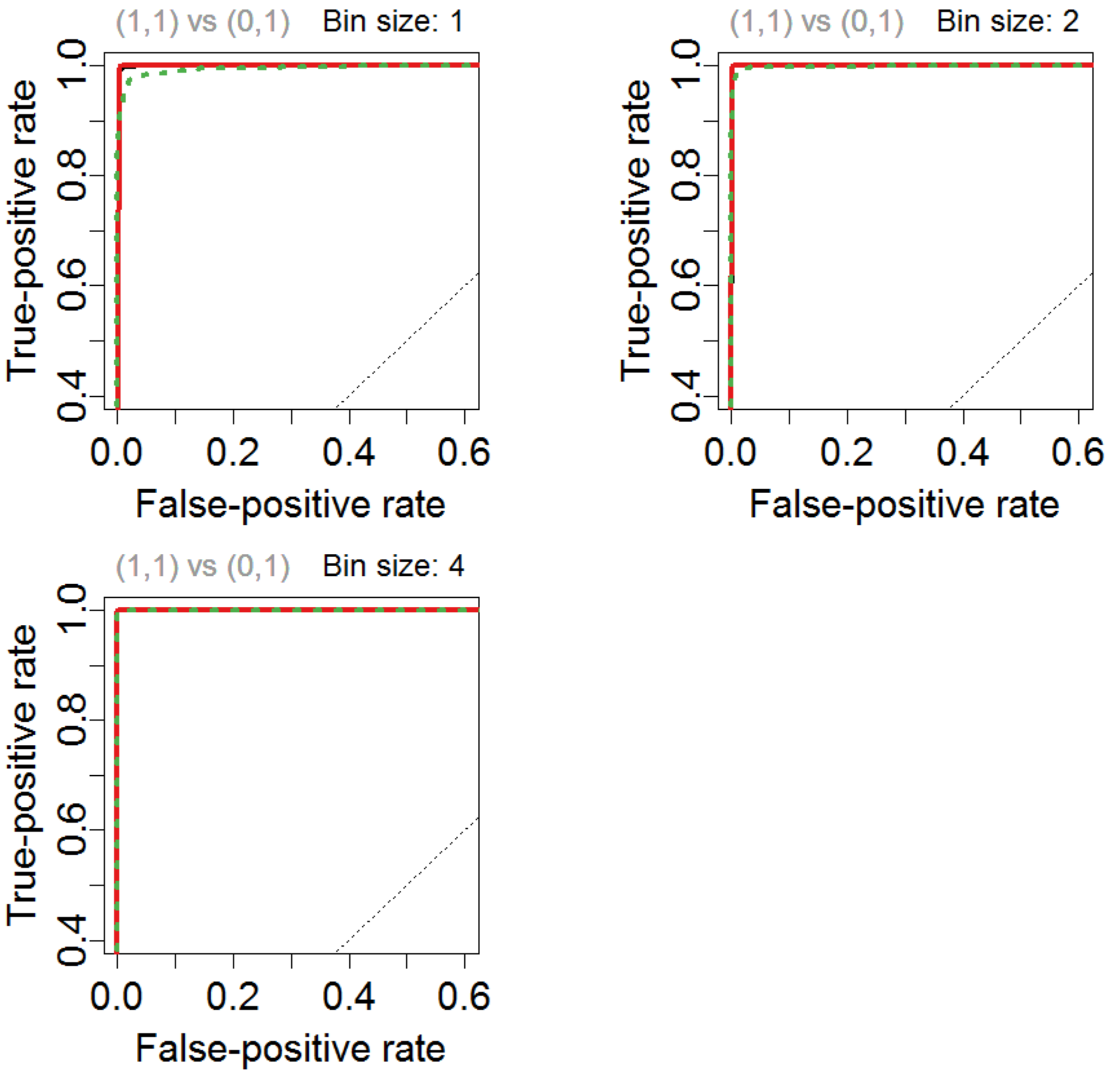

Figure 17: ROC curves for each preprocessing method at the full resolution as well as 2 different amounts of smoothing (using the mean() function) for region TCGA-23-1027:Chr10@80-109,cp=94+/-0.5,s=0/2. Legend: raw,NGC (dashed; #000000), TBN,NGC,NGC (solid; #E41A1C) and TCN,NGC (dotted; #4DAF4A).

5.4 (β_N, β_T) plots

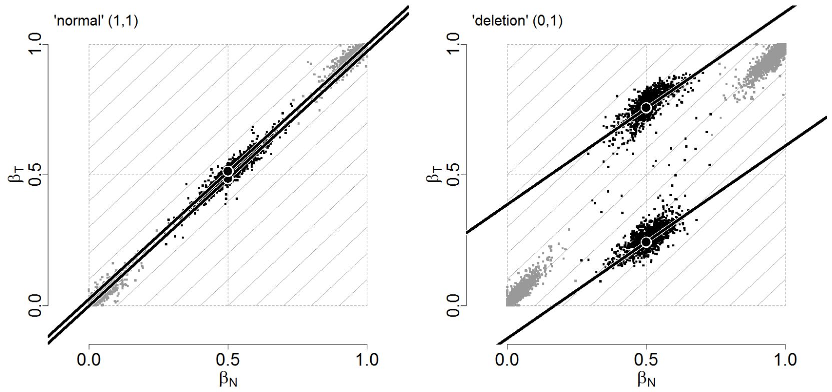

Figure 18: raw,NGC for region TCGA-23-1027:Chr10@80-109,cp=94+/-0.5,s=0/2.

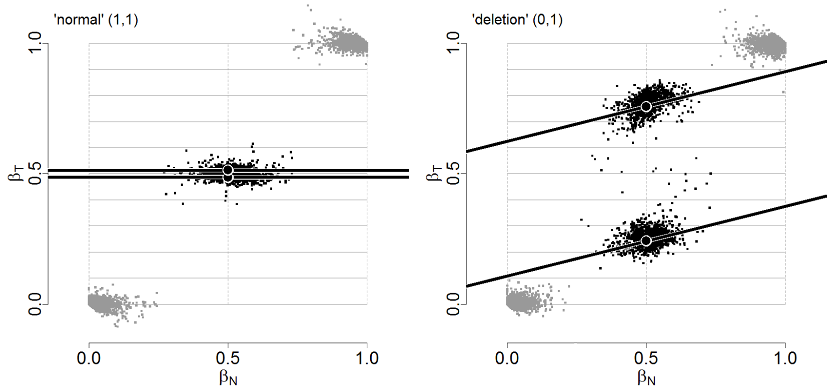

Figure 19: TBN,NGC,NGC for region TCGA-23-1027:Chr10@80-109,cp=94+/-0.5,s=0/2.

5.5 Allele-specific copy number estimates

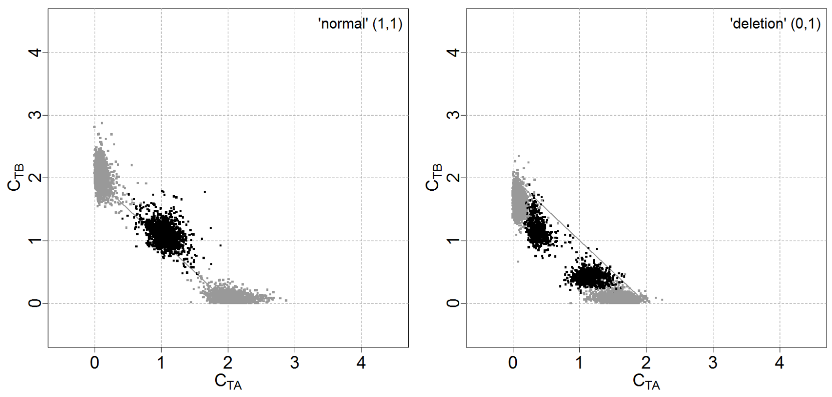

Figure 20: raw,NGC for region TCGA-23-1027:Chr10@80-109,cp=94+/-0.5,s=0/2.

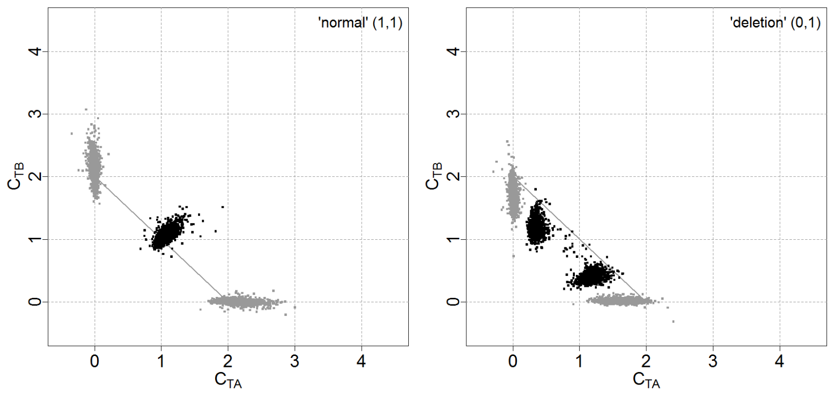

Figure 21: TBN,NGC,NGC for region TCGA-23-1027:Chr10@80-109,cp=94+/-0.5,s=0/2.

6 Region: TCGA-23-1027:Chr10@106.5-113.5,cp=110+/-0.5,s=2/3

6.1 Decrease in Heterozygosity (DH) and total copy-number tracks

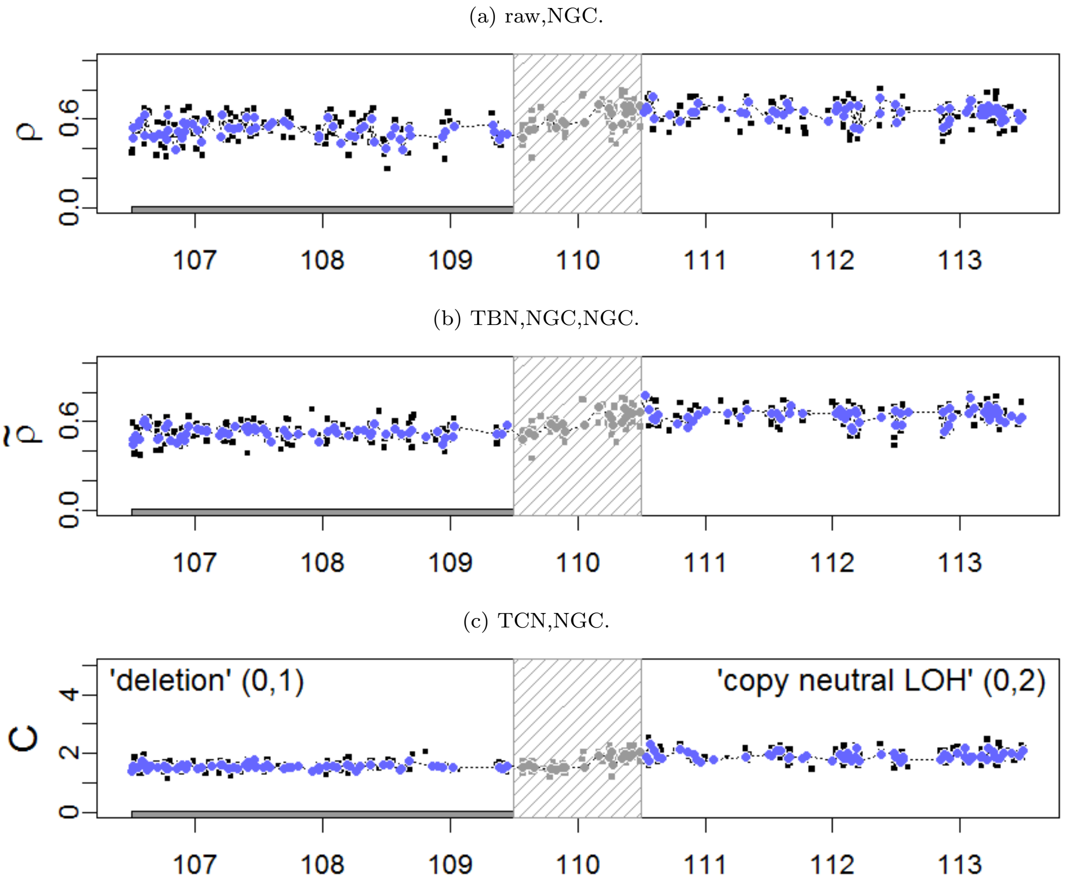

Figure 22: Decrease in Heterozygosity (DH) and total copy numbers for region TCGA-23-1027:Chr10@106.5-113.5,cp=110+/-0.5,s=2/3. Only heterozygous SNPs are plotted. There are 245 loci of state 'deletion' (0,1) ("negatives") and 245 loci of state 'copy neutral LOH' (0,2) ("positives"), where the latter are highlighted with a solid bar beneath. In total 73 loci within the safety margin were excluded.

6.2 Allele B fraction density plots

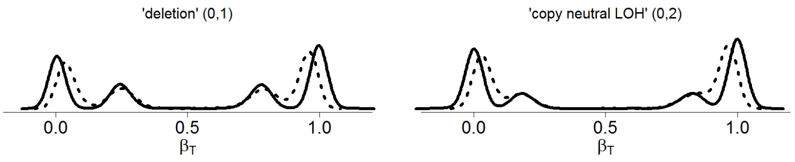

(a) 'deletion' (0,1). (b) 'copy neutral LOH' (0,2).

Figure 23: Density of raw (dashed lines) and TumorBoost-normalized (solid lines) allele B fractions for region TCGA-23-1027:Chr10@106.5-113.5,cp=110+/-0.5,s=2/3.

6.3 ROC curves

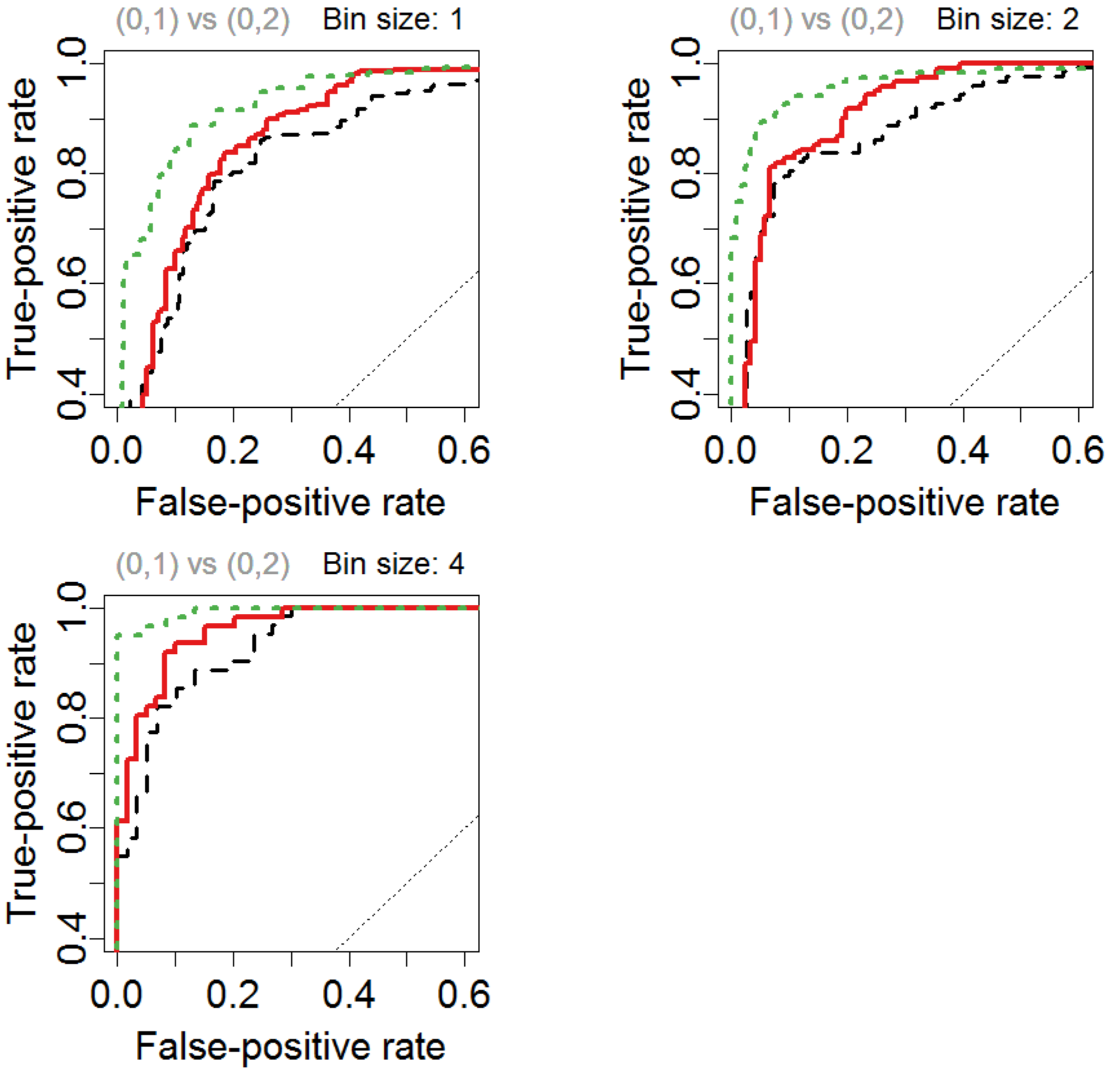

Figure 24: ROC curves for each preprocessing method at the full resolution as well as 2 different amounts of smoothing (using the mean() function) for region TCGA-23-1027:Chr10@106.5-113.5,cp=110+/-0.5,s=2/3. Legend: raw,NGC (dashed; #000000), TBN,NGC,NGC (solid; #E41A1C) and TCN,NGC (dotted; #4DAF4A).

6.4 (β_N, β_T) plots

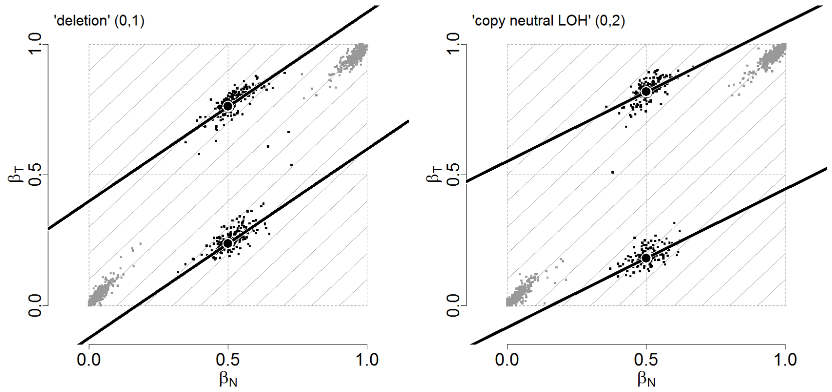

Figure 25: raw,NGC for region TCGA-23-1027:Chr10@106.5-113.5,cp=110+/-0.5,s=2/3.

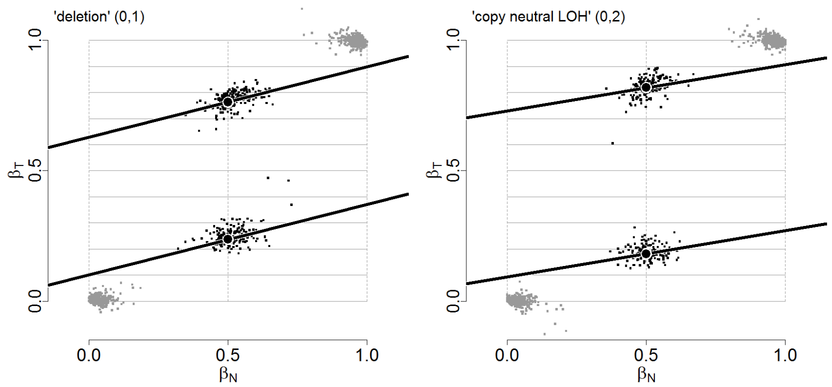

Figure 26: TBN,NGC,NGC for region TCGA-23-1027:Chr10@106.5-113.5,cp=110+/-0.5,s=2/3.

6.5 Allele-specific copy number estimates

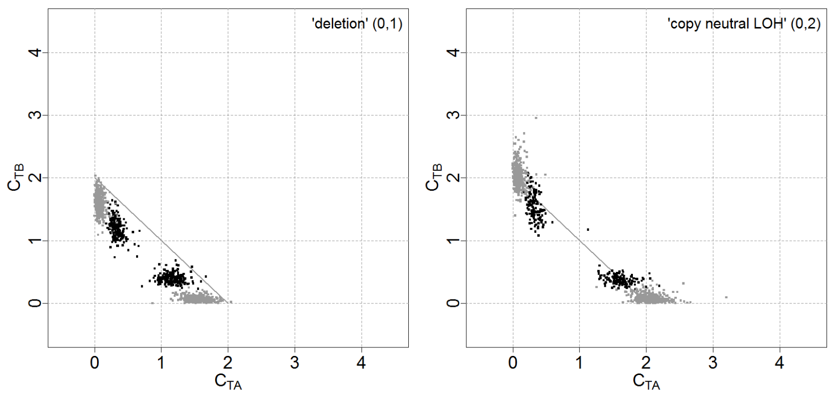

Figure 27: raw,NGC for region TCGA-23-1027:Chr10@106.5-113.5,cp=110+/-0.5,s=2/3.

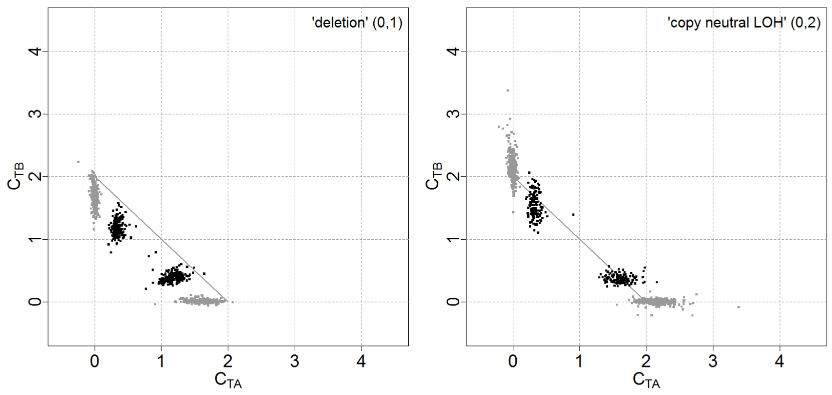

Figure 28: TBN,NGC,NGC for region TCGA-23-1027:Chr10@106.5-113.5,cp=110+/-0.5,s=2/3.

7 Region: TCGA-23-1027:Chr2@55-75.0,cp=65.0+/-0.5,s=0/1

7.1 Decrease in Heterozygosity (DH) and total copy-number tracks

(a) raw,NGC.

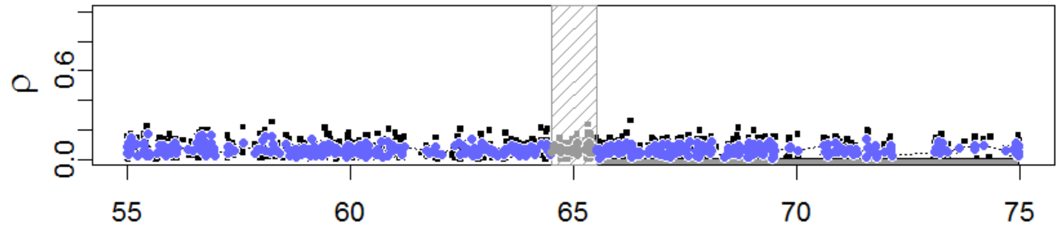

(b) TBN,NGC,NGC.

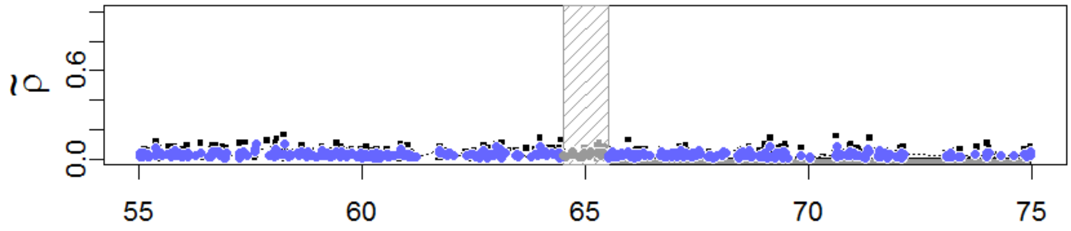

(c) TCN,NGC.

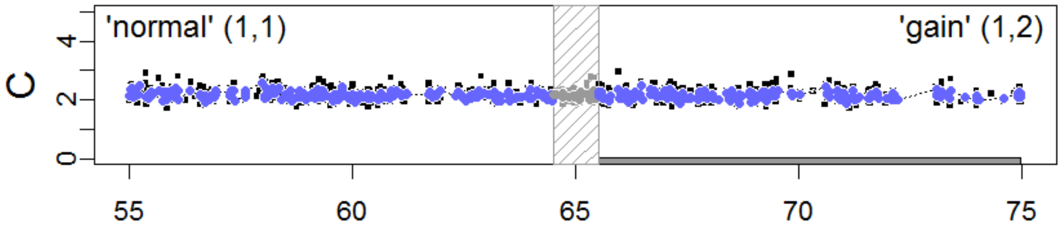

Figure 29: Decrease in Heterozygosity (DH) and total copy numbers for region TCGA-23-1027:Chr2@55-75.0,cp=65.0+/-0.5,s=0/1. Only heterozygous SNPs are plotted. There are 856 loci of state 'normal' (1,1) ("negatives") and 856 loci of state 'gain' (1,2) ("positives"), where the latter are highlighted with a solid bar beneath. In total 84 loci within the safety margin were excluded.

7.2 Allele B fraction density plots

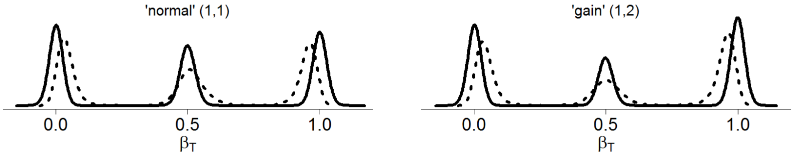

(a) 'normal' (1,1). (b) 'gain' (1,2).

Figure 30: Density of raw (dashed lines) and TumorBoost-normalized (solid lines) allele B fractions for region TCGA-23-1027:Chr2@55-75.0,cp=65.0+/-0.5,s=0/1.

7.3 ROC curves

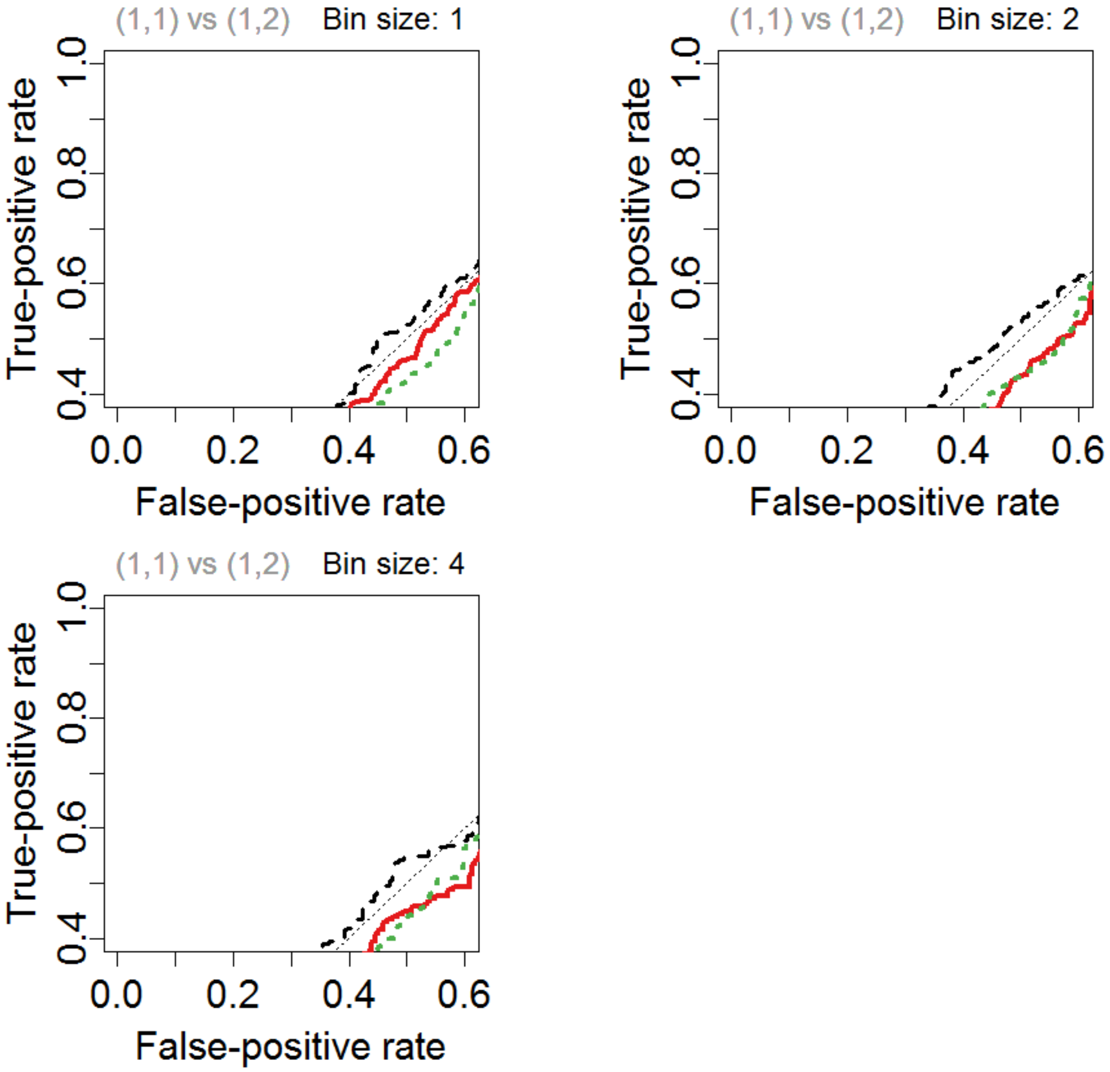

Figure 31: ROC curves for each preprocessing method at the full resolution as well as 2 different amounts of smoothing (using the mean() function) for region TCGA-23-1027:Chr2@55-75.0,cp=65.0+/-0.5,s=0/1. Legend: raw,NGC (dashed; #000000), TBN,NGC,NGC (solid; #E41A1C) and TCN,NGC (dotted; #4DAF4A).

7.4 (β_N, β_T) plots

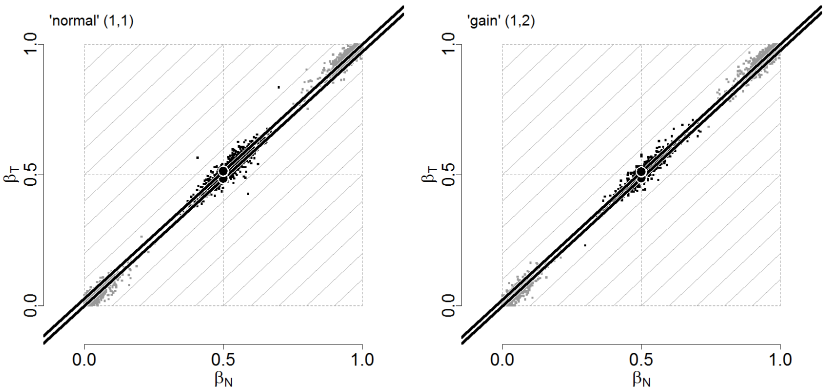

Figure 32: raw,NGC for region TCGA-23-1027:Chr2@55-75.0,cp=65.0+/-0.5,s=0/1.

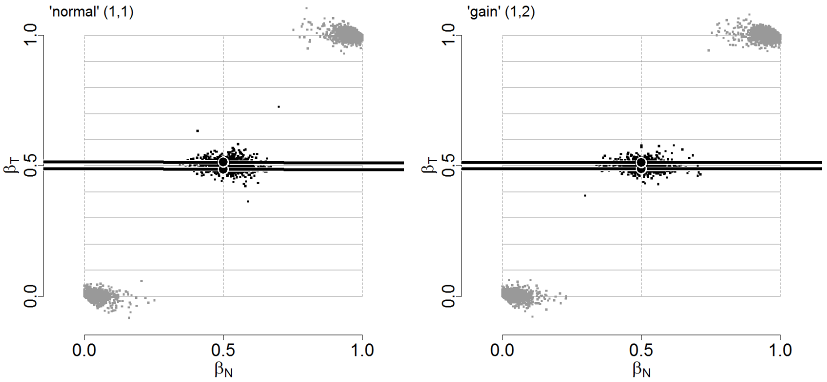

Figure 33: TBN,NGC,NGC for region TCGA-23-1027:Chr2@55-75.0,cp=65.0+/-0.5,s=0/1.

7.5 Allele-specific copy number estimates

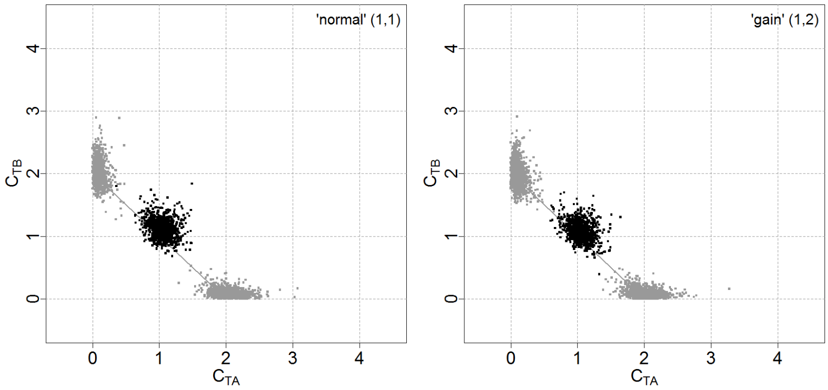

Figure 34: raw,NGC for region TCGA-23-1027:Chr2@55-75.0,cp=65.0+/-0.5,s=0/1.

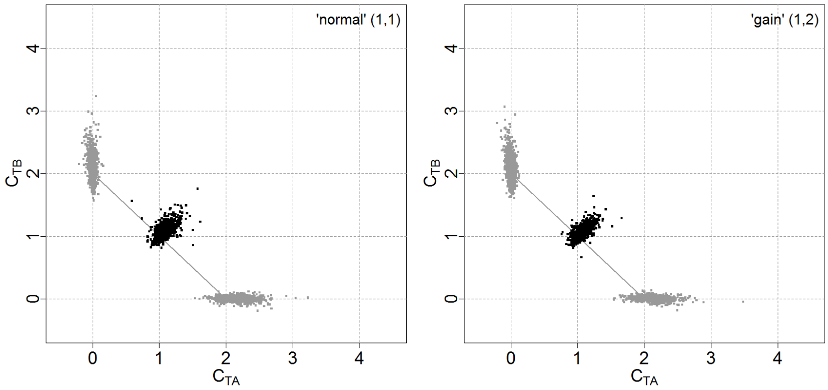

Figure 35: TBN,NGC,NGC for region TCGA-23-1027:Chr2@55-75.0,cp=65.0+/-0.5,s=0/1.

8 Bootstrap estimates of test statistics for all regions

	0/1	1/3	0/2	2/3	0/1
raw,NGC	19.393±1.368	62.039±4.003	62.585±3.126	15.148±1.167	0.858±0.583
TBN,NGC,NGC	45.334±2.773	80.384±8.959	92.095±7.289	18.561±1.127	1.247±0.893
TCN,NGC	16.285±0.981	22.030±1.262	38.780±2.039	24.520±1.420	1.935±1.046

Table 2: Student test statistics of the null hypothesis of equal mean before and after each PCN change point (heterozygous SNPs): raw or TumorBoost-normalized DH, and total copy number (last line). Mean ± standard deviation across 100 samplings of 225 points (for each PCN state) from the original data set. The larger value, the more different the true means are.

References

A Data files

A.1 Total copy numbers

$‘TCGA,OV,BeadStudio,XY‘
AromaUnitTotalCnBinarySet:
Name: TCGA
Tags: OV,BeadStudio,XY
Full name: TCGA,OV,BeadStudio,XY
Number of files: 1
Names: TCGA-23-1027
Path (to the first file): rawCnData/TCGA,OV,BeadStudio,XY/Human1M-Duo
Total file size: 4.58 MB
RAM: 0.00MB

A.2 Allele B fractions

$raw
AromaUnitFracBCnBinarySet:
Name: TCGA
Tags: OV,BeadStudio,XY
Full name: TCGA,OV,BeadStudio,XY
Number of files: 1
Names: TCGA-23-1027
Path (to the first file): totalAndFracBData/TCGA,OV,BeadStudio,XY/Human1M-Duo
Total file size: 4.58 MB
RAM: 0.00MB

$‘TBN,NGC‘
AromaUnitFracBCnBinarySet:
Name: TCGA
Tags: OV,BeadStudio,XY,TBN,NGC
Full name: TCGA,OV,BeadStudio,XY,TBN,NGC
Number of files: 1
Names: TCGA-23-1027
Path (to the first file): totalAndFracBData/TCGA,OV,BeadStudio,XY,TBN,NGC/Human1M-Duo
Total file size: 4.58 MB
RAM: 0.00MB

A.3 Genotype calls

$NGC
AromaUnitGenotypeCallSet:
Name: TCGA
Tags: OV,BeadStudio,XY,NGC
Full name: TCGA,OV,BeadStudio,XY,NGC
Number of files: 1
Names: TCGA-23-1027
Path (to the first file): callData/TCGA,OV,BeadStudio,XY,NGC/Human1M-Duo
Total file size: 2.29 MB
RAM: 0.00MB

A.4 Genotype confidence scores

$NGC
AromaUnitSignalBinarySet:

Name: TCGA
Tags: OV,BeadStudio,XY,NGC
Full name: TCGA,OV,BeadStudio,XY,NGC
Number of files: 1
Names: TCGA-23-1027
Path (to the first file): callData/TCGA,OV,BeadStudio,XY,NGC/Human1M-Duo
Total file size: 4.57 MB
RAM: 0.00MB

B Session information

This report was automatically generated using the R.rsp package.

- R version 2.10.0 Patched (2009-11-21 r50532), `i386-pc-mingw32`
- Locale: `LC_COLLATE=English_United States.1252`, `LC_CTYPE=English_United States.1252`, `LC_MONETARY=English_United States.1252`, `LC_NUMERIC=C`, `LC_TIME=English_United States.1252`
- Base packages: base, datasets, graphics, grDevices, methods, stats, utils
- Other packages: aroma.cn.eval 0.1.1, aroma.core 1.3.5, aroma.light 1.15.1, digest 0.4.1, MASS 7.3-3, matrixStats 0.1.8, R.cache 0.2.0, R.filesets 0.6.5, R.menu 0.0.5, R.methodsS3 1.1.0, R.oo 1.6.6, R.rsp 0.3.6, R.utils 1.2.4, RColorBrewer 1.0-2, xtable 1.5-5
- Loaded via a namespace (and not attached): affxparser 1.18.0
