# Supplementary material for: TumorBoost: Normalization of allele-specific tumor copy numbers from a single pair of tumor-normal genotyping microarrays
Source: BMC Bioinformatics. 2010 May 12;11:245. doi: 10.1186/1471-2105-11-245 (PMC2894037; doi:10.1186/1471-2105-11-245)
Supplement: Additional file 10 — Affymetrix GenomeWideSNP_6 data after CRMAv2 preprocessing (sample TCGA-02-0001; with confidence scores). Assessment of TumorBoost based on tumor/normal pair TCGA-02-0001 in the Affymetrix GenomeWideSNP_6 data set preprocessed with the CRMAv2 method using the SNPs with 90% highest confidence scores. [file 1471-2105-11-245-S10.PDF]

Supplementary Note: Assessment of TumorBoost based on tumor/normal pair TCGA-02-0001 in the Affymetrix GenomeWideSNP_6 data set TCGA,GBM,CRMAv2 using the SNPs with 90% highest confidence scores

Henrik Bengtsson, Pierre Neuvial, Terence P. Speed

March 5, 2010

Contents

1 Introduction **3**

2 Data set **3**

2.1 Preprocessing methods . . . 3
2.2 Stratification on genotype confidence scores . . . 3
2.3 List of change points . . . 3

3 Region: TCGA-02-0001:Chr2@35-74,cp=57+/-1,s=1/2 **4**

3.1 Decrease in Heterozygosity (DH) and total copy-number tracks . . . 4
3.2 Allele B fraction density plots . . . 5
3.3 ROC curves . . . 5
3.4 (β_N, β_T) plots . . . 6
3.5 Allele-specific copy number estimates . . . 7

4 Region: TCGA-02-0001:Chr2@75-110,cp=96+/-1,s=1/4 **8**

4.1 Decrease in Heterozygosity (DH) and total copy-number tracks . . . 8
4.2 Allele B fraction density plots . . . 9
4.3 ROC curves . . . 9
4.4 (β_N, β_T) plots . . . 10
4.5 Allele-specific copy number estimates . . . 11

5 Region: TCGA-02-0001:Chr2@100-130,cp=110+/-1,s=4/0 **12**

5.1 Decrease in Heterozygosity (DH) and total copy-number tracks . . . 12
5.2 Allele B fraction density plots . . . 13
5.3 ROC curves . . . 13
5.4 (β_N, β_T) plots . . . 14
5.5 Allele-specific copy number estimates . . . 15

6 Region: TCGA-02-0001:Chr13@0-70,cp=45+/-1,s=0/2 **16**

6.1 Decrease in Heterozygosity (DH) and total copy-number tracks . . . 16
6.2 Allele B fraction density plots . . . 17
6.3 ROC curves . . . 17
6.4 (β_N, β_T) plots . . . 18
6.5 Allele-specific copy number estimates . . . 19

7 Bootstrap estimates of test statistics for all regions **20**

A Data files 22
A.1 Total copy numbers . . . 22
A.2 Allele B fractions . . . 22
A.3 Genotype calls . . . 22
A.4 Genotype confidence scores . . . 22

B Session information 24

1 Introduction

This report, which is automatically generated, assesses the performance of the TumorBoost method based on a few change points in a particular tumor/normal pair. For more details on the evaluation methods, see the main TumorBoost manuscript.

2 Data set

The evaluation is this report is based on the tumor/normal pair (01C,10A) for individual TCGA-02-0001 in the data set TCGA,GBM,CRMAv2.

2.1 Preprocessing methods

The data was generated on the Affymetrix GenomeWideSNP_6 chip type.

There is one CEL file per hybridization. Each CEL file was preprocessed separately using CR-MAv2 Bengtsson *et al.* (2009), without relying neither on reference samples nor prior estimates.

2.2 Stratification on genotype confidence scores

We focus on the SNPs in which we are the most confident that they are heterozygous: the evaluation will involve the 90% SNPs with highest genotype confidence scores.

2.3 List of change points

For this data set, we have selected a few regions for which one can safely assume that there exists a single copy number change point. By definition, each change point separates two sets of genomic loci such that the true Decrease in Heterozygosity (DH) is the same within one set of loci but differs between the two sets. These regions were selected visually. For each region we chose a large enough safety margin to make our evaluation independent of the uncertainty on the true location of the change point.

Chr	Region	Change point	Margin	Before	After
2	35-74	57	1	gain (1,2)	deletion (0,1)
2	75-110	96	1	gain (1,2)	gain (1,3)
2	100-130	110	1	gain (1,3)	normal (1,1)
13	0-70	45	1	normal (1,1)	deletion (0,1)

Table 1: Regions in TCGA-02-0001 used for the evaluation and that each contain a single changepoint. All positions and lengths are in units of Mb.

We next compare how well each of these change points is detected using the above preprocessed signals followed or not by TumorBoost normalization using the ROC analysis described in the main TumorBoost manuscript at the full resolution as well as smoothed resolution with bin sizes $h = \{1, 2, 4\}$. Specifically, we compare the following three methods: (1) **"raw"**: preprocessed signals without Tumor-Boost normalization. (2) **"TBN,NGC"**: preprocessed signals followed by TumorBoost normalization using NGC genotype calls. For completeness we also include an evaluation of Total copy numbers (TCN).

3 Region: TCGA-02-0001:Chr2@35-74,cp=57+/-1,s=1/2

3.1 Decrease in Heterozygosity (DH) and total copy-number tracks

(a) raw,NGC.

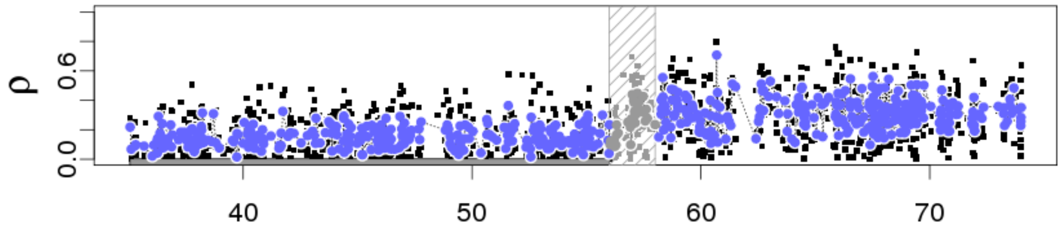

(b) TBN,NGC,NGC.

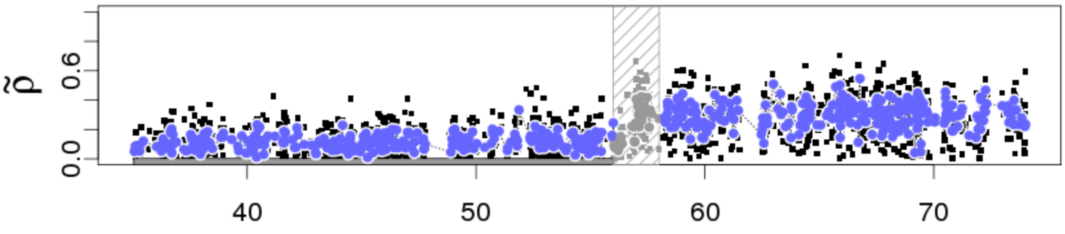

(c) TCN,NGC.

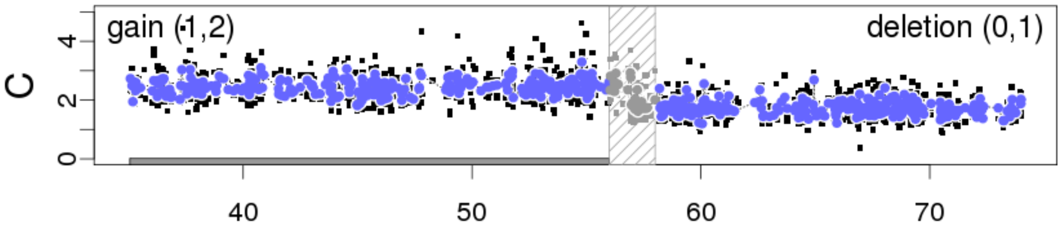

Figure 1: Decrease in Heterozygosity (DH) and total copy numbers for region TCGA-02-0001:Chr2@35-74,cp=57+/-1,s=1/2. Only heterozygous SNPs are plotted. There are 1093 loci of state gain (1,2) ("negatives") and 1093 loci of state deletion (0,1) ("positives"), where the latter are highlighted with a solid bar beneath. In total 120 loci within the safety margin were excluded.

3.2 Allele B fraction density plots

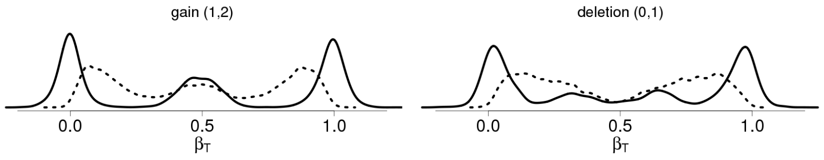

(a) gain (1,2). (b) deletion (0,1).

Figure 2: Density of raw (dashed lines) and TumorBoost-normalized (solid lines) allele B fractions for region TCGA-02-0001:Chr2@35-74,cp=57+/-1,s=1/2.

3.3 ROC curves

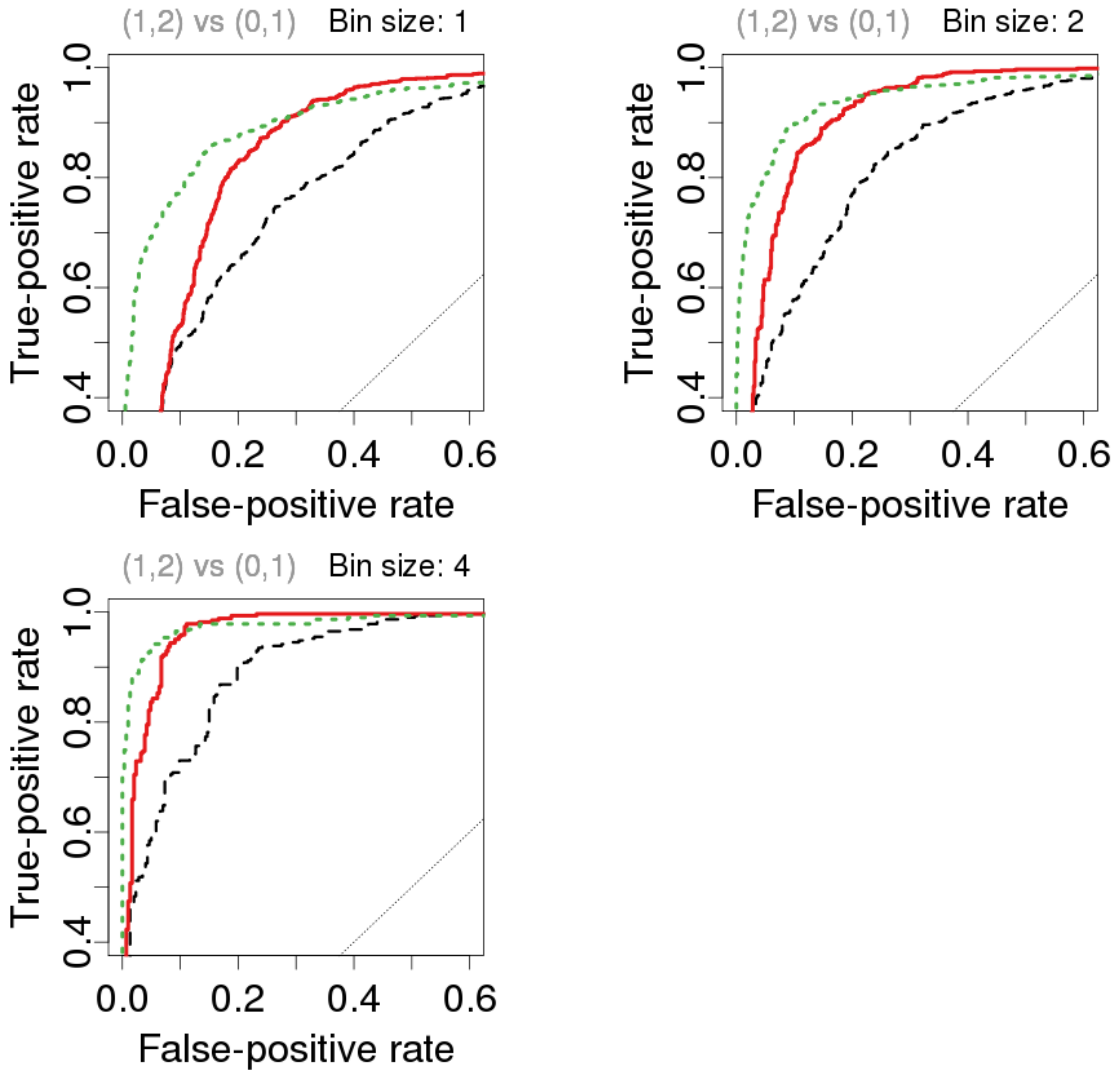

Figure 3: ROC curves for each preprocessing method at the full resolution as well as 2 different amounts of smoothing (using the mean() function) for region TCGA-02-0001:Chr2@35-74,cp=57+/-1,s=1/2. Legend: raw,NGC (dashed; #000000), TBN,NGC,NGC (solid; #E41A1C) and TCN,NGC (dotted; #4DAF4A).

3.4 (β_N, β_T) plots

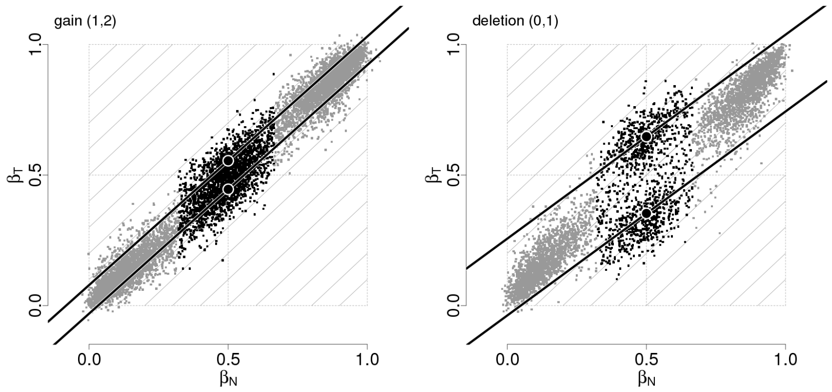

Figure 4: raw,NGC for region TCGA-02-0001:Chr2@35-74,cp=57+/-1,s=1/2.

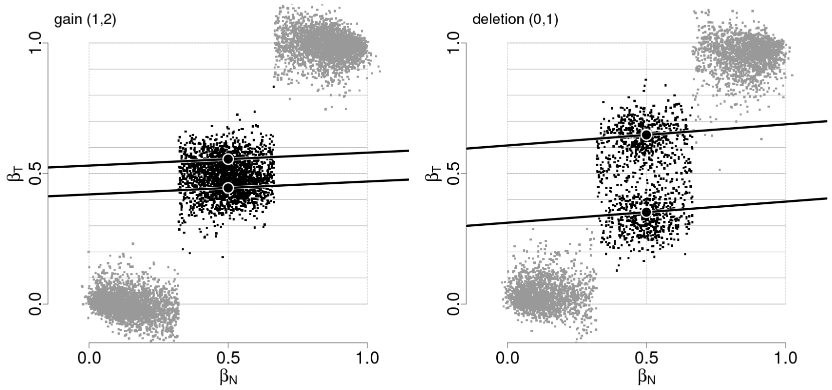

Figure 5: TBN,NGC,NGC for region TCGA-02-0001:Chr2@35-74,cp=57+/-1,s=1/2.

3.5 Allele-specific copy number estimates

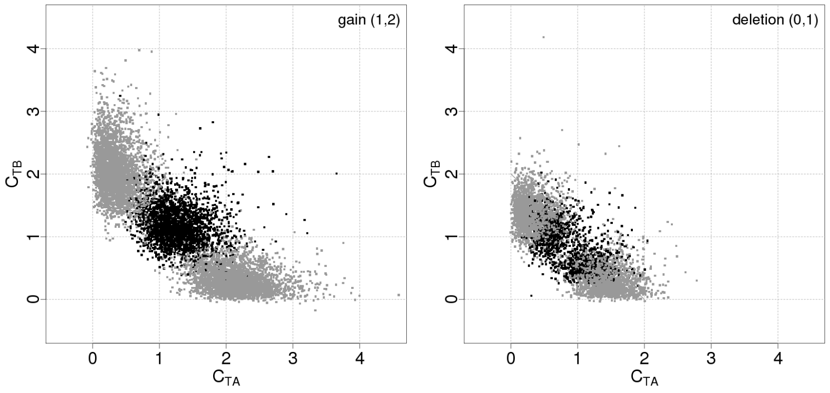

Figure 6: raw,NGC for region TCGA-02-0001:Chr2@35-74,cp=57+/-1,s=1/2.

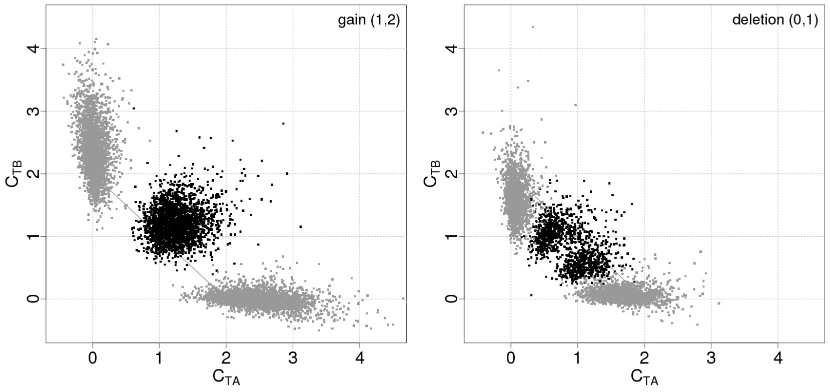

Figure 7: TBN,NGC,NGC for region TCGA-02-0001:Chr2@35-74,cp=57+/-1,s=1/2.

4 Region: TCGA-02-0001:Chr2@75-110,cp=96+/-1,s=1/4

4.1 Decrease in Heterozygosity (DH) and total copy-number tracks

(a) raw,NGC.

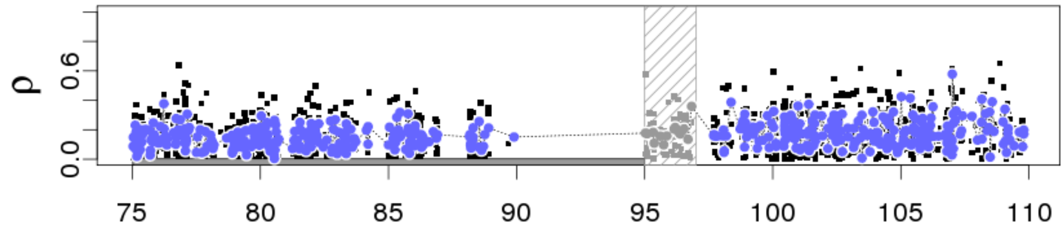

(b) TBN,NGC,NGC.

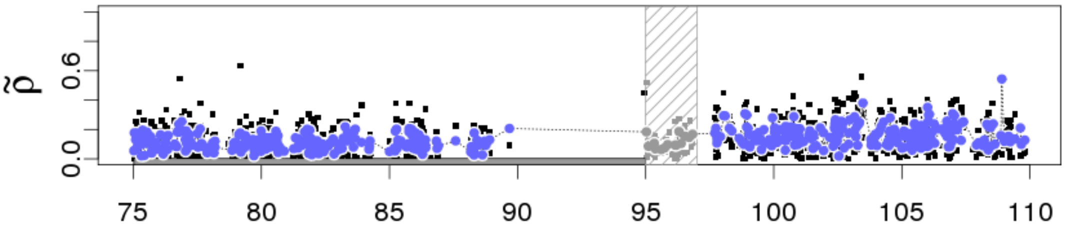

(c) TCN,NGC.

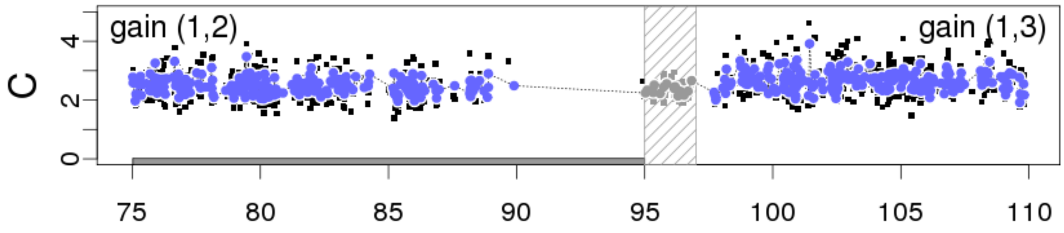

Figure 8: Decrease in Heterozygosity (DH) and total copy numbers for region TCGA-02-0001:Chr2@75-110,cp=96+/-1,s=1/4. Only heterozygous SNPs are plotted. There are 928 loci of state gain (1,2) ("negatives") and 928 loci of state gain (1,3) ("positives"), where the latter are highlighted with a solid bar beneath. In total 53 loci within the safety margin were excluded.

4.2 Allele B fraction density plots

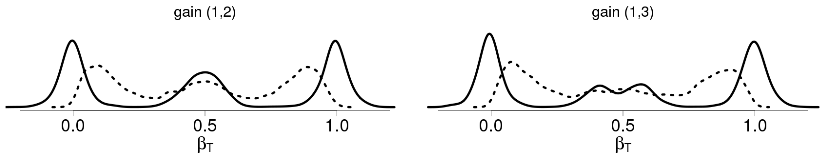

(a) gain (1,2). (b) gain (1,3).

Figure 9: Density of raw (dashed lines) and TumorBoost-normalized (solid lines) allele B fractions for region TCGA-02-0001:Chr2@75-110,cp=96+/-1,s=1/4.

4.3 ROC curves

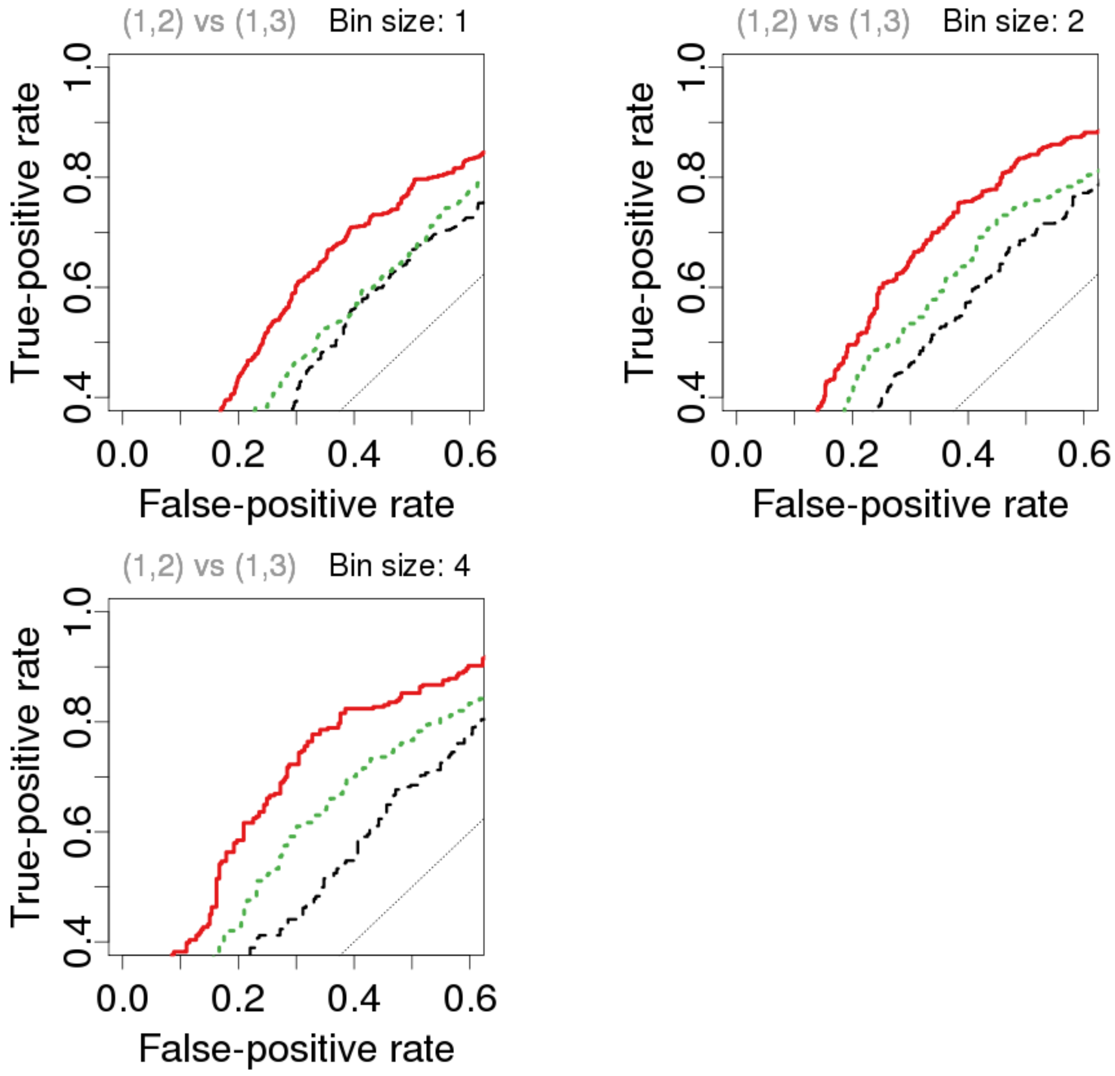

Figure 10: ROC curves for each preprocessing method at the full resolution as well as 2 different amounts of smoothing (using the mean() function) for region TCGA-02-0001:Chr2@75-110,cp=96+/-1,s=1/4. Legend: raw,NGC (dashed; #000000), TBN,NGC,NGC (solid; #E41A1C) and TCN,NGC (dotted; #4DAF4A).

4.4 (β_N, β_T) plots

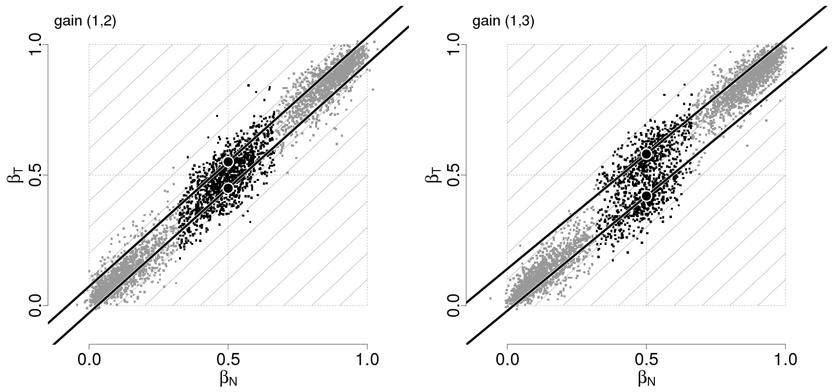

Figure 11: raw,NGC for region TCGA-02-0001:Chr2@75-110,cp=96+/-1,s=1/4.

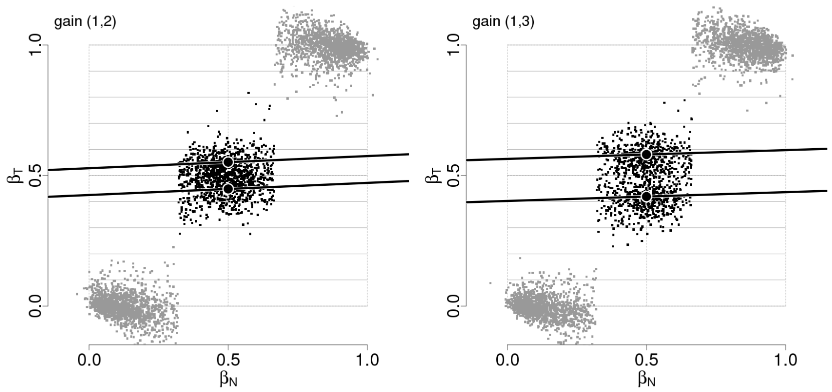

Figure 12: TBN,NGC,NGC for region TCGA-02-0001:Chr2@75-110,cp=96+/-1,s=1/4.

4.5 Allele-specific copy number estimates

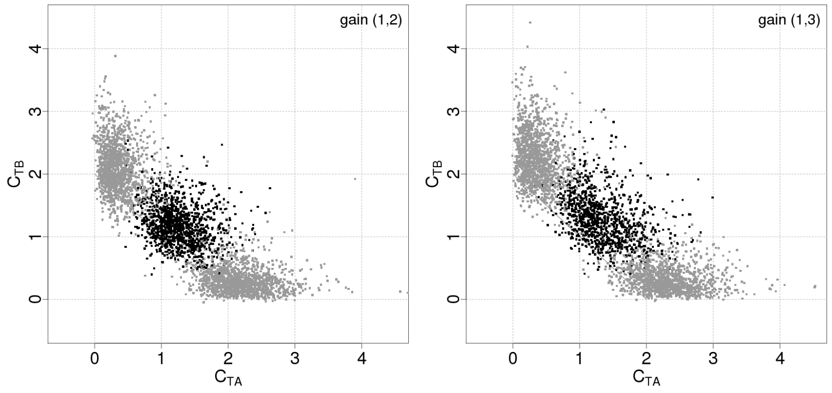

Figure 13: raw,NGC for region TCGA-02-0001:Chr2@75-110,cp=96+/-1,s=1/4.

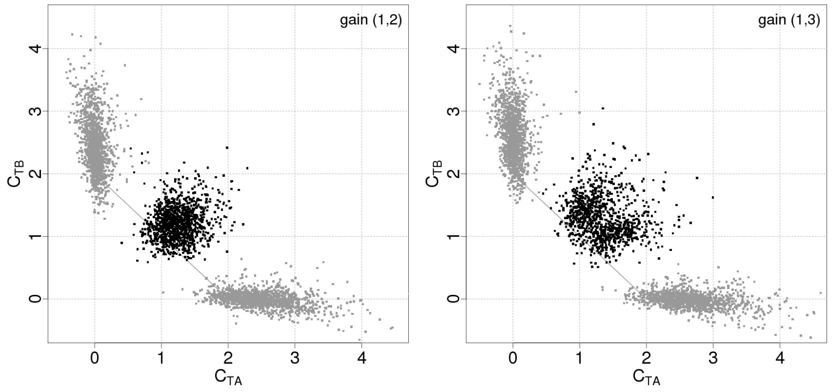

Figure 14: TBN,NGC,NGC for region TCGA-02-0001:Chr2@75-110,cp=96+/-1,s=1/4.

5 Region: TCGA-02-0001:Chr2@100-130,cp=110+/-1,s=4/0

5.1 Decrease in Heterozygosity (DH) and total copy-number tracks

(a) raw,NGC.

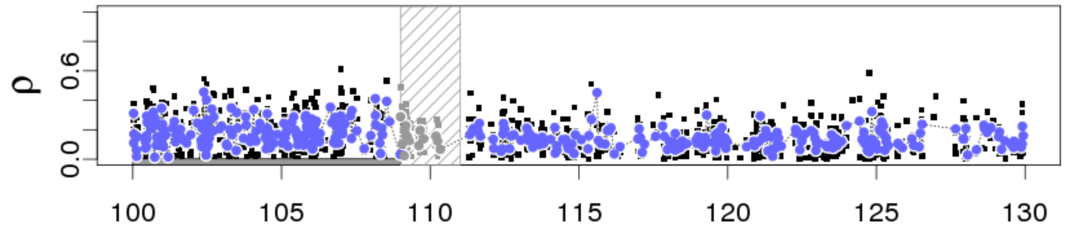

(b) TBN,NGC,NGC.

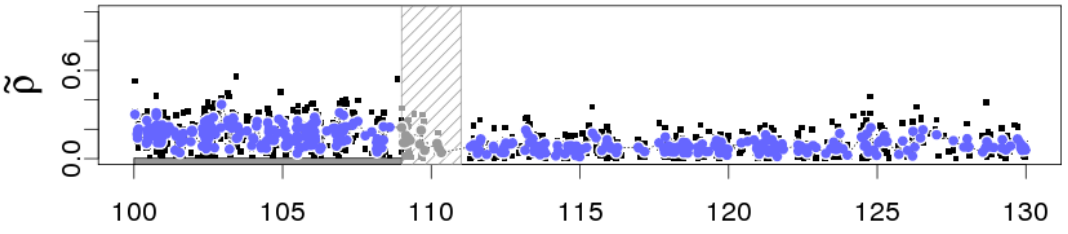

(c) TCN,NGC.

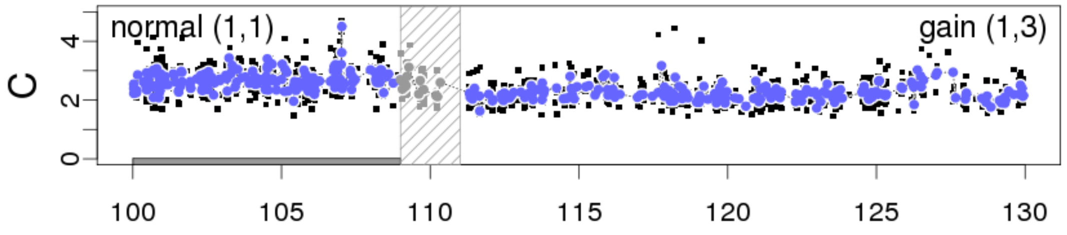

Figure 15: Decrease in Heterozygosity (DH) and total copy numbers for region TCGA-02-0001:Chr2@100-130,cp=110+/-1,s=4/0. Only heterozygous SNPs are plotted. There are 748 loci of state normal (1,1) ("negatives") and 748 loci of state gain (1,3) ("positives"), where the latter are highlighted with a solid bar beneath. In total 53 loci within the safety margin were excluded.

5.2 Allele B fraction density plots

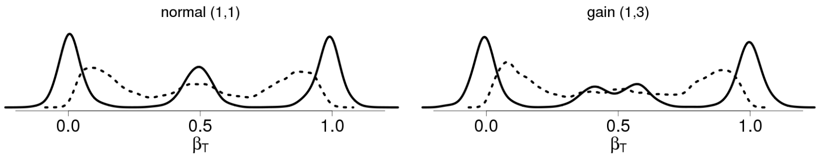

(a) normal (1,1). (b) gain (1,3).

Figure 16: Density of raw (dashed lines) and TumorBoost-normalized (solid lines) allele B fractions for region TCGA-02-0001:Chr2@100-130,cp=110+/-1,s=4/0.

5.3 ROC curves

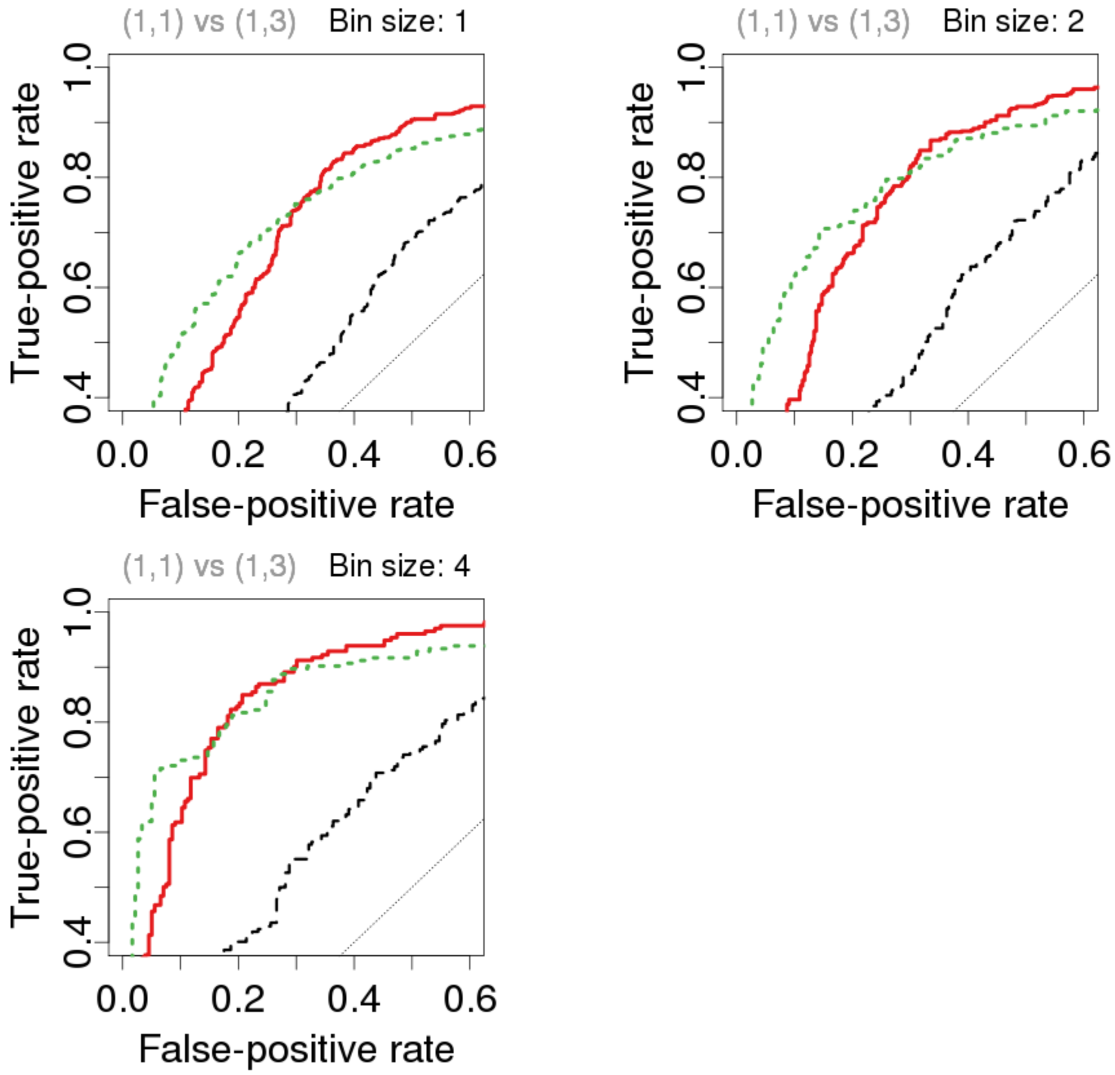

Figure 17: ROC curves for each preprocessing method at the full resolution as well as 2 different amounts of smoothing (using the mean() function) for region TCGA-02-0001:Chr2@100-130,cp=110+/-1,s=4/0. Legend: raw,NGC (dashed; #000000), TBN,NGC,NGC (solid; #E41A1C) and TCN,NGC (dotted; #4DAF4A).

5.4 (β_N, β_T) plots

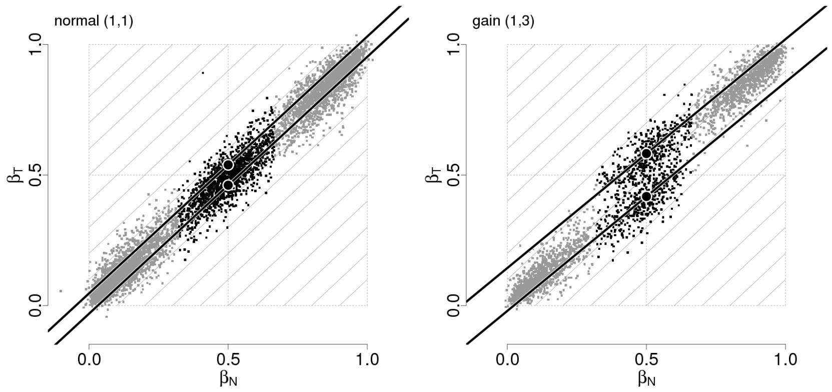

Figure 18: raw,NGC for region TCGA-02-0001:Chr2@100-130,cp=110+/-1,s=4/0.

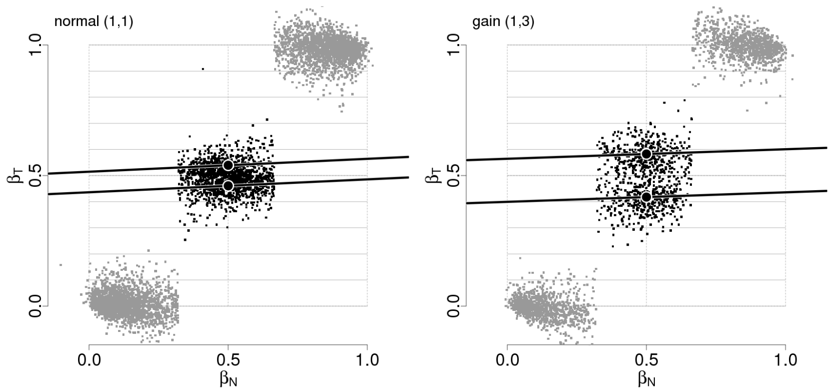

Figure 19: TBN,NGC,NGC for region TCGA-02-0001:Chr2@100-130,cp=110+/-1,s=4/0.

5.5 Allele-specific copy number estimates

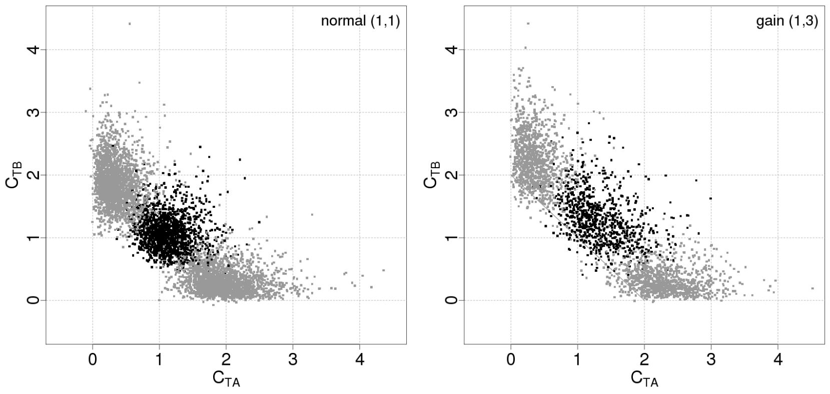

Figure 20: raw,NGC for region TCGA-02-0001:Chr2@100-130,cp=110+/-1,s=4/0.

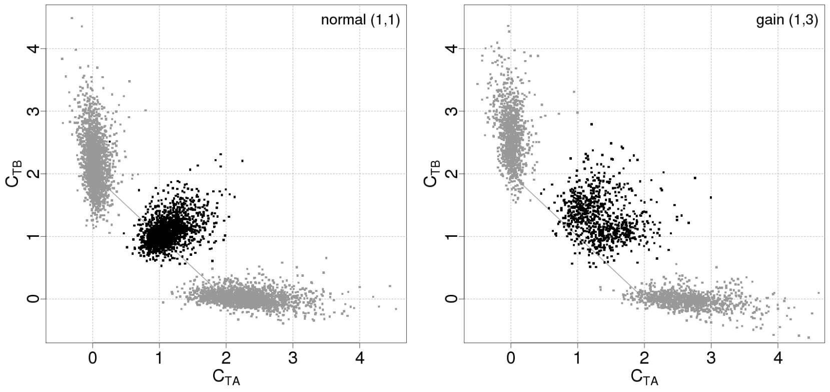

Figure 21: TBN,NGC,NGC for region TCGA-02-0001:Chr2@100-130,cp=110+/-1,s=4/0.

6 Region: TCGA-02-0001:Chr13@0-70,cp=45+/-1,s=0/2

6.1 Decrease in Heterozygosity (DH) and total copy-number tracks

(a) raw,NGC.

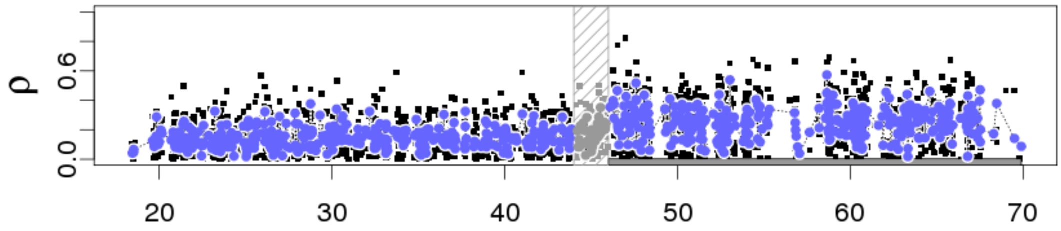

(b) TBN,NGC,NGC.

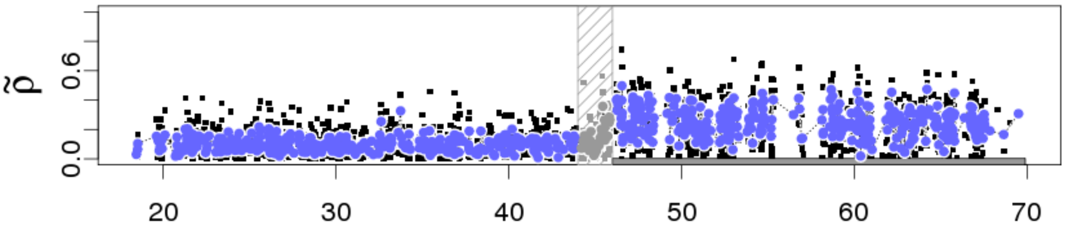

(c) TCN,NGC.

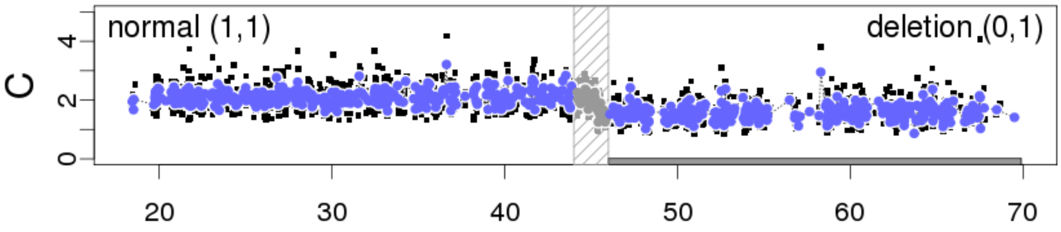

Figure 22: Decrease in Heterozygosity (DH) and total copy numbers for region TCGA-02-0001:Chr13@0-70,cp=45+/-1,s=0/2. Only heterozygous SNPs are plotted. There are 1418 loci of state normal (1,1) (”negatives”) and 1418 loci of state deletion (0,1) (”positives”), where the latter are highlighted with a solid bar beneath. In total 126 loci within the safety margin were excluded.

6.2 Allele B fraction density plots

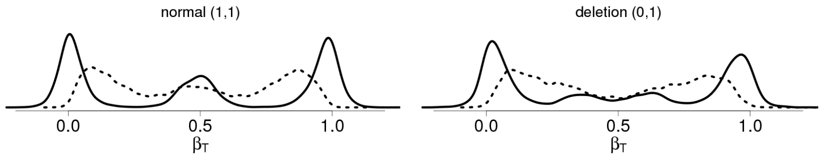

(a) normal (1,1). (b) deletion (0,1).

Figure 23: Density of raw (dashed lines) and TumorBoost-normalized (solid lines) allele B fractions for region TCGA-02-0001:Chr13@0-70,cp=45+/-1,s=0/2.

6.3 ROC curves

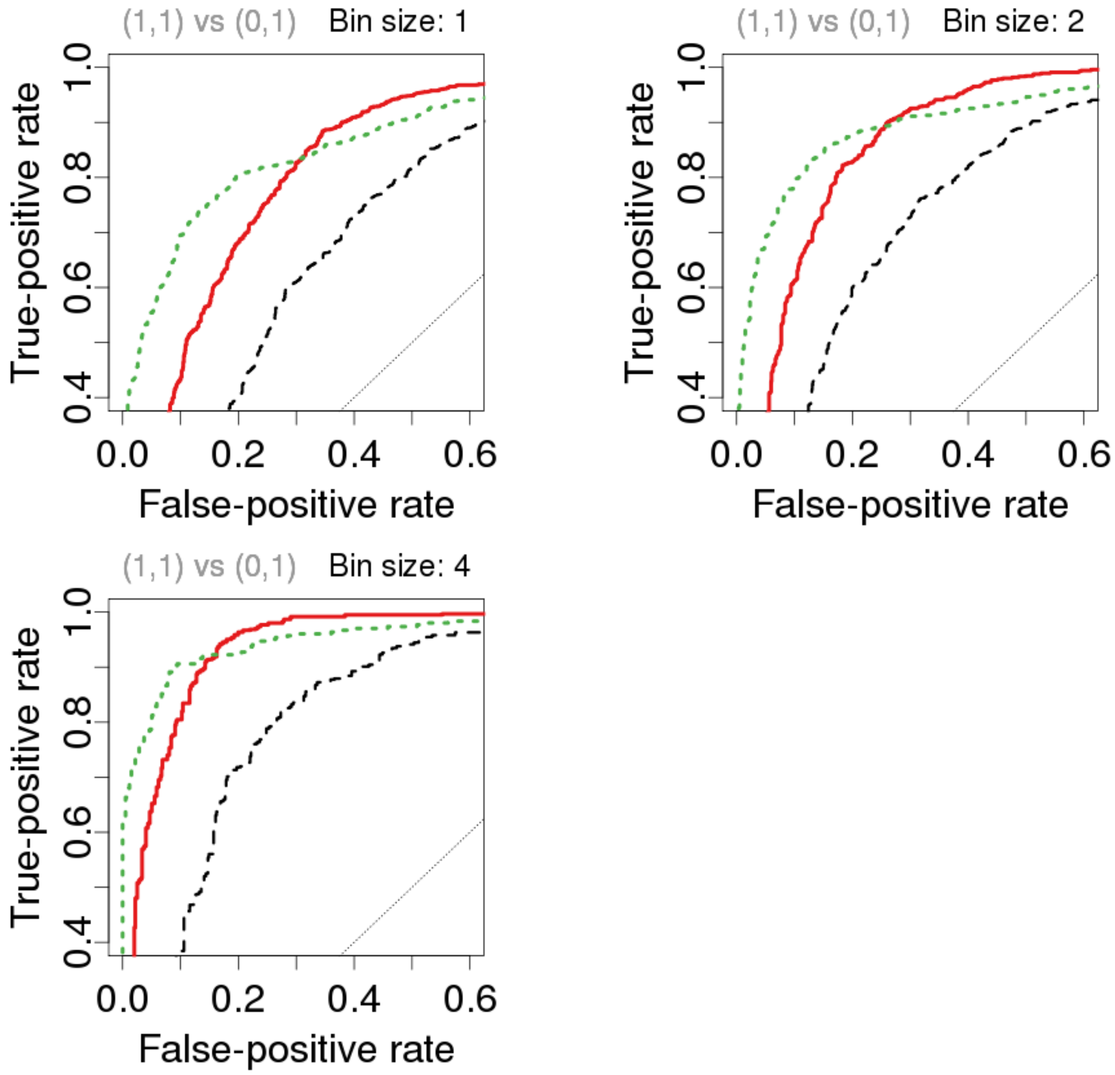

Figure 24: ROC curves for each preprocessing method at the full resolution as well as 2 different amounts of smoothing (using the mean() function) for region TCGA-02-0001:Chr13@0-70,cp=45+/-1,s=0/2. Legend: raw,NGC (dashed; #000000), TBN,NGC,NGC (solid; #E41A1C) and TCN,NGC (dotted; #4DAF4A).

6.4 (β_N, β_T) plots

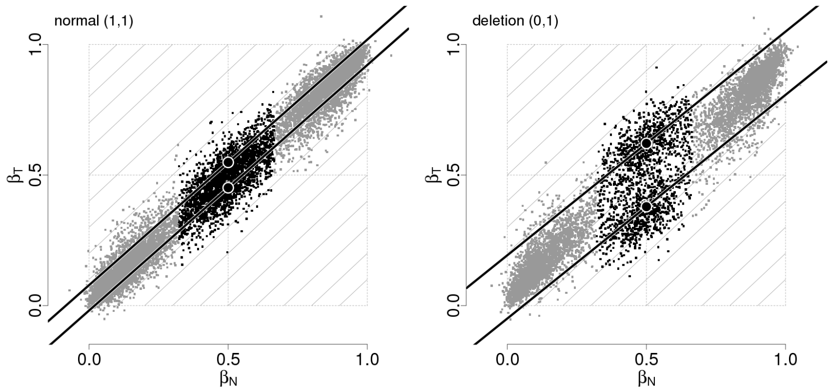

Figure 25: raw,NGC for region TCGA-02-0001:Chr13@0-70,cp=45+/-1,s=0/2.

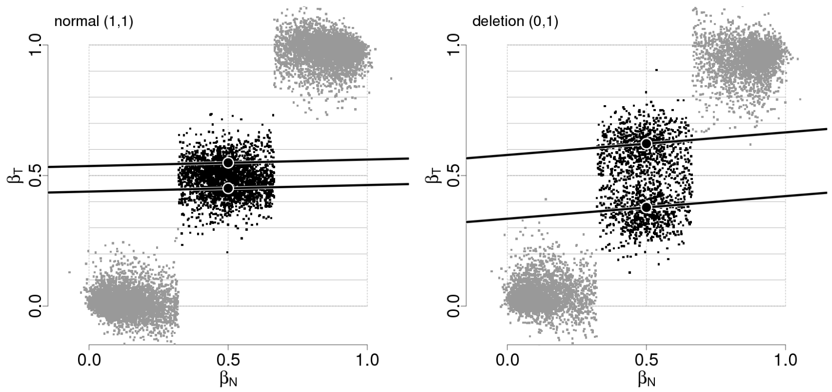

Figure 26: TBN,NGC,NGC for region TCGA-02-0001:Chr13@0-70,cp=45+/-1,s=0/2.

6.5 Allele-specific copy number estimates

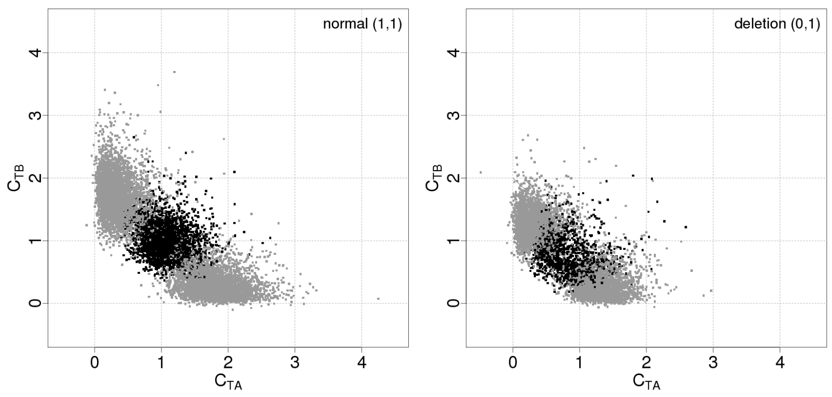

Figure 27: raw,NGC for region TCGA-02-0001:Chr13@0-70,cp=45+/-1,s=0/2.

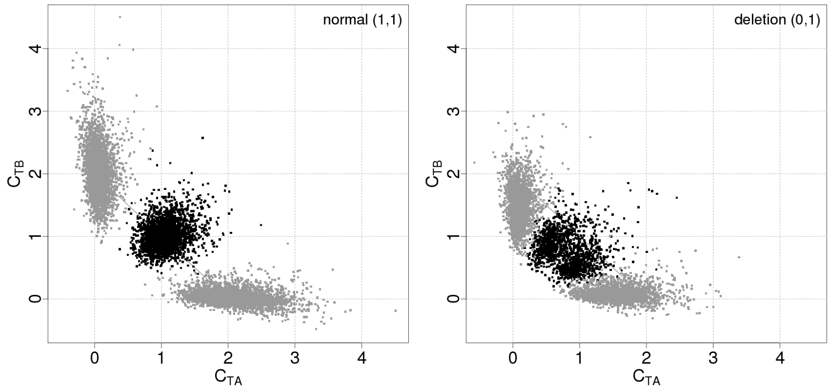

Figure 28: TBN,NGC,NGC for region TCGA-02-0001:Chr13@0-70,cp=45+/-1,s=0/2.

7 Bootstrap estimates of test statistics for all regions

	1/2	1/4	4/0	0/2
raw,NGC	12.548±1.223	3.757±1.102	5.203±0.974	9.565±1.001
TBN,NGC,NGC	17.739±1.345	7.152±0.960	10.640±1.205	14.541±1.076
TCN,NGC	19.977±1.294	4.916±0.945	11.174±1.080	14.894±1.254

Table 2: Student test statistics of the null hypothesis of equal mean before and after each PCN change point (heterozygous SNPs): raw or TumorBoost-normalized DH, and total copy number (last line). Mean ± standard deviation across 100 samplings of 225 points (for each PCN state) from the original data set. The larger value, the more different the true means are.

References

Bengtsson, H., Wirapati, P., and Speed, T. P. (2009). A single-array preprocessing method for estimating full-resolution raw copy numbers from all Affymetrix genotyping arrays including GenomeWideSNP 5 & 6. *Bioinformatics*, **25**(17), 2149–2156.

A Data files

A.1 Total copy numbers

$‘TCGA,GBM,CRMAv2‘
AromaUnitTotalCnBinarySet:
Name: TCGA
Tags: GBM,CRMAv2
Full name: TCGA,GBM,CRMAv2
Number of files: 1
Names: TCGA-02-0001 [1]
Path (to the first file): rawCnData/TCGA,GBM,CRMAv2/GenomeWideSNP˙6
Total file size: 7.08 MB
RAM: 0.00MB

A.2 Allele B fractions

$raw
AromaUnitFracBCnBinarySet:
Name: TCGA
Tags: GBM,CRMAv2
Full name: TCGA,GBM,CRMAv2
Number of files: 1
Names: TCGA-02-0001 [1]
Path (to the first file): totalAndFracBData/TCGA,GBM,CRMAv2/GenomeWideSNP˙6
Total file size: 7.08 MB
RAM: 0.00MB

$‘TBN,NGC‘
AromaUnitFracBCnBinarySet:
Name: TCGA
Tags: GBM,CRMAv2,TBN,NGC
Full name: TCGA,GBM,CRMAv2,TBN,NGC
Number of files: 1
Names: TCGA-02-0001 [1]
Path (to the first file): totalAndFracBData/TCGA,GBM,CRMAv2,TBN,NGC/GenomeWideSNP˙6
Total file size: 7.08 MB
RAM: 0.00MB

A.3 Genotype calls

$NGC
AromaUnitGenotypeCallSet:
Name: TCGA
Tags: GBM,CRMAv2,NGC
Full name: TCGA,GBM,CRMAv2,NGC
Number of files: 1
Names: TCGA-02-0001 [1]
Path (to the first file): callData/TCGA,GBM,CRMAv2,NGC/GenomeWideSNP˙6
Total file size: 3.54 MB
RAM: 0.00MB

A.4 Genotype confidence scores

$NGC
AromaUnitSignalBinarySet:

Name: TCGA
Tags: GBM,CRMAv2,NGC
Full name: TCGA,GBM,CRMAv2,NGC
Number of files: 1
Names: TCGA-02-0001 [1]
Path (to the first file): callData/TCGA,GBM,CRMAv2,NGC/GenomeWideSNP˙6
Total file size: 7.08 MB
RAM: 0.00MB

B Session information

This report was automatically generated using the R.rsp package.

- R version 2.10.1 (2009-12-14), `x86_64-unknown-linux-gnu`
- Locale: `LC_CTYPE=en_US.UTF-8`, `LC_NUMERIC=C`, `LC_TIME=en_US.UTF-8`, `LC_COLLATE=en_US.UTF-8`, `LC_MONETARY=C`, `LC_MESSAGES=en_US.UTF-8`, `LC_PAPER=en_US.UTF-8`, `LC_NAME=C`, `LC_ADDRESS=C`, `LC_TELEPHONE=C`, `LC_MEASUREMENT=en_US.UTF-8`, `LC_IDENTIFICATION=C`
- Base packages: base, datasets, graphics, grDevices, methods, stats, utils
- Other packages: aroma.cn.eval 0.1.1, aroma.core 1.5.0, aroma.light 1.15.1, digest 0.4.2, MASS 7.3-4, matrixStats 0.1.9, R.cache 0.2.0, RColorBrewer 1.0-2, R.filesets 0.8.0, R.menu 0.1.0, R.methodsS3 1.1.0, R.oo 1.6.7, R.rsp 0.3.6, R.utils 1.3.3, xtable 1.5-6
- Loaded via a namespace (and not attached): affxparser 1.18.0, tools 2.10.1
