# Supplementary material for: TumorBoost: Normalization of allele-specific tumor copy numbers from a single pair of tumor-normal genotyping microarrays
Source: BMC Bioinformatics. 2010 May 12;11:245. doi: 10.1186/1471-2105-11-245 (PMC2894037; doi:10.1186/1471-2105-11-245)
Supplement: Additional file 12 — Illumina HumanHap550 data after BeadStudio,XY preprocessing (sample TCGA-02-0001; with confidence scores). Assessment of TumorBoost based on tumor/normal pair TCGA-02-0001 in the Illumina HumanHap550 data set preprocessed with the BeadStudio,XY method using the SNPs with 90% highest confidence scores. [file 1471-2105-11-245-S12.PDF]

Supplementary Note: Assessment of TumorBoost based on tumor/normal pair TCGA-02-0001 in the Illumina HumanHap550 data set TCGA,GBM,BeadStudio,XY using the SNPs with 90% highest confidence scores

Henrik Bengtsson, Pierre Neuvial, Terence P. Speed

March 5, 2010

Contents

1 Introduction **3**

2 Data set **3**
2.1 Preprocessing methods . . . 3
2.2 Stratification on genotype confidence scores . . . 3
2.3 List of change points . . . 3

3 Region: TCGA-02-0001:Chr2@35-74,cp=57+/-1,s=1/2 **4**
3.1 Decrease in Heterozygosity (DH) and total copy-number tracks . . . 4
3.2 Allele B fraction density plots . . . 5
3.3 ROC curves . . . 5
3.4 (β_N, β_T) plots . . . 6
3.5 Allele-specific copy number estimates . . . 7

4 Region: TCGA-02-0001:Chr2@75-110,cp=96+/-1,s=1/4 **8**
4.1 Decrease in Heterozygosity (DH) and total copy-number tracks . . . 8
4.2 Allele B fraction density plots . . . 9
4.3 ROC curves . . . 9
4.4 (β_N, β_T) plots . . . 10
4.5 Allele-specific copy number estimates . . . 11

5 Region: TCGA-02-0001:Chr2@100-130,cp=110+/-1,s=4/0 **12**
5.1 Decrease in Heterozygosity (DH) and total copy-number tracks . . . 12
5.2 Allele B fraction density plots . . . 13
5.3 ROC curves . . . 13
5.4 (β_N, β_T) plots . . . 14
5.5 Allele-specific copy number estimates . . . 15

6 Region: TCGA-02-0001:Chr13@0-70,cp=45+/-1,s=0/2 **16**
6.1 Decrease in Heterozygosity (DH) and total copy-number tracks . . . 16
6.2 Allele B fraction density plots . . . 17
6.3 ROC curves . . . 17
6.4 (β_N, β_T) plots . . . 18
6.5 Allele-specific copy number estimates . . . 19

7 Bootstrap estimates of test statistics for all regions **20**

A Data files **21**
A.1 Total copy numbers 21
A.2 Allele B fractions 21
A.3 Genotype calls 21
A.4 Genotype confidence scores 21

B Session information **23**

1 Introduction

This report, which is automatically generated, assesses the performance of the TumorBoost method based on a few change points in a particular tumor/normal pair. For more details on the evaluation methods, see the main TumorBoost manuscript.

2 Data set

The evaluation is this report is based on the tumor/normal pair (01C,10A) for individual TCGA-02-0001 in the data set TCGA,GBM,BeadStudio,XY.

2.1 Preprocessing methods

The data was generated on the Illumina HumanHap550 chip type. Each array was preprocessed separately using Birdseed's "XY-normalization" method, which is principle is a single-array method that relies neither on reference samples nor prior estimates.

2.2 Stratification on genotype confidence scores

We focus on the SNPs in which we are the most confident that they are heterozygous: the evaluation will involve the 90% SNPs with highest genotype confidence scores.

2.3 List of change points

For this data set, we have selected a few regions for which one can safely assume that there exists a single copy number change point. By definition, each change point separates two sets of genomic loci such that the true Decrease in Heterozygosity (DH) is the same within one set of loci but differs between the two sets. These regions were selected visually. For each region we chose a large enough safety margin to make our evaluation independent of the uncertainty on the true location of the change point.

Chr	Region	Change point	Margin	Before	After
2	35-74	57	1	gain (1,2)	deletion (0,1)
2	75-110	96	1	gain (1,2)	gain (1,3)
2	100-130	110	1	gain (1,3)	normal (1,1)
13	0-70	45	1	normal (1,1)	deletion (0,1)

Table 1: Regions in TCGA-02-0001 used for the evaluation and that each contain a single changepoint. All positions and lengths are in units of Mb.

We next compare how well each of these change points is detected using the above preprocessed signals followed or not by TumorBoost normalization using the ROC analysis described in the main TumorBoost manuscript at the full resolution as well as smoothed resolution with bin sizes $h = \{1, 2, 4\}$. Specifically, we compare the following three methods: (1) **"raw"**: preprocessed signals without TumorBoost normalization. (2) **"TBN,NGC"**: preprocessed signals followed by TumorBoost normalization using NGC genotype calls. For completeness we also include an evaluation of Total copy numbers (TCN).

3 Region: TCGA-02-0001:Chr2@35-74,cp=57+/-1,s=1/2

3.1 Decrease in Heterozygosity (DH) and total copy-number tracks

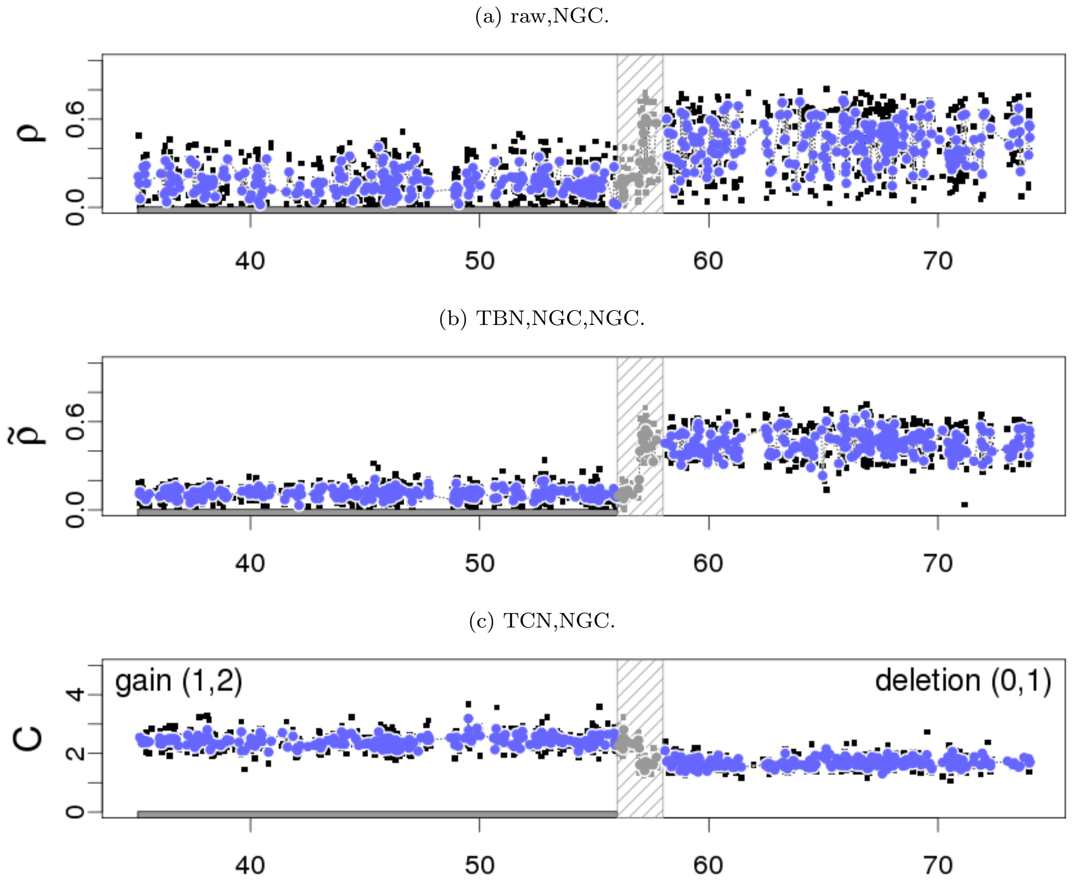

Figure 1: Decrease in Heterozygosity (DH) and total copy numbers for region TCGA-02-0001:Chr2@35-74,cp=57+/-1,s=1/2. Only heterozygous SNPs are plotted. There are 829 loci of state gain (1,2) (”negatives”) and 829 loci of state deletion (0,1) (”positives”), where the latter are highlighted with a solid bar beneath. In total 91 loci within the safety margin were excluded.

3.2 Allele B fraction density plots

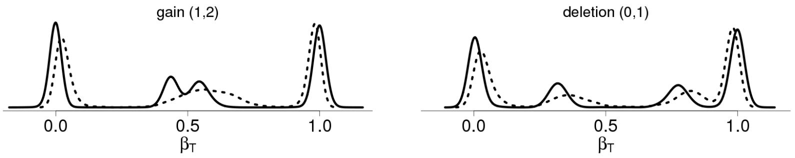

(a) gain (1,2). (b) deletion (0,1).

Figure 2: Density of raw (dashed lines) and TumorBoost-normalized (solid lines) allele B fractions for region TCGA-02-0001:Chr2@35-74,cp=57+/-1,s=1/2.

3.3 ROC curves

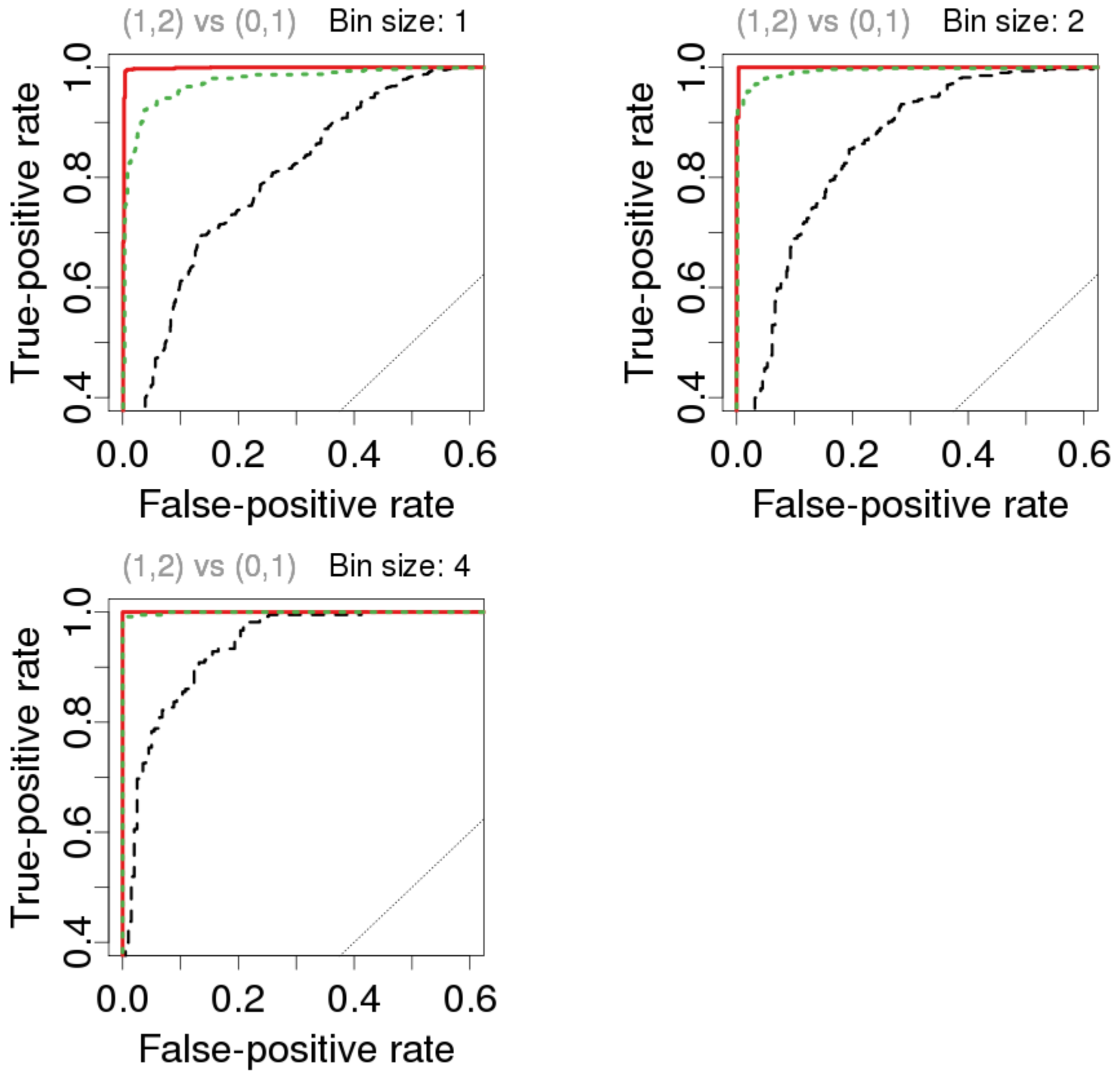

Figure 3: ROC curves for each preprocessing method at the full resolution as well as 2 different amounts of smoothing (using the mean() function) for region TCGA-02-0001:Chr2@35-74,cp=57+/-1,s=1/2. Legend: raw,NGC (dashed; #000000), TBN,NGC,NGC (solid; #E41A1C) and TCN,NGC (dotted; #4DAF4A).

3.4 (β_N, β_T) plots

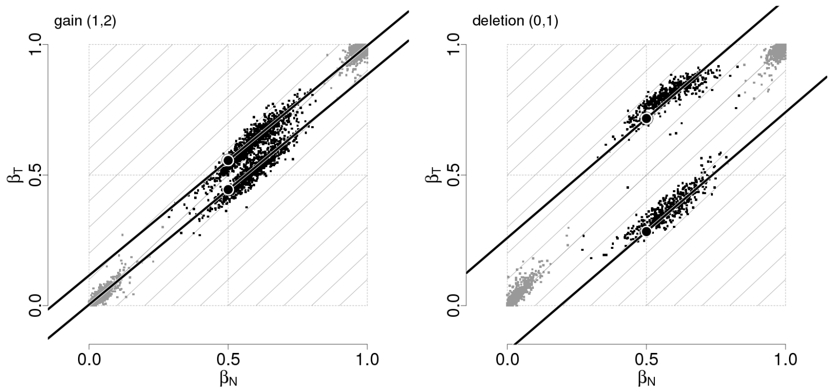

Figure 4: raw,NGC for region TCGA-02-0001:Chr2@35-74,cp=57+/-1,s=1/2.

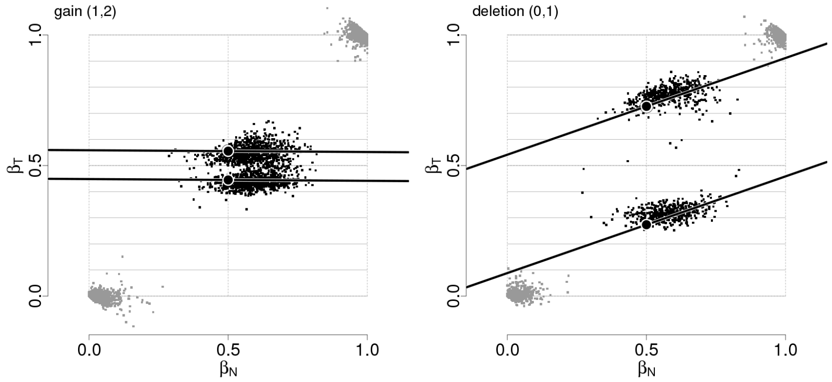

Figure 5: TBN,NGC,NGC for region TCGA-02-0001:Chr2@35-74,cp=57+/-1,s=1/2.

3.5 Allele-specific copy number estimates

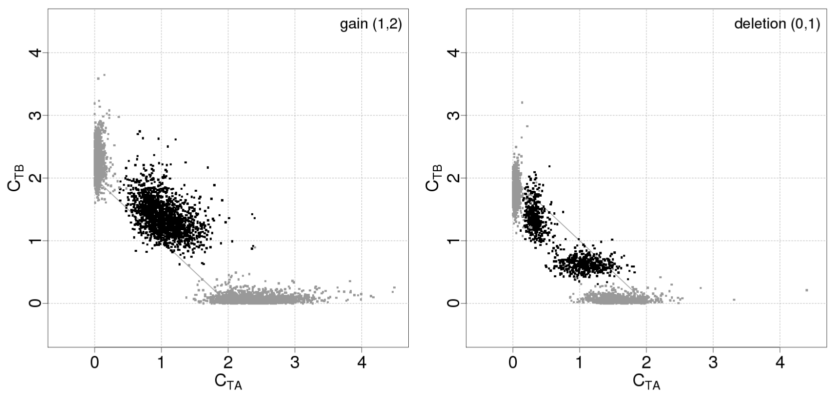

Figure 6: raw,NGC for region TCGA-02-0001:Chr2@35-74,cp=57+/-1,s=1/2.

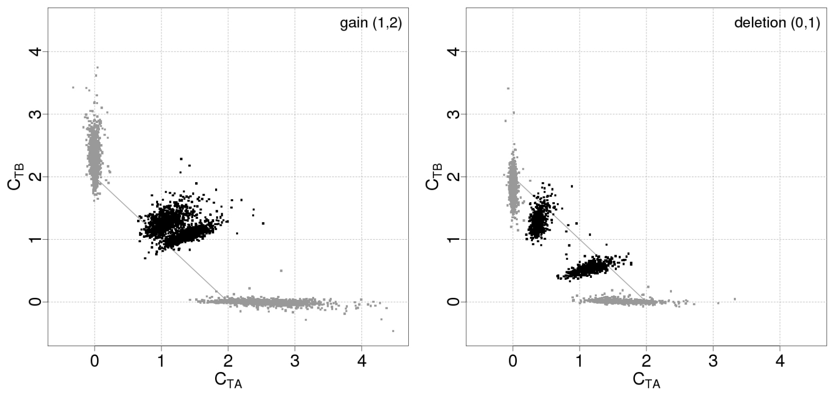

Figure 7: TBN,NGC,NGC for region TCGA-02-0001:Chr2@35-74,cp=57+/-1,s=1/2.

4 Region: TCGA-02-0001:Chr2@75-110,cp=96+/-1,s=1/4

4.1 Decrease in Heterozygosity (DH) and total copy-number tracks

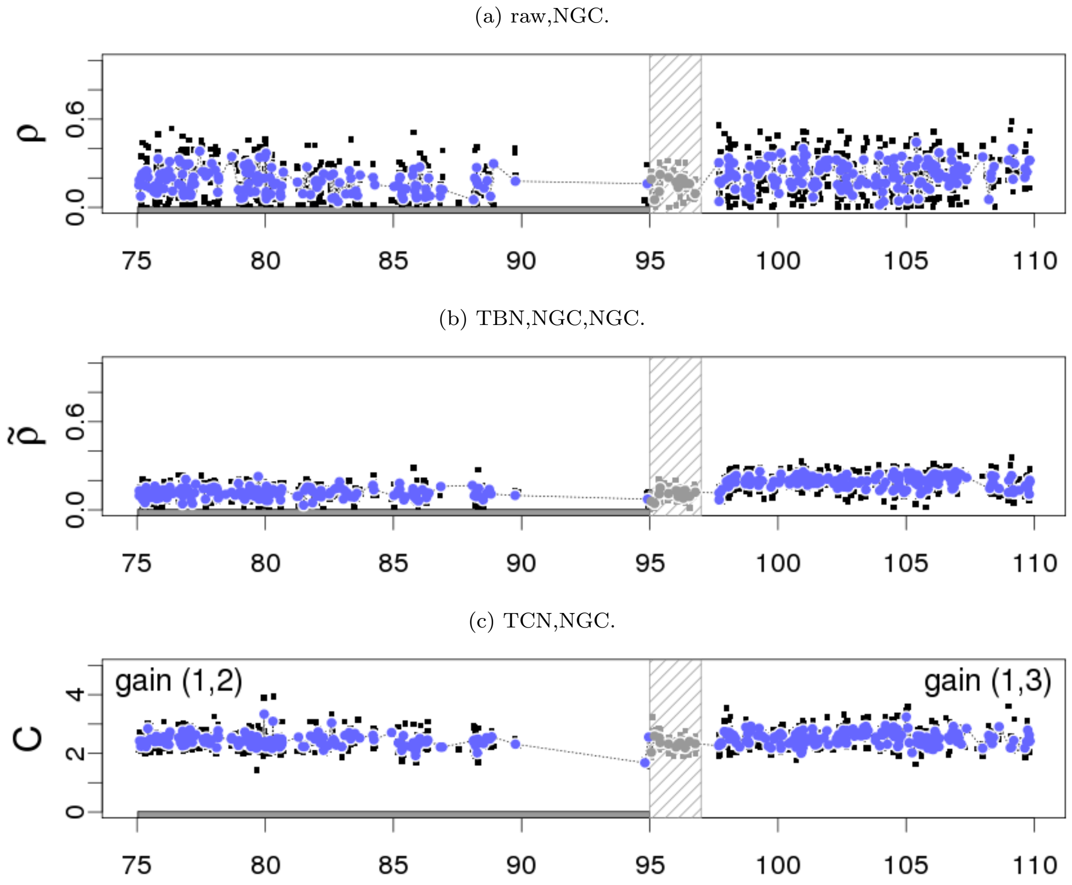

Figure 8: Decrease in Heterozygosity (DH) and total copy numbers for region TCGA-02-0001:Chr2@75-110,cp=96+/-1,s=1/4. Only heterozygous SNPs are plotted. There are 626 loci of state gain (1,2) ("negatives") and 626 loci of state gain (1,3) ("positives"), where the latter are highlighted with a solid bar beneath. In total 49 loci within the safety margin were excluded.

4.2 Allele B fraction density plots

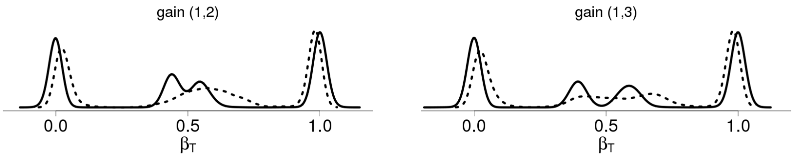

(a) gain (1,2). (b) gain (1,3).

Figure 9: Density of raw (dashed lines) and TumorBoost-normalized (solid lines) allele B fractions for region TCGA-02-0001:Chr2@75-110,cp=96+/-1,s=1/4.

4.3 ROC curves

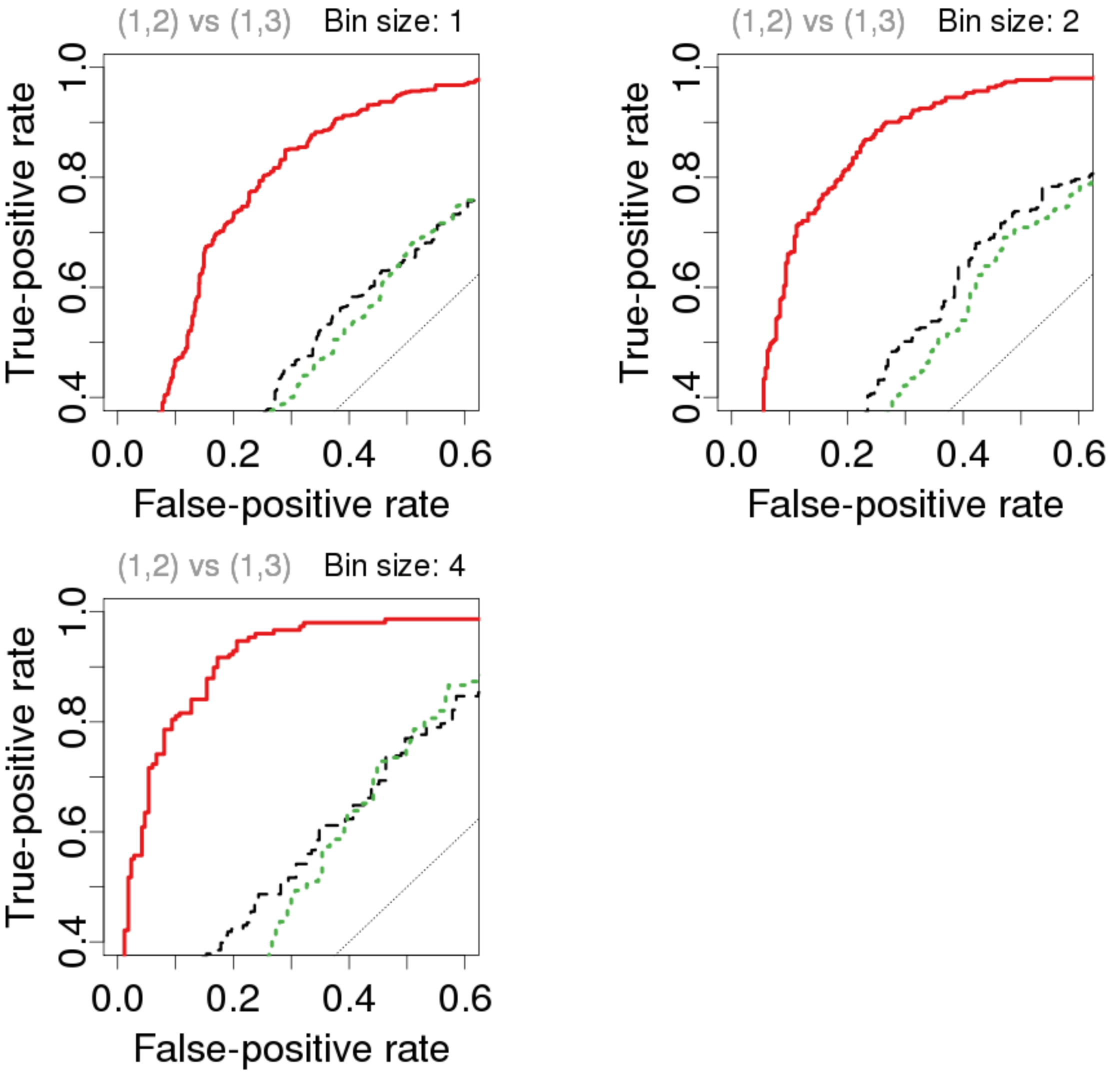

Figure 10: ROC curves for each preprocessing method at the full resolution as well as 2 different amounts of smoothing (using the mean() function) for region TCGA-02-0001:Chr2@75-110,cp=96+/-1,s=1/4. Legend: raw,NGC (dashed; #000000), TBN,NGC,NGC (solid; #E41A1C) and TCN,NGC (dotted; #4DAF4A).

4.4 (β_N, β_T) plots

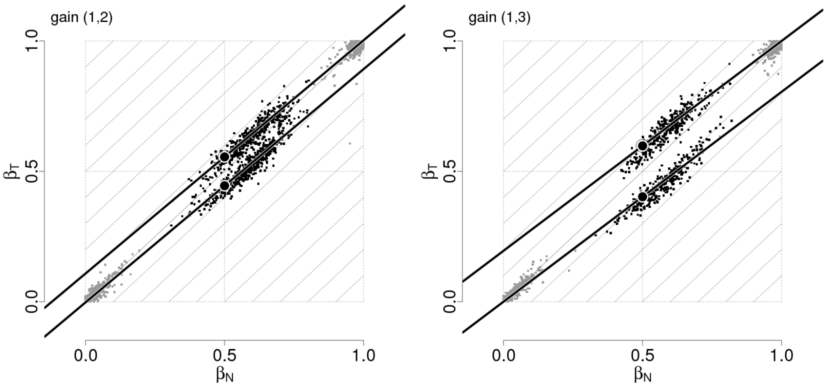

Figure 11: raw,NGC for region TCGA-02-0001:Chr2@75-110,cp=96+/-1,s=1/4.

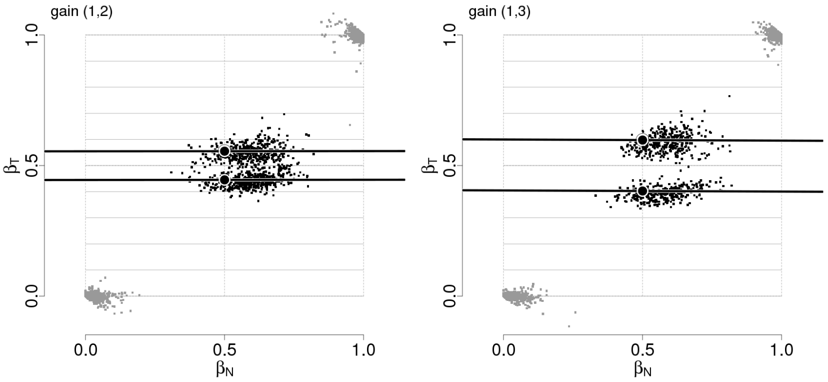

Figure 12: TBN,NGC,NGC for region TCGA-02-0001:Chr2@75-110,cp=96+/-1,s=1/4.

4.5 Allele-specific copy number estimates

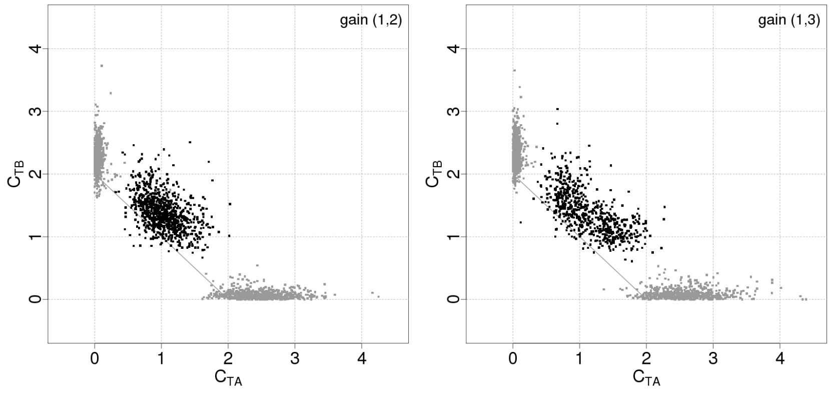

Figure 13: raw,NGC for region TCGA-02-0001:Chr2@75-110,cp=96+/-1,s=1/4.

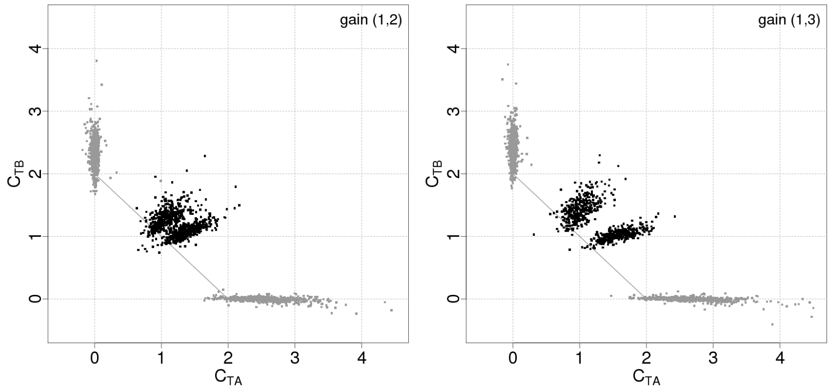

Figure 14: TBN,NGC,NGC for region TCGA-02-0001:Chr2@75-110,cp=96+/-1,s=1/4.

5 Region: TCGA-02-0001:Chr2@100-130,cp=110+/-1,s=4/0

5.1 Decrease in Heterozygosity (DH) and total copy-number tracks

(a) raw,NGC.

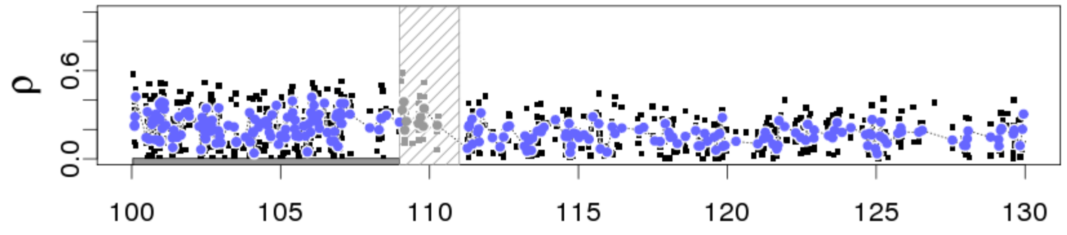

(b) TBN,NGC,NGC.

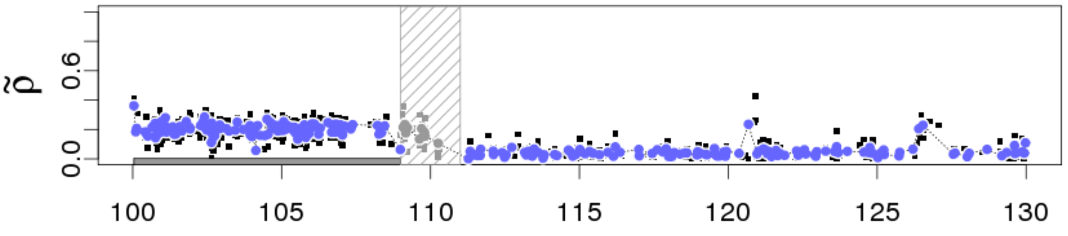

(c) TCN,NGC.

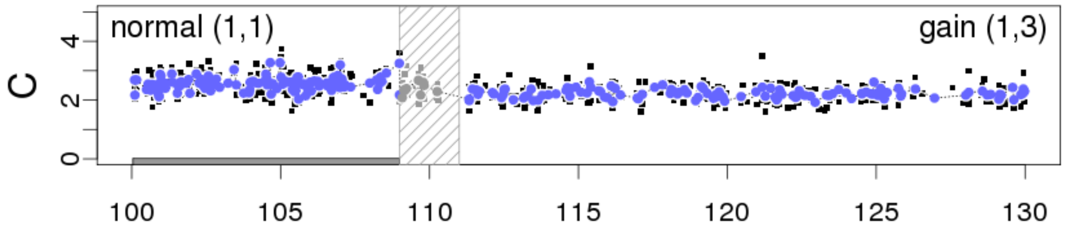

Figure 15: Decrease in Heterozygosity (DH) and total copy numbers for region TCGA-02-0001:Chr2@100-130,cp=110+/-1,s=4/0. Only heterozygous SNPs are plotted. There are 469 loci of state normal (1,1) ("negatives") and 469 loci of state gain (1,3) ("positives"), where the latter are highlighted with a solid bar beneath. In total 39 loci within the safety margin were excluded.

5.2 Allele B fraction density plots

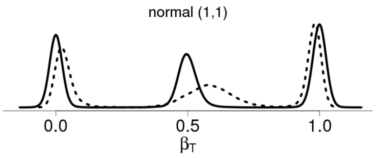

(a) normal (1,1).

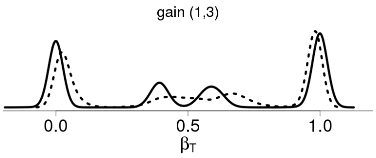

(b) gain (1,3).

Figure 16: Density of raw (dashed lines) and TumorBoost-normalized (solid lines) allele B fractions for region TCGA-02-0001:Chr2@100-130,cp=110+/-1,s=4/0.

5.3 ROC curves

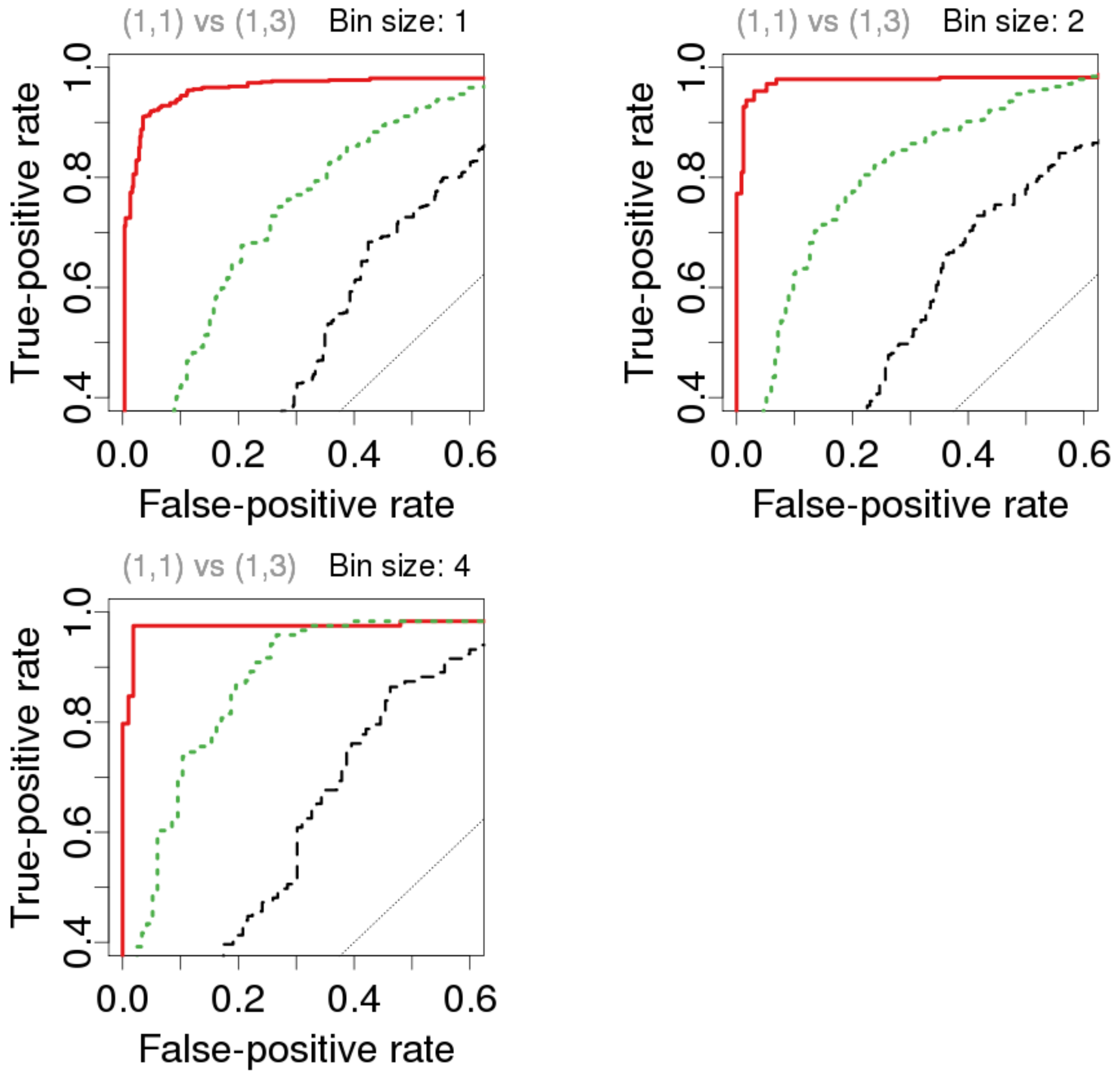

Figure 17: ROC curves for each preprocessing method at the full resolution as well as 2 different amounts of smoothing (using the mean() function) for region TCGA-02-0001:Chr2@100-130,cp=110+/-1,s=4/0. Legend: raw,NGC (dashed; #000000), TBN,NGC,NGC (solid; #E41A1C) and TCN,NGC (dotted; #4DAF4A).

5.4 (β_N, β_T) plots

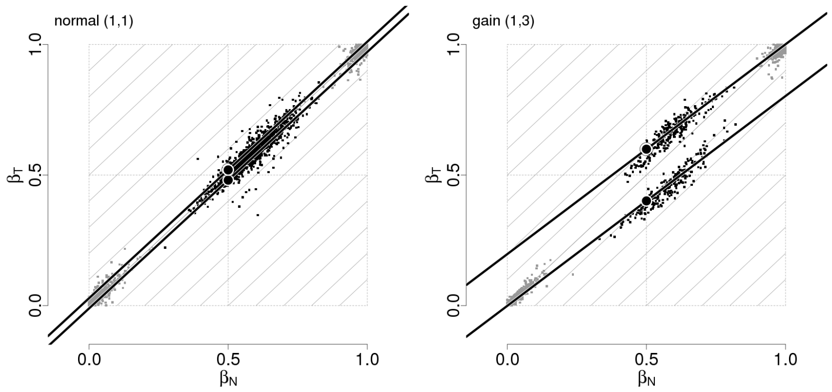

Figure 18: raw,NGC for region TCGA-02-0001:Chr2@100-130,cp=110+/-1,s=4/0.

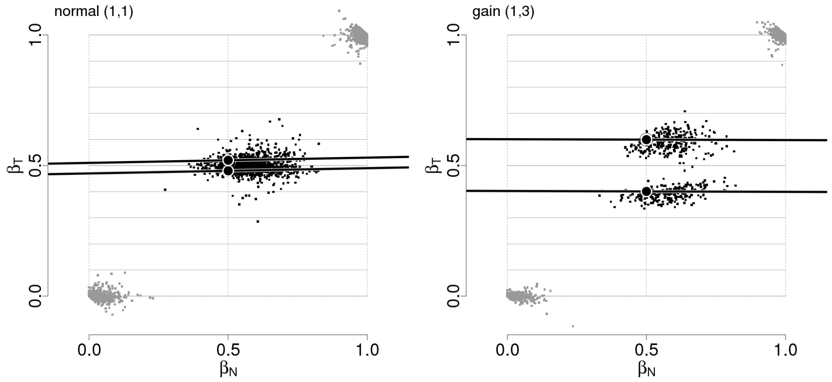

Figure 19: TBN,NGC,NGC for region TCGA-02-0001:Chr2@100-130,cp=110+/-1,s=4/0.

5.5 Allele-specific copy number estimates

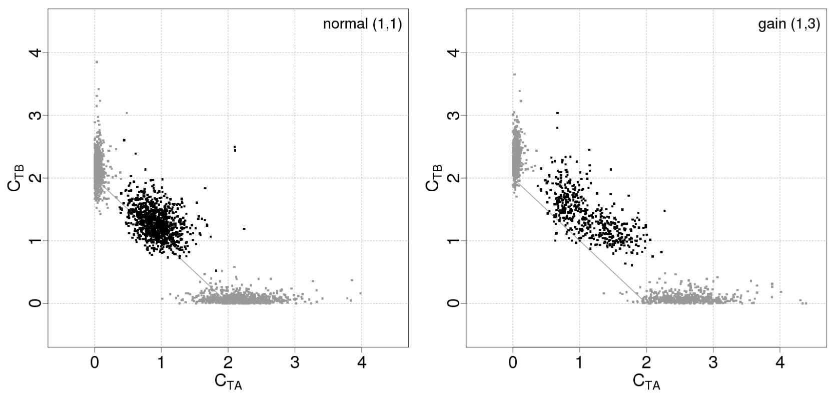

Figure 20: raw,NGC for region TCGA-02-0001:Chr2@100-130,cp=110+/-1,s=4/0.

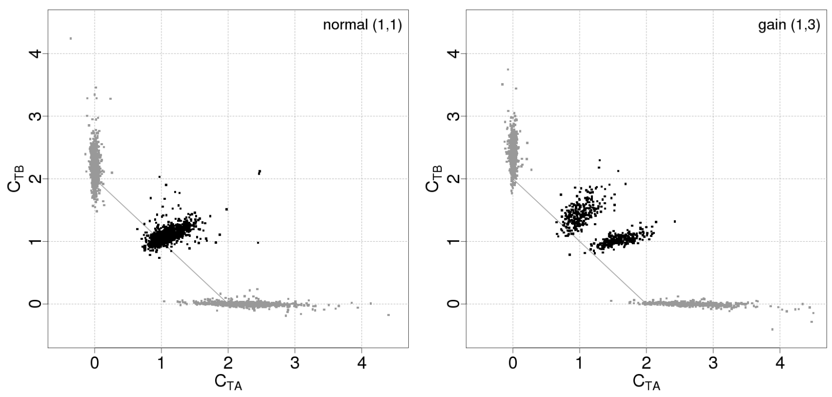

Figure 21: TBN,NGC,NGC for region TCGA-02-0001:Chr2@100-130,cp=110+/-1,s=4/0.

6 Region: TCGA-02-0001:Chr13@0-70,cp=45+/-1,s=0/2

6.1 Decrease in Heterozygosity (DH) and total copy-number tracks

(a) raw,NGC.

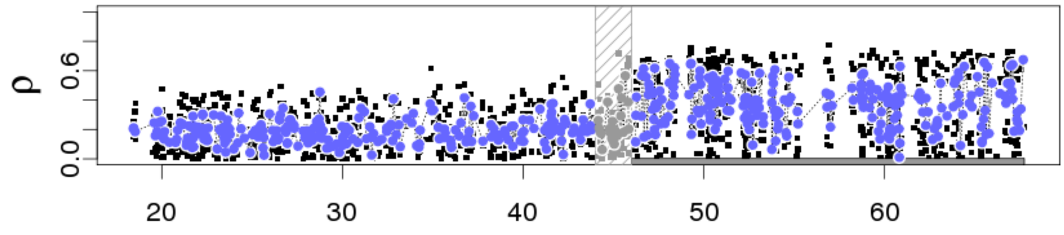

(b) TBN,NGC,NGC.

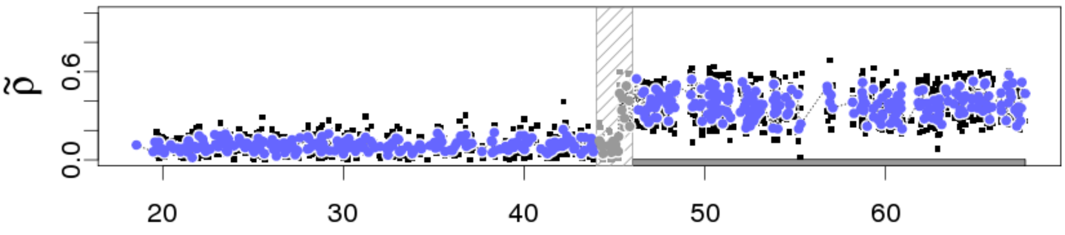

(c) TCN,NGC.

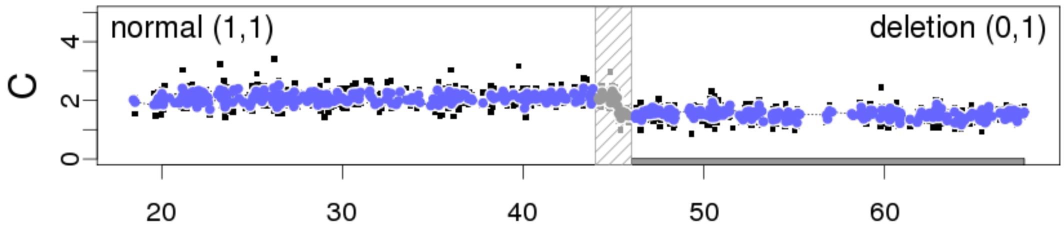

Figure 22: Decrease in Heterozygosity (DH) and total copy numbers for region TCGA-02-0001:Chr13@0-70,cp=45+/-1,s=0/2. Only heterozygous SNPs are plotted. There are 943 loci of state normal (1,1) ("negatives") and 943 loci of state deletion (0,1) ("positives"), where the latter are highlighted with a solid bar beneath. In total 87 loci within the safety margin were excluded.

6.2 Allele B fraction density plots

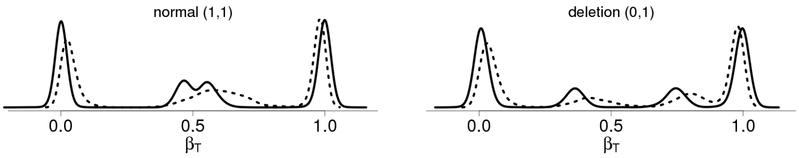

(a) normal (1,1). (b) deletion (0,1).

Figure 23: Density of raw (dashed lines) and TumorBoost-normalized (solid lines) allele B fractions for region TCGA-02-0001:Chr13@0-70,cp=45+/-1,s=0/2.

6.3 ROC curves

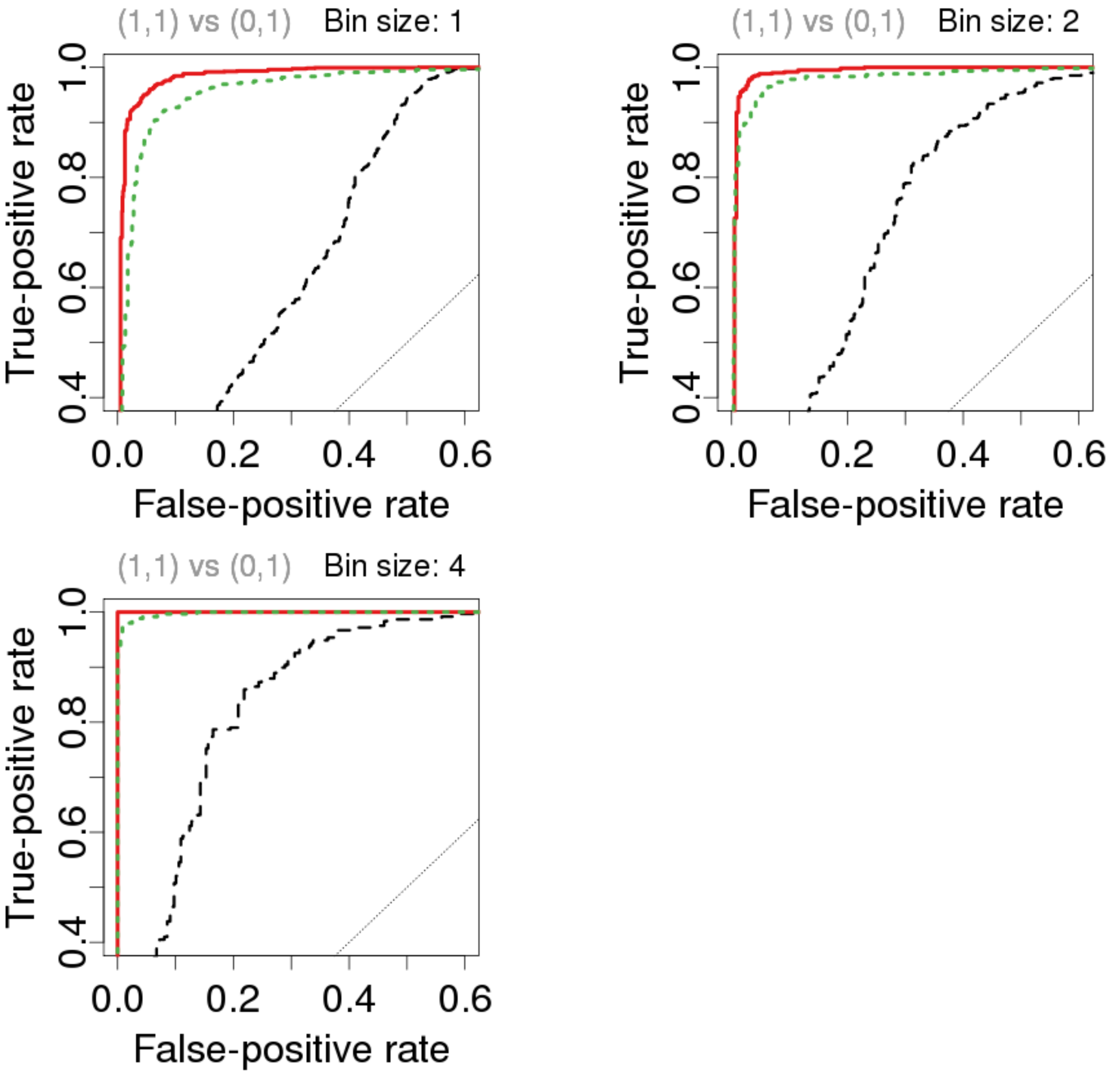

Figure 24: ROC curves for each preprocessing method at the full resolution as well as 2 different amounts of smoothing (using the mean() function) for region TCGA-02-0001:Chr13@0-70,cp=45+/-1,s=0/2. Legend: raw,NGC (dashed; #000000), TBN,NGC,NGC (solid; #E41A1C) and TCN,NGC (dotted; #4DAF4A).

6.4 (β_N, β_T) plots

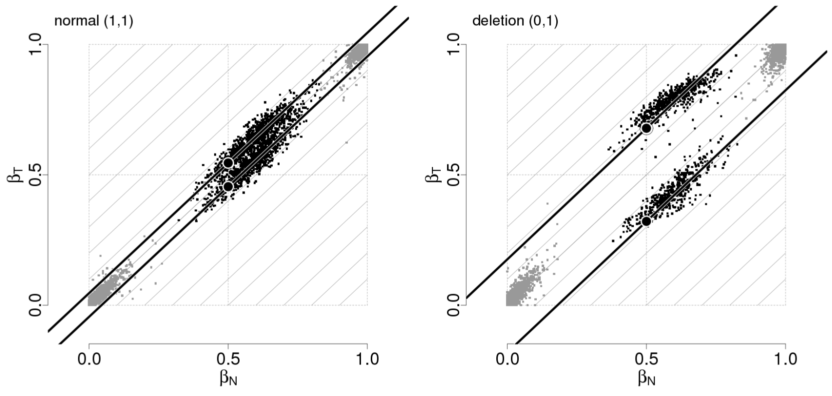

Figure 25: raw,NGC for region TCGA-02-0001:Chr13@0-70,cp=45+/-1,s=0/2.

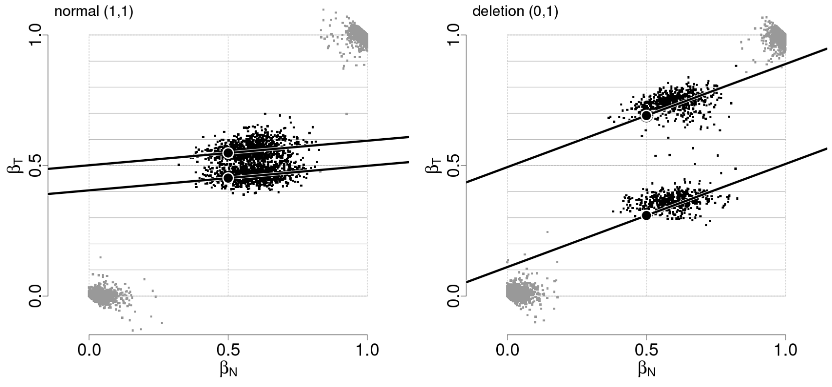

Figure 26: TBN,NGC,NGC for region TCGA-02-0001:Chr13@0-70,cp=45+/-1,s=0/2.

6.5 Allele-specific copy number estimates

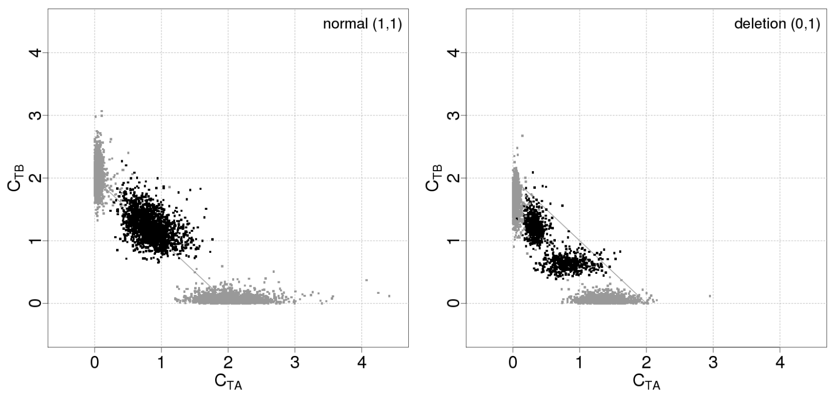

Figure 27: raw,NGC for region TCGA-02-0001:Chr13@0-70,cp=45+/-1,s=0/2.

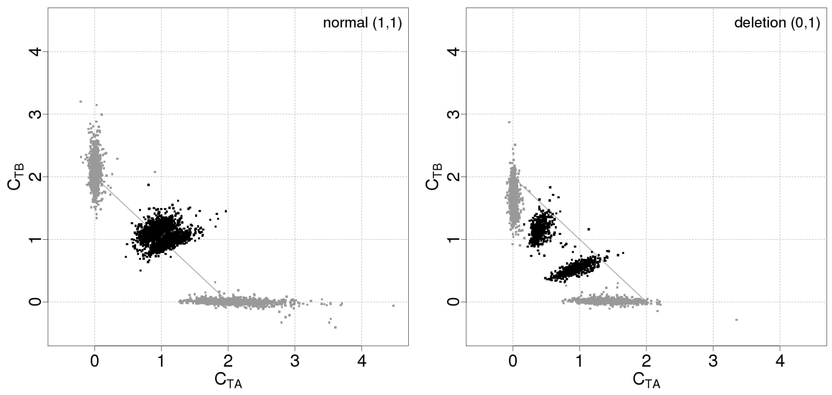

Figure 28: TBN,NGC,NGC for region TCGA-02-0001:Chr13@0-70,cp=45+/-1,s=0/2.

7 Bootstrap estimates of test statistics for all regions

	1/2	1/4	4/0	0/2
raw,NGC	17.727±1.122	4.391±0.865	5.349±1.014	10.598±1.099
TBN,NGC,NGC	43.255±1.734	15.400±1.175	31.300±2.534	32.108±1.391
TCN,NGC	29.564±1.428	3.896±0.980	11.456±1.317	25.812±1.370

Table 2: Student test statistics of the null hypothesis of equal mean before and after each PCN change point (heterozygous SNPs): raw or TumorBoost-normalized DH, and total copy number (last line). Mean ± standard deviation across 100 samplings of 225 points (for each PCN state) from the original data set. The larger value, the more different the true means are.

A Data files

A.1 Total copy numbers

$‘TCGA,GBM,BeadStudio,XY‘
AromaUnitTotalCnBinarySet:
Name: TCGA
Tags: GBM,BeadStudio,XY
Full name: TCGA,GBM,BeadStudio,XY
Number of files: 1
Names: TCGA-02-0001 [1]
Path (to the first file): rawCnData/TCGA,GBM,BeadStudio,XY/HumanHap550
Total file size: 2.14 MB
RAM: 0.00MB

A.2 Allele B fractions

$raw
AromaUnitFracBCnBinarySet:
Name: TCGA
Tags: GBM,BeadStudio,XY
Full name: TCGA,GBM,BeadStudio,XY
Number of files: 1
Names: TCGA-02-0001 [1]
Path (to the first file): totalAndFracBData/TCGA,GBM,BeadStudio,XY/HumanHap550
Total file size: 2.14 MB
RAM: 0.00MB

$‘TBN,NGC‘
AromaUnitFracBCnBinarySet:
Name: TCGA
Tags: GBM,BeadStudio,XY,TBN,NGC
Full name: TCGA,GBM,BeadStudio,XY,TBN,NGC
Number of files: 1
Names: TCGA-02-0001 [1]
Path (to the first file): totalAndFracBData/TCGA,GBM,BeadStudio,XY,TBN,NGC/HumanHap550
Total file size: 2.14 MB
RAM: 0.00MB

A.3 Genotype calls

$NGC
AromaUnitGenotypeCallSet:
Name: TCGA
Tags: GBM,BeadStudio,XY,NGC
Full name: TCGA,GBM,BeadStudio,XY,NGC
Number of files: 1
Names: TCGA-02-0001 [1]
Path (to the first file): callData/TCGA,GBM,BeadStudio,XY,NGC/HumanHap550
Total file size: 1.07 MB
RAM: 0.00MB

A.4 Genotype confidence scores

$NGC
AromaUnitSignalBinarySet:

Name: TCGA
Tags: GBM,BeadStudio,XY,NGC
Full name: TCGA,GBM,BeadStudio,XY,NGC
Number of files: 1
Names: TCGA-02-0001 [1]
Path (to the first file): callData/TCGA,GBM,BeadStudio,XY,NGC/HumanHap550
Total file size: 2.14 MB
RAM: 0.00MB

B Session information

This report was automatically generated using the R.rsp package.

- R version 2.10.1 (2009-12-14), `x86_64-unknown-linux-gnu`
- Locale: `LC_CTYPE=en_US.UTF-8`, `LC_NUMERIC=C`, `LC_TIME=en_US.UTF-8`, `LC_COLLATE=en_US.UTF-8`, `LC_MONETARY=C`, `LC_MESSAGES=en_US.UTF-8`, `LC_PAPER=en_US.UTF-8`, `LC_NAME=C`, `LC_ADDRESS=C`, `LC_TELEPHONE=C`, `LC_MEASUREMENT=en_US.UTF-8`, `LC_IDENTIFICATION=C`
- Base packages: base, datasets, graphics, grDevices, methods, stats, utils
- Other packages: aroma.cn.eval 0.1.1, aroma.core 1.5.0, aroma.light 1.15.1, digest 0.4.2, MASS 7.3-4, matrixStats 0.1.9, R.cache 0.2.0, RColorBrewer 1.0-2, R.filesets 0.8.0, R.menu 0.1.0, R.methodsS3 1.1.0, R.oo 1.6.7, R.rsp 0.3.6, R.utils 1.3.3, xtable 1.5-6
- Loaded via a namespace (and not attached): affxparser 1.18.0, tools 2.10.1
